# Aerobic Exercise and Weight Loss in Adults: A Systematic Review and Dose-Response Meta-Analysis

**DOI:** 10.1001/jamanetworkopen.2024.52185

**Published:** 2024-12-26

**Authors:** Ahmad Jayedi, Sepideh Soltani, Alireza Emadi, Mahdieh-Sadat Zargar, Ali Najafi

**Affiliations:** 1 Department of Epidemiology and Biostatistics, School of Public Health, Imperial College, London, United Kingdom; 2 Social Determinants of Health Research Center, Semnan University of Medical Sciences, Semnan, Iran; 3 Yazd Cardiovascular Research Center, Non-Communicable Diseases Research Institute, Shahid Sadoughi University of Medical Sciences, Yazd, Iran; 4 Food Safety Research Center (Salt), Semnan University of Medical Sciences, Semnan, Iran; 5 Clinical Research Development Unit, Kowsar Educational, Research and Therapeutic Hospital, Semnan University of Medical Sciences, Semnan, Iran; 6 Department of Internal Medicine, Imam Hossein Center For Education, Research and Treatment, Shahroud University of Medical Sciences, Shahroud, Iran

## Abstract

**Importance:**

Current guidance on the duration of aerobic exercise recommended in existing guidelines comes primarily from individual trials. Meta-analyses are lacking to examine the dose-response association of aerobic exercise with adiposity measures.

**Objective:**

To clarify the dose-response association of aerobic exercise with adiposity measures.

**Data Sources:**

PubMed, Scopus, the Cochrane Central Register of Controlled Trials, and gray literature sources (ProQuest and ClinicalTrials.gov) from inception to April 30, 2024.

**Study Selection:**

Randomized clinical trials with intervention durations of at least 8 weeks evaluating the effects of supervised aerobic training on adults with overweight or obesity.

**Data Extraction and Synthesis:**

The PRISMA guidelines were followed to report the results of the meta-analysis. Data extraction was conducted by 2 teams of 2 reviewers each, working independently and in duplicate. Random-effects meta-analyses were performed to estimate mean differences and 95% CIs for each 30-minute per week aerobic exercise and to clarify the shape of the curvilinear associations.

**Main Outcomes and Measures:**

Measures of body weight, waist circumference, body fat, adverse events, medication use reduction, and health-related quality of life score. The certainty of evidence was assessed using the Grading of Recommendations Assessment, Development and Evaluation (GRADE) tool, with a range from very low to high certainty.

**Results:**

In total, 116 randomized clinical trials involving 6880 participants (4199 [61%] female; mean [SD] age, 46 [13] years) with overweight or obesity were included. Each 30 minutes per week of aerobic exercise was associated with reduced body weight by 0.52 kg (95% CI, −0.61 to −0.44 kg; n = 109 trials, GRADE = moderate), waist circumference by 0.56 cm (95% CI, −0.67 to −0.45 cm; n = 62 trials, GRADE = high), body fat percentage by 0.37% (95% CI, −0.43% to −0.31%; n = 65 trials, GRADE = moderate), as well as the areas of visceral (mean difference, −1.60 cm^2^ [95% CI, −2.12 to −1.07 cm^2^]; n = 26 trials, GRADE = high) and subcutaneous (mean difference, −1.37 cm^2^ [95% CI, −1.82 to −0.92 cm^2^]; n = 27 trials, GRADE = moderate) adipose tissues. Aerobic exercise was associated with modestly increased physical (standardized mean difference, 1.69 SD [95% CI, 1.18-2.20 SD]) and mental (standardized mean difference, 0.74 SD [95% CI, 0.29-1.19 SD]) aspects of quality of life (1 trial with 80 participants, GRADE = low). It was associated with modestly increased mild to moderate adverse events, which were mostly musculoskeletal symptoms (risk difference, 2 more events per 100 participants [95% CI, 1 to 2 more]; GRADE = low). Dose-response meta-analyses indicated that body weight, waist circumference, and body fat measures decreased linearly or monotonically in association with increasing duration of aerobic exercise to 300 minutes per week, with aerobic exercise lasting 150 minutes per week at moderate to vigorous intensities resulting in clinically important reductions in waist circumference and body fat.

**Conclusions and Relevance:**

In this meta-analysis of randomized clinical trials, engaging in 30 minutes of aerobic exercise per week was associated with modest reductions in body weight, waist circumference, and body fat measures among adults with overweight or obesity. However, aerobic training exceeding 150 minutes per week at moderate intensity or greater may be needed to achieve clinically important reductions.

## Key Points

**Question:**

What is the dose-response association between aerobic exercise and measurements of body weight, waist circumference, and body fat?

**Findings:**

This meta-analyses of 116 randomized clinical trials involving 6880 adults with overweight or obesity found that levels of body weight, waist circumference, and body fat decreased linearly or monotonically with increasing duration of aerobic exercise at moderate to vigorous intensities to 300 minutes per week. Aerobic exercise at least 150 minutes per week was associated with clinically important reductions in waist circumference and measures of body fat.

**Meaning:**

These results suggest that aerobic exercise training at least 150 minutes per week at moderate intensity or higher may be needed to achieve clinically important reductions in waist circumference and measures of body fat.

## Teaser

This dose-response meta-analysis of randomized clinical trials assesses the association of aerobic exercise duration and intensity with measures of adiposity among adults with overweight or obesity.

## Introduction

Overweight and obesity are among the most important health problems worldwide.^1^ During the past 45 years, the prevalence of overweight and obesity has tripled globally, resulting in approximately 50% of adults having excess body weight.^2^ Current guidelines recommend that exercise programs, mainly in the form of aerobic exercise or aerobic and resistance exercises combined, should be one of the key elements of lifestyle modification programs designed to manage obesity.^3,4,5,6,7^

It is generally recommended in the guidelines that at least 150 minutes per week of aerobic exercise at moderate intensity is required to achieve clinically important weight loss.^3,4,5,6,7^ The American College of Sport Medicine suggested that exercise programs shorter than 150 minutes per week promote minimal weight loss, and that exercise programs at least 150 minutes per week are required for modest weight loss of 2 to 3 kg.^3^ The guideline also recommended that an exercise program of 225 to 420 minutes per week at moderate intensity is required for weight loss of 5.0 to 7.5 kg.^3^ However, current guidance on the duration of aerobic exercise programs recommended in existing guidelines comes primarily from individual trials and is predominantly from older data.

Although previous meta-analyses of randomized clinical trials have provided a wealth of evidence that aerobic exercise is an effective intervention for weight loss in adults with overweight or obesity, the results of these meta-analyses are mainly based on pairwise comparisons between intervention and control groups.^8,9,10^ In fact, systematic reviews and meta-analyses are lacking to examine the possible dose-response association of aerobic exercise with body weight, waist size, and fat. Evaluating the potential dose-response association is of clinical and public health importance^11^ because it can determine how body weight, waist size, and fat change with increasing aerobic training dose, thereby contributing to providing useful information needed for decision-making. Therefore, our aim was to perform a systematic review and meta-analysis of randomized clinical trials to investigate the possible dose-response association of aerobic exercise of varying intensity with measures of body weight, waist size, and fat in adults with overweight or obesity.

## Methods

We reported the meta-analysis according to the Preferred Reporting Items for Systematic Reviews and Meta-Analyses (PRISMA) 2020 statement for systematic reviews of interventions. The protocol of the present systematic review was registered with PROSPERO (CRD42023460474).^12^ Given that this is a review study, we were not required to secure approval for the study protocol from an ethics committee or institutional review board, nor was informed consent from study participants necessary.

### Systematic Search

PubMed, Scopus, the Cochrane Central Register of Controlled Trials, and gray literature sources (ProQuest and ClinicalTrials.gov) were selected for the systematic search from inception until April 30, 2024. See eMethods 1 in Supplement 1 for additional information and eTable 1 in Supplement 1 for the search strategy.

### Study Selection

Using our a priori protocol,^12^ we searched for randomized clinical trials that (1) had an intervention duration of at least 8 weeks^13^; (2) included adults older than 18 years with either overweight or obesity (defined as body mass index [BMI, calculated as weight in kilograms divided by height in meters squared] >25 in Western countries^6^ and >23 in Asian countries^14^) or central obesity; (3) conducted a supervised continuous aerobic training program (eg, walking, running) as an intervention; (4) reported the efficacy of aerobic exercise on 1 of the study outcomes (mean [SD] of change for continuous outcomes, number of events for binary outcomes); and (5) reported frequency (sessions per week), duration (minutes per session), and intensity (moderate, vigorous, or combined) of aerobic exercise in the intervention arm. Detailed information on criteria applied for excluding studies is provided in eMethods 1 in Supplement 1.

### Outcomes

We considered body weight change (in kg) and adverse events as our primary outcomes. Secondary outcomes included changes in waist circumference (in cm), body fat percentage, fat mass (in kg), area of visceral adipose tissue (in cm^2^), area of subcutaneous adipose tissue (in cm^2^), health-related quality of life score, and medication reduction (eg, antidiabetic or antihypertensive medications).

### Data Extraction and Risk of Bias Assessment

Data extraction was conducted by 2 teams of 2 reviewers (A.J. and S.S.; A.E. and M.-S.Z.) working independently and in duplicate. Detailed information on data extraction and risk of bias assessment is provided in eMethods 2 in Supplement 1. We assessed risk of bias of the trials using version 2 of the Cochrane risk of bias tool.^15^

### Intensity of Aerobic Exercise Programs

The intensity of aerobic exercise programs in the included trials was classified as follows^16^: (1) light (1.6 to <3.0 metabolic equivalents [METs], or 40% to <55% maximum heart rate, or 20% to <40% maximum oxygen consumption [V̇o_2_max]); (2) moderate (3.0 to <6.0 METs, or 55% to <70% maximum heart rate, or 40% to <60% V̇o_2_max); and (3) vigorous (6.0 to <9.0 METs, or 70% to <90% maximum heart rate, or 60% to <85% V̇o_2_max). Programs of moderate to vigorous intensity were those that included both moderate and vigorous categories.

### Statistical Analysis

We used a random-effects model (DerSimonian and Laird method)^17^ to calculate summary effect estimates for primary and secondary outcomes. We considered the mean difference and its 95% CI as the effect size for continuous outcomes. The exception was health-related quality of life, for which we calculated standardized mean differences in scores. For binary outcomes (adverse events and medication reduction), we reported measures of relative (risk ratio) and absolute (risk difference) risks. Two-sided *P* < .05 was deemed to be statistically significant.

For continuous outcomes, we first calculated changes (mean and SD of change) from baseline values in each study arm. We computed the mean and SD of changes for the trials that did not report this information by using information from preintervention and postintervention measures.^18^ Second, we followed the method introduced by Crippa and Orsini^19^ to assess mean differences and their corresponding 95% CIs for each 30-minute per week increment in aerobic exercise.^20^ Trial-specific effect estimates were combined using a random-effects model.^17^ The number of participants, the mean (SD) of the change in continuous outcomes, and the dose (minutes per week) of aerobic exercise across study arms were required for this method.

We performed predefined subgroup analyses based on the intensity of aerobic exercise, health status of the participants, and study risk of bias. We used meta-regression analysis to calculate the *P* value for the subgroup difference. The Instrument to Assess the Credibility of Effect Modification Analyses (ICEMAN)^21^ recently introduced 8 criteria that we used to determine whether or not subgroup differences were credible. We applied the ICEMAN tool to assess the credibility of subgroup differences when the *P* value for the group difference was lower than .10.^21^ eTable 2 in Supplement 1 presents the domains of the ICEMAN tool and each domain was evaluated.

We also performed post hoc subgroup analyses based on geographical location, intervention duration, sex, baseline weight status, type of aerobic exercise, exercise frequency, exercise modality, degree of adherence to the intervention program, dropout, and the existence of calorie restriction as a co-intervention.

Finally, to investigate the dose-response association of aerobic exercise duration (minutes per week) with our continuous outcomes, we conducted a nonlinear dose-response meta-analysis.^19^ Publication bias was tested using the Egger test (*P* value <.10)^22^ and inspection of the funnel plot. We assessed heterogeneity using the *I*^2^ statistic and performed a χ^2^ test for homogeneity.^23^ Statistical analyses were conducted using STATA software, version 17.0 (StataCorp LLC).

### Grading the Evidence

Two authors (A.J. and M.-S.Z.) judged the certainty of evidence according to the Grading of Recommendations Assessment, Development and Evaluation (GRADE) tool, with a range from very low to high certainty.^24^ Detailed information on the domains of the GRADE tool is provided in eMethods 3 in Supplement 1.

## Results

### Literature Search and Study Selection Process

The flowchart of the systematic search is given in eFigure 1 in Supplement 1. We removed 8975 duplicates, leaving 21 518 records for screening of the title and abstract. At this step, we excluded 21 163 records. We read the full text of 351 records and, finally, 116 trials with 6880 participants (4199 [61%] female and 2681 [39%] male; mean [SD] age, 46 [13] years) with overweight or obesity were eligible for inclusion. We excluded 235 full texts for reasons presented in eTable 3 in Supplement 1.

### Characteristics of Trials Included in the Meta-Analysis

eTable 4 in Supplement 1 gives the characteristics of the trials included in the meta-analysis.^25,26,27,28,29,30,31,32,33,34,35,36,37,38,39,40,41,42,43,44,45,46,47,48,49,50,51,52,53,54,55,56,57,58,59,60,61,62,63,64,65,66,67,68,69,70,71,72,73,74,75,76,77,78,79,80,81,82,83,84,85,86,87,88,89,90,91,92,93,94,95,96,97,98,99,100,101,102,103,104,105,106,107,108,109,110,111,112,113,114,115,116,117,118,119,120,121,122,123,124,125,126,127,128,129,130,131,132,133,134,135,136,137,138,139,140^ All trials implemented a supervised aerobic exercise program. The control groups received no intervention or continued a sedentary lifestyle or maintained their usual activity. In brief, 48 trials were conducted in North America, 39 in Asia, 18 in Europe, 5 in Australia, 4 in South America, and 2 in Africa. Forty-seven trials were conducted with female participants, 30 with male participants, and 39 with both sexes. Among the trials, 42 involved participants with obesity, 2 involved participants with overweight, and the remaining 72 trials involved mixed populations. There were 41 trials that involved patients with overweight or obesity and a comorbidity (eg, type 2 diabetes, hypertension, or fatty liver disease) and 75 trials that involved otherwise healthy people. The mean age of the participants in the included trials ranged from 19 to 74 years, and the mean BMI ranged from 25 to 44 (mean [SD], 31 [3]). Among the trials, 78 implemented a progressive aerobic training program, and 38 implemented a nonprogressive training program. The duration of the intervention (minutes of aerobic exercise per week) ranged from 55 to 300 minutes per week (mean [SD], 167 [54] minutes per week). The degree of adherence to the intervention program was excellent, good, or high, or at least 80% in 48 trials and less than 80% in 6 trials. In 60 trials, no information was provided on the level of adherence. The dropout rate was lower than 20% in 80 trials and 20% or higher in 27 trials (eTable 5 in Supplement 1). Adverse events were mostly mild to moderate musculoskeletal symptoms, including knee and ankle injury, pain in the knee or back, and arthritis, as well as an increase in blood pressure in 1 trial (eTable 5 in Supplement 1). Fourteen trials were at low risk of bias, 48 trials had some level of concern, and 54 trials were at serious risk of bias (eTable 6 in Supplement 1).

### Primary Outcomes

There were 109 trials with 6298 participants that reported on body weight.^26,27,29,30,31,32,33,34,35,36,37,38,39,40,41,42,43,44,45,46,47,48,49,51,52,53,55,56,57,58,59,60,61,62,63,64,65,66,67,68,69,70,71,72,73,74,75,76,77,78,79,80,81,82,83,84,85,86,87,88,89,90,91,92,93,94,95,96,97,98,99,100,101,102,103,104,105,106,107,108,110,112,114,115,116,117,118,119,120,121,122,123,124,125,126,127,128,129,130,131,132,133,134,135,136,137,138,139,140^ Each 30 minutes per week of aerobic exercise was associated with body weight reduced by 0.52 kg (95% CI, −0.61 to −0.44; *I*^2^ = 88%; GRADE = moderate) (Table). A forest plot was not provided because there were too many trials. Aerobic exercise was associated with increased mild to moderate adverse events by 2 more events per 100 patients (95% CI, 1 to 2 more; 9 trials; GRADE = low) (eFigures 2 and 3 in Supplement 1; Table).^34,38,44,45,55,71,119,130,140^ Aerobic exercise was not associated with an increase in hypoglycemic reactions (risk difference, 1 more per 100 patients [95% CI, 1 fewer to 3 more]; 3 trials; GRADE = very low]) (eFigures 4 and 5 in Supplement 1; Table).^38,71,119^

**Table.** Association of Supervised Aerobic Exercise With Body Weight, Waist Circumference, and Body Fat Among Participants With Overweight or Obesity

Outcome	Anticipated absolute effect (95% CI)		Relative effect, RR (95% CI)	No. of participants/No. of studies	Certainty of the evidence (GRADE)
	Risk with control group	Risk with intervention, No. of events per 1000 participants			
Body weight	0.52 (0.61-0.44) kg Lower than control	NA	NA	6298/109	Moderate
Waist circumference	0.56 (0.67-0.45) cm Lower than control	NA	NA	4281/62	High
Body fat percentage	0.37% (0.43%-0.31%) Lower than control	NA	NA	3466/65	Moderate
Body fat mass^a^	0.20 (0.32-0.08) kg Lower than control	NA	NA	410/7	High
Visceral adipose tissue area	1.60 (2.12-1.07) cm^2^ Lower than control	NA	NA	1501/26	High
Subcutaneous adipose tissue area	1.37 (1.82-0.92) cm^2^ Lower than control	NA	NA	1634/27	Moderate
Adverse events	27 Events per 1000 participants	46 (27-79)	1.74 (1.02-2.97)	1170/9	Low
Hypoglycemia	6 Events per 1000 participants	15 (3-77)	2.51 (0.49-12.78)	335/3	Very low
Medication use reduction	36 Events per 1000 participants	52 (21-131)	1.43 (0.57-3.60)	587/2	Low
Health-related quality of life mental score	1.69 (SD, 1.18-2.20) Points higher than control	NA	NA	80/1	Low
Health-related quality of life physical score	0.74 (SD, 0.29-1.19) Points higher than control	NA	NA	80/1	Low

Abbreviations: GRADE, Grading of Recommendations Assessment, Development and Evaluation (range is very low, low, moderate, and high); NA, not applicable; RR relative risk.

^a^ There was significant and credible difference based on study risk of bias, where trials with a low risk of bias reported a smaller effect; results of trials with a low risk of bias are presented.

### Secondary Outcomes

A summary of the association of aerobic exercise with body waist circumference and measures of fat is given in the Table. Each 30 minutes per week of aerobic exercise was associated with lower waist circumference (mean difference, −0.56 cm [95% CI, −0.67 to −0.45 cm]; *I*^2^ = 88%; GRADE = high; for 62 trials with 4281 participants) (eFigure 6 in Supplement 1),^25,26,32,34,37,39,40,41,45,46,47,49,54,55,59,61,62,64,65,66,69,71,73,76,77,78,79,80,81,82,83,91,92,93,94,95,97,102,104,105,106,108,109,110,111,112,113,114,116,117,118,119,122,123,125,128,132,136,137,139^ lower body fat percentage (mean difference: −0.37% [95% CI, −0.43% to −0.31%]; *I*^2^ = 83%; GRADE = moderate; for 65 trials with 3466 participants) (eFigure 7 in Supplement 1),^26,27,28,30,31,33,34,35,36,38,40,41,42,44,45,46,48,51,56,58,59,62,63,65,73,74,75,76,77,78,79,80,84,86,90,93,96,98,99,100,101,104,105,108,109,114,116,117,119,121,123,126,127,128,129,131,132,134,135,136,137,138,139,140^ and lower body fat mass (mean difference, −0.20 kg [95% CI, −0.32 to −0.08 kg]; *I*^2^ = 26%; GRADE = high; for 7 trials with a low risk of bias and 410 participants) (eFigure 8 in Supplement 1).^55,61,65,69,79,112,138^ There was also moderate to high certainty that aerobic exercise was associated with lower visceral (mean difference, −1.60 cm^2^ [95% CI, −2.12 to −1.07 cm^2^]; n = 26 trials, GRADE = high)^47,50,51,53,56,66,69,75,76,78,79,81,82,83,89,91,99,110,117,119,120,135,136,137,138,140^ and subcutaneous (mean difference, −1.37 cm^2^ [95% CI, −1.82 to −0.92 cm^2^]; n = 27 trials, GRADE = moderate)^47,51,53,56,63,66,69,75,79,81,82,83,89,91,93,98,99,110,114,117,119,120,135,136,137,138,140^ adipose tissues (eFigures 9 and 10 in Supplement 1; Table). Aerobic exercise was not associated with lower antidiabetic^34,119^ and antihypertensive^34^ medication use (risk difference, 1 more per 100 patients [95% CI, 2 fewer to 5 more; 2 trials; GRADE = low) (eFigures 11 and 12 in Supplement 1) but was associated with modestly increased physical (standardized mean difference, 1.69 SD [95% CI, 1.18-2.20 SD] and mental (standardized mean difference, 0.74 SD [95% CI, 0.29-1.19 SD] aspects of quality of life (1 trial with 80 participants, GRADE = low) (eFigures 13 and 14 in Supplement 1; Table).^130^

### Subgroup Analyses

We considered 3 plausible effect modifiers, including risk of bias, intensity of aerobic exercise, and presence of comorbidity in the study participants, and applied the ICEMAN criteria to identify credible subgroup differences with *P* < .10 for the group difference^21^ (eTables 7-18 in Supplement 1). There was no significant or credible difference across subgroups defined based on study risk of bias, except for body fat mass, for which trials with a low risk of bias reported smaller effects than those with some concerns or high risk of bias (*P* = .007 for subgroup difference; ICEMAN credibility = moderate). Therefore, we reported the results of the trials with a low risk of bias. In addition, we conducted a series of post hoc subgroup analyses to identify potential sources of heterogeneity and potential effect modifiers. Although there were some significant differences between some subgroups, further detailed evaluation based on the 8 criteria introduced by ICEMAN suggested that there was no credible difference across post hoc subgroup analyses (eTables 7-18 in Supplement 1). For example, we found that trials that implemented a progressive aerobic training indicated greater association with weight reduction (mean difference, −0.58 kg [95% CI, −0.69 to −0.46 kg]; 73 trials) compared with trials that implemented a nonprogressive exercise (mean difference, −0.39 kg [−0.50 to −0.28]; 36 trials); however, the credibility of this subgroup difference was rated low as this subgroup was not based on an a priori hypothesis and change may be a likely explanation (*P* = .02 for subgroup difference). Interestingly, there were no significant differences among subgroups categorized by the duration of the intervention (8 to ≤12, 12-24, and >24 weeks) for most outcomes, except for visceral and subcutaneous adipose tissue areas, where short-term trials (8 to ≤12 weeks) indicated greater associations with reduction in visceral (*P* = .004 for subgroup difference) and subcutaneous (*P* = .02 for subgroup difference) adipose tissue areas than trials with longer duration (12-24 and >24 weeks).

### Nonlinear Dose-Response Meta-Analyses

**Figure 1.**
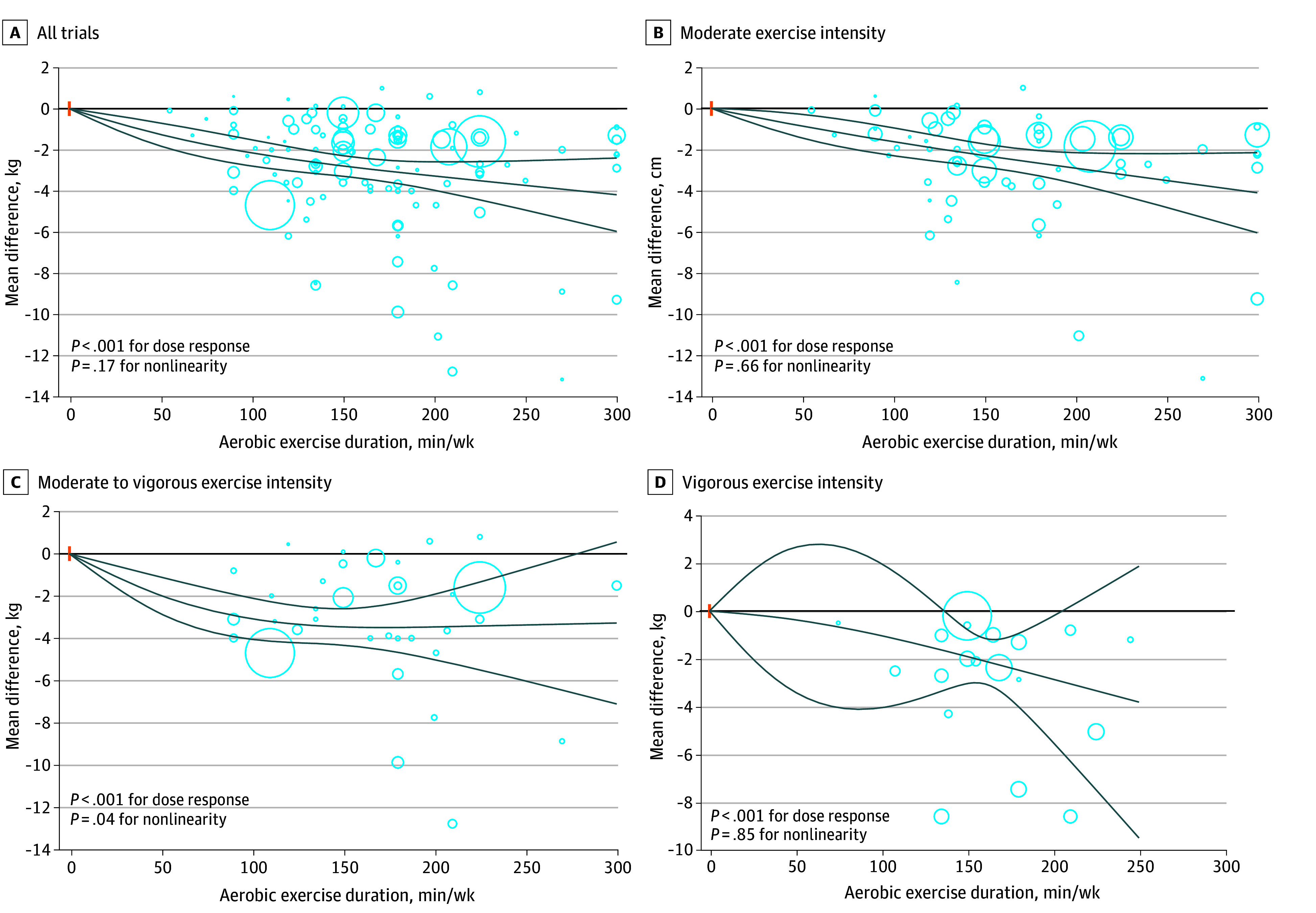
Dose-Response Association of Aerobic Exercise With Body Weight Among Adults With Overweight or Obesity Solid lines represent the dose-response lines, and lines above and below are 95% CIs. Circles represent relative risk point estimates for aerobic exercise from each study, with circle size proportional to the inverse of standard error. The vertical orange lines represent the baseline aerobic exercise dose across studies.

**Figure 2.**
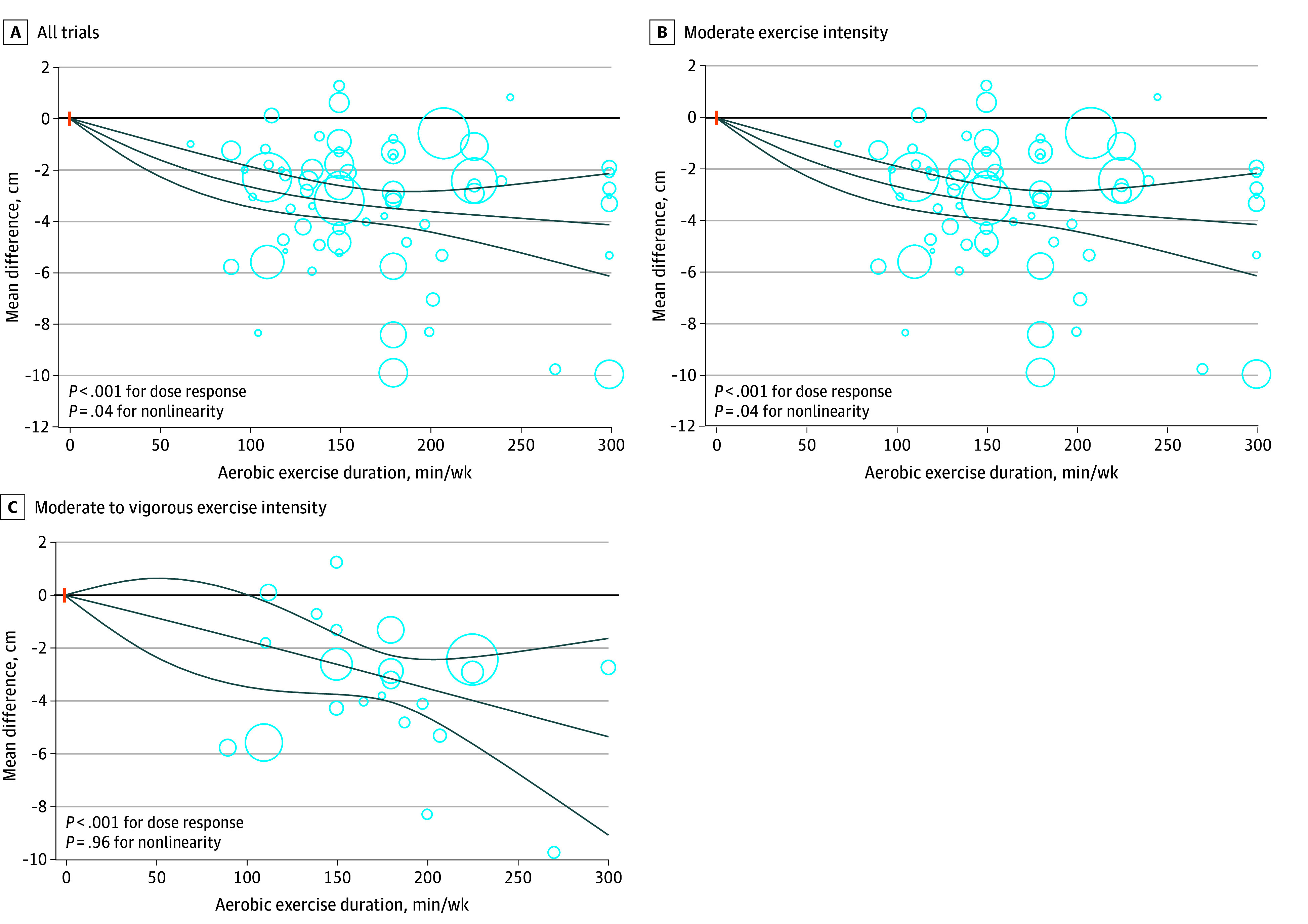
Dose-Response Association of Aerobic Exercise With Waist Circumference Among Adults With Overweight or Obesity Solid lines represent the dose-response lines, and lines above and below are 95% CIs. Circles represent relative risk point estimates for aerobic exercise from each study, with circle size proportional to the inverse of the standard error. The vertical orange lines represent the baseline aerobic exercise dose across studies.

In the main analysis, our dose-response meta-analysis suggested a linear reduction in body weight associated with increasing duration of aerobic exercise to 300 minutes per week (*P* = .17 for nonlinearity; *P* < .001 for dose response; n = 109 trials) (Figure 1). The degree of weight loss was −2.79 kg (95% CI, −3.29 to −2.29 kg) at 150 minutes per week and −4.19 kg (95% CI, −5.98 to −2.41 kg) at 300 minutes per week (eTable 19 in Supplement 1). A similar linear reduction in body weight was also observed in the dose-response analyses of trials with moderate, moderate to vigorous, and vigorous exercise intensities (Figure 1; eTable 19 in Supplement 1). For waist circumference, there was a nonlinear reduction associated with dose of aerobic exercise in the main analysis (*P* = .04 for nonlinearity; *P* < .001 for dose response; n = 62 trials) (Figure 2); however, the analysis of trials with moderate to vigorous intensity showed a linear reduction in waist circumference associated with the dose of aerobic exercise. The degree of reduction in waist circumference was −4.21 cm (95% CI, −6.85 to −1.58 cm) at 300 minutes per week of aerobic exercise at moderate intensity and −5.34 cm (95% CI, −9.05 to −1.63 cm) at 300 minutes per week of aerobic exercise at moderate to vigorous intensity (eTable 19 in Supplement 1). The results suggested that the association of aerobic exercise with waist circumference surpassed the threshold set as the minimum clinically important difference for waist circumference (2 cm), with higher duration indicating greater reduction in waist circumference.

**Figure 3.**
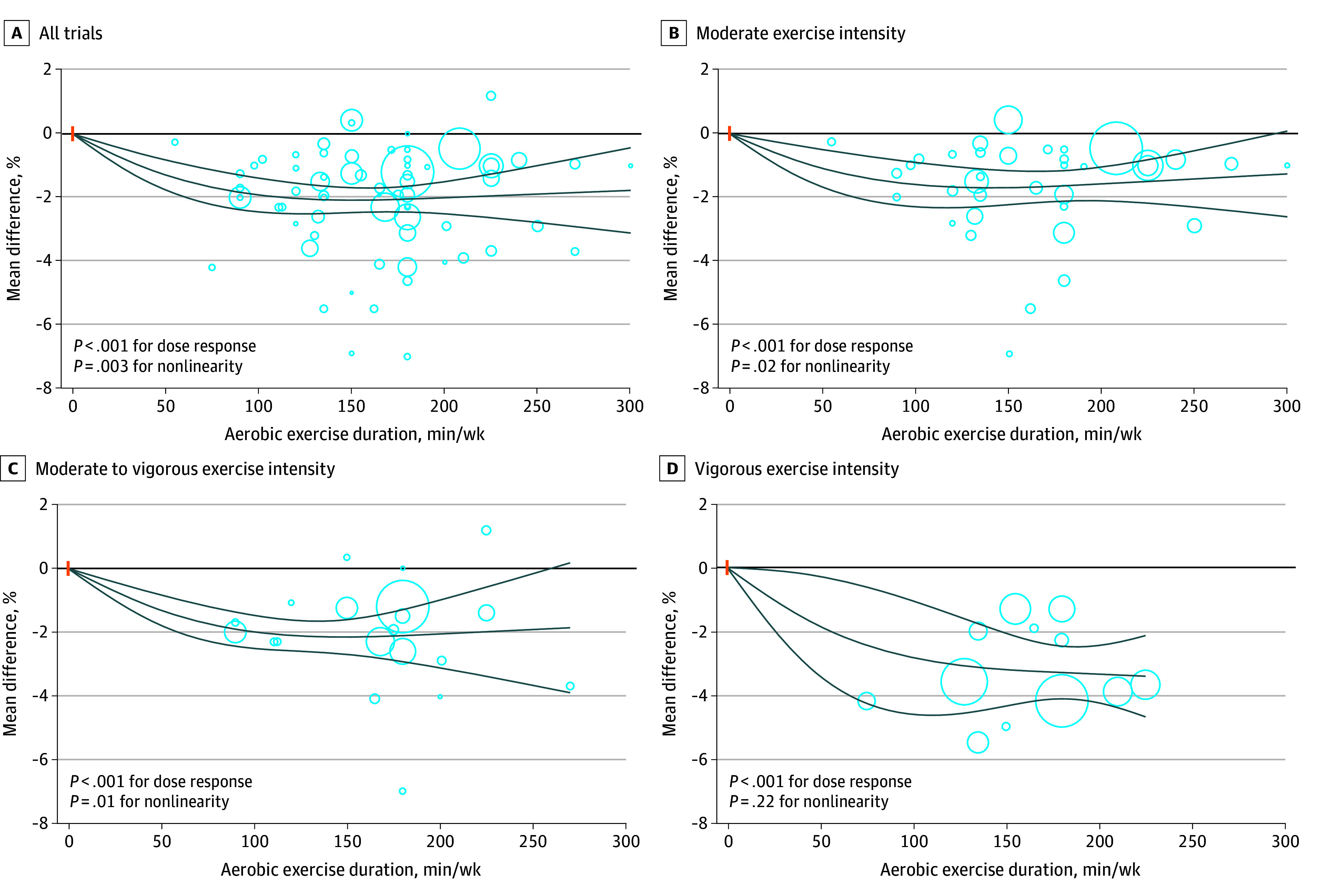
Dose-Response Association of Aerobic Exercise With Body Fat Percentage Among Adults With Overweight or Obesity Solid lines represent the dose-response lines, and lines above and below are 95% CIs. Circles represent relative risk point estimates for aerobic exercise from each study, with circle size proportional to the inverse of the standard error. Small vertical orange lines represent the baseline aerobic exercise dose across studies.

**Figure 4.**
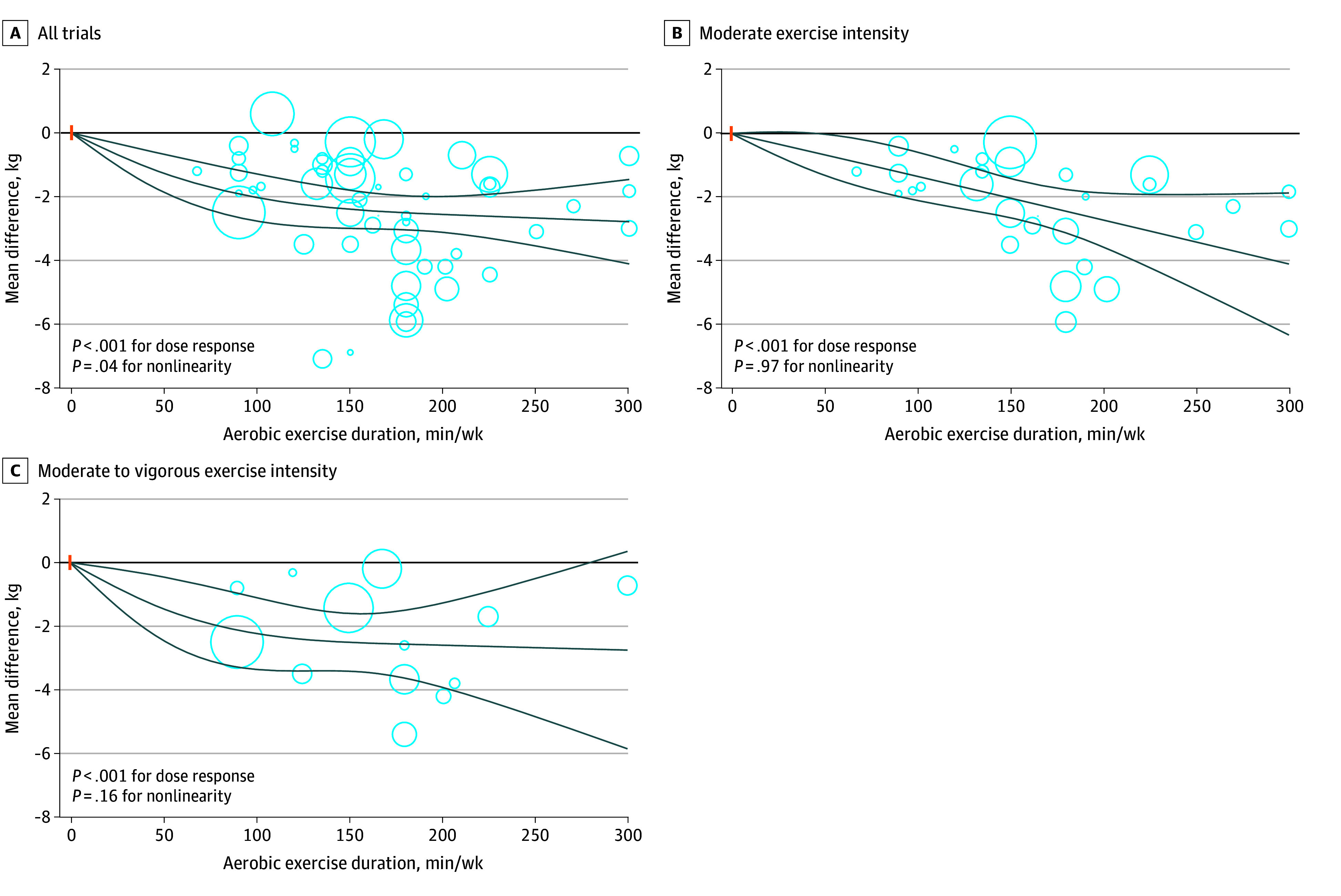
Dose-Response Association of Aerobic Exercise With Fat Mass Among Adults With Overweight or Obesity Solid lines represent the dose-response lines, and lines above and below are 95% CIs. Circles represent relative risk point estimates for aerobic exercise from each study, with circle size proportional to the inverse of the standard error. The vertical orange lines represent the baseline aerobic exercise dose across studies.

For body fat percentage associated with dose of aerobic exercise, the greatest reduction was observed at 150 minutes per week (mean difference, −2.08% [95% CI, −2.47% to −1.69%]) (Figure 3; eTable 19 in Supplement 1), surpassing the threshold (2%) as the minimum clinically important difference for body fat percentage. Similar findings were observed for body fat mass associated with dose of aerobic exercise, in which the size of the effect was larger than the minimum clinically important difference threshold (2 kg) at 100 minutes per week in the main analysis (mean difference, −2.03 kg [95% CI, −2.77 to −1.29 kg]) and in the analysis of trials with moderate to vigorous exercise intensity (mean difference, −2.23 kg [95% CI, −3.36 to −1.10 kg]) (Figure 4; eTable 19 in Supplement 1). The dose-dependent associations of aerobic exercise with areas of visceral and subcutaneous adipose tissues are indicated in eFigures 15 and 16 and eTable 19 in Supplement 1.

### Publication Bias

It is possible that the associations were overestimated due to the asymmetry observed in the funnel plots for body fat percentage (Egger test = 0.003) and subcutaneous adipose tissue area (Egger test, <0.001). For the other outcomes, the funnel plots showed no signs of asymmetry (eFigures 17-22 in Supplement 1). For body fat precentage, trim-and-fill analysis found 1 potentially missing study, and the main effect size did not change after imputing this study (mean differemce, −0.37% [95% CI, −0.43% to −0.30%]; 66 trials). For subcutaneous adipose tissue, trim-and-fill analysis found 11 potentially missing studies, and the main effect size attenuated but remained significant after imputing these studies (mean difference, −1.03 cm^2^ [95% CI, −1.46 to −0.60 cm^2^]; 38 trials).

### Grading the Evidence

The certainty of evidence was rated moderate for body weight due to downgrades for imprecision (effect size smaller than the minimum clinically important difference) and study risk of bias and an upgrade for the dose-response gradient. The certainty of evidence was also downgraded for publication bias and serious risk of bias for body fat percentage (eTable 20 in Supplement 1).

## Discussion

To answer an important question that is relevant to patients and practice, this meta-analysis of randomized clinical trials gathered the available data assessing the associations of aerobic exercise with body weight, waist circumference, and body fat. Our findings suggested that each 30 minutes of aerobic exercise per week may help adults who have overweight or obesity slightly reduce body weight, waist circumference, and measures of fat. Our nonlinear dose-response meta-analyses indicated that body weight decreased linearly in association with increasing duration of aerobic exercise up to 300 minutes per week at a variety of intensities. Relatively similar findings were found for waist circumference, with waist circumference decreasing linearly or monotonically in association with increasing duration of aerobic exercise. The analyses of body fat percentage suggested a nonlinear association, with greatest improvement associated with 150 minutes per week. There was low certainty of evidence that aerobic exercise was associated with increased health-related quality of life score.

A recent network meta-analysis compared different types of long-term (≥6 months) exercise and concluded that aerobic exercise is the most effective exercise associated with weight loss in adults with obesity.^13^ Their findings indicated that, when compared with no intervention, aerobic exercise was associated with reduced body weight by 2.18 kg and waist circumference by 2.33 cm.^13^ According to pairwise meta-analyses, aerobic exercise was associated with reduced waist circumference by 2.12 to 3.20 cm^8,10^ and body weight by 1.60 to 2.00 kg.^9,10^ The greatest number of trials included in the earlier reviews were 45 trials for body weight^141^ and 25 trials for waist circumference.^8^ We included 109 trials assessing body weight and 62 trials assessing waist circumference, enabling us to provide the most comprehensive review in this area. Additionally, our nonlinear dose-response meta-analyses enabled us to show the dose and intensity of exercise associated with the greatest improvements in body weight, waist circumference, and fat percentage, which were not reported in previous reviews. For instance, the range of weight loss in a previous review was between 1.60 and 2.39 kg, and the range of waist circumference loss was from 2.12 to 3.70 cm.^9^ By comparison, we found a 4.19 kg decrease in body weight associated with 300 minutes of aerobic exercise per week, and decreases in waist circumference associated with 300 minutes of aerobic exercise per week of 4.21 cm for moderate intensity and 5.34 cm for moderate to vigorous intensity.

Our findings indicated that body weight and waist circumference decreased linearly or monotonically in association with increasing duration of aerobic exercise at different intensities. Such a linear reduction was also found associated with body fat mass and subcutaneous adipose tissue area. The results suggest that longer durations of aerobic exercise are associated with greater reductions in body weight, waist circumference, and fat tissue. The nonlinear dose-response analyses indicated that aerobic exercise at least 150 minutes per week was associated with clinically important reductions in waist circumference and measures of body fat; thus, aerobic training at least 150 minutes per week may be needed to achieve important reductions in waist circumference and body fat. Point-specific estimates for different aerobic exercise duration and intensity can help patients and health care professionals select the optimal aerobic exercise duration and intensity according to their weight loss goals.

A series of analyses was conducted to assess the associations across various subgroups. In terms of modality, progressive exercise demonstrated greater association with weight reduction compared with nonprogressive exercise; however, the credibility of this subgroup difference was rated low and the analyses of other outcomes did not reveal any significant subgroup difference. The analyses of body fat percentage, waist circumference, and visceral adipose tissue area indicated that the size of the effect surpassing the minimum clinically important difference threshold was larger with vigorous exercise compared with light or moderate exercise intensities, suggesting better outcomes with more vigorous exercise. The findings were largely consistent among subgroups categorized by the duration of the intervention (8-12, 12-24, and >24 weeks). However, only 2 trials reported on intervention durations longer than 48 weeks,^88,140^ both of which showed small mean differences. This raises concerns about the association of aerobic exercise with weight loss beyond 1 year.

Our findings did not indicate any significant reduction in medication use associated with aerobic exercise. This finding could be because only 2 trials were included in the analysis^34,119^ and the follow-up period was relatively short (12 to 16 weeks). A recent meta-analysis found that aerobic exercise was associated with a modest reduction in medication use among patients with type 2 diabetes.^20^

### Limitations

There were several shortcomings of this study that need further evaluation in future research and should be considered when interpreting the results. First, there was high heterogeneity in the data, which may limit the generalizability of the findings. However, there were many trials included in almost all analyses. In these situations, even a slight variation in the effect estimates can lead to substantial data heterogeneity.^142^ Additionally, trials assessing continuous outcomes typically report narrower CIs, which in turn may result in less overlap between CIs and higher *I*^2^ values.^142,143^ Considering these limitations and based on the GRADE approach,^144^ we focused on the similarity of the point estimates and the degree of overlap of the CIs. In fact, there was high consistency in effect estimates between trials included in the analyses. For example, of 109 trials included in the analysis of body weight, 104 trials reported a reduction in body weight associated with aerobic exercise. We did not downgrade the certainty of the evidence for inconsistencies given the high consistency in the direction of the effect estimates. Second, due to the low number of studies, we could not perform nonlinear dose-response meta-analyses based on the health status of the participants (eg, in patients with type 2 diabetes). Third, a low number of trials were available for some outcomes, such as health-related quality of life and medication use reduction. Fourth, we used study level data for dose-response meta-analyses; thus, our results are subject to an aggregation bias. Fifth, there was substantial evidence of publication bias for subcutaneous adipose tissue, and trim-and-fill analysis indicated a weaker association. Thus, the magnitude of the findings between aerobic exercise and subcutaneous adipose tissue may have been overestimated. Finally, the trials included in the meta-analysis provided insufficient data regarding the dietary habits and smoking status of the participants. Consequently, we could not account for the influence of these important effect modifiers in our estimates of effects.

## Conclusions

This dose-response meta-analysis of 116 randomized clinical trials presented evidence of moderate to high certainty that aerobic exercise may be associated with clinically important reductions in waist circumference and measures of body fat, including body fat percentage, fat mass, and visceral and subcutaneous adipose tissue areas. The results indicated that levels of body weight, waist circumference, and fat decreased linearly or monotonically in association with increasing duration of aerobic exercise, suggesting that longer durations of aerobic exercise may be associated with more beneficial weight or waist circumference outcomes. By contrast, we did not find any credible subgroup differences based on the intensity of aerobic exercise. Specifically, our results suggest that aerobic training exceeding 150 minutes per week at moderate intensity or greater may be needed to achieve associations with clinically important reductions.

## Notes

Supplement 1. **eMethods 1.** Detailed information on criteria applied for excluding studies **eMethods 2.** Detailed information on systematic search, data extraction and risk of bias assessment **eMethods 3.** Detailed information to evaluate the overall quality of the evidence using GRADE tool **eTable 1.** Search strategy to find potential eligible trials for inclusion in the meta-analysis of supervised aerobic exercise and measures of body weight, waist and fat (April 2024) **eTable 2.** Description of the ICEMAN domains and how to judge each domain **eTable 3.** List of studies that were excluded via full text assessment **eTable 4.** Characteristics of the trials included in the meta-analysis of aerobic exercise and measures of body weight, waist, and fat **eTable 5.** Dropout, degree of adherence to the intervention program, and adverse events in the trials included in the meta-analysis of aerobic exercise and measures of body weight, waist, and fat **eTable 6.** Risk of bias of the trials included in the meta-analysis of aerobic exercise and measures of body weight, waist and fat **eTable 7.** Subgroup analyses of the association of supervised aerobic exercise (each 30 min/week) with body weight (kg) **eTable 8.** Assessment of credibility of subgroup difference based on ICEMAN for body weight **eTable 9.** Subgroup analyses of the association of supervised aerobic exercise (each 30 min/week) with waist circumference (cm) **eTable 10.** Assessment of credibility of subgroup difference based on ICEMAN for waist circumference **eTable 11.** Subgroup analyses of the association of supervised aerobic exercise (each 30 min/week) with body fat percentage (%) **eTable 12.** Assessment of credibility of subgroup difference based on ICEMAN for body fat percentage **eTable 13.** Subgroup analyses of the association of supervised aerobic exercise (each 30 min/week) with body fat mass (kg) **eTable 14.** Assessment of credibility of subgroup difference based on ICEMAN for body fat mass **eTable 15.** Subgroup analyses of the association of supervised aerobic exercise (each 30 min/week) with visceral adipose tissue (cm2) **eTable 16.** Assessment of credibility of subgroup difference based on ICEMAN for visceral adipose tissue **eTable 17.** Subgroup analyses of the association of supervised aerobic exercise (each 30 min/week) with subcutaneous adipose tissue (cm2) **eTable 18.** Assessment of credibility of subgroup difference based on ICEMAN for visceral adipose tissue **eTable 19.** The association of different doses of aerobic exercise with measures of body weight, waist and fat in adults with overweight or obesity (mean difference and 95%CI) **eTable 20.** GRADE evidence for the association of aerobic exercise with measures of body weight, waist and fat **eFigure 1.** Literature search and study selection process **eFigure 2.** Absolute effect of aerobic exercise on adverse events in the study participants **eFigure 3.** Relative effect of aerobic exercise on adverse events in the study participants **eFigure 4.** Absolute effect of aerobic exercise on hypoglycemic reactions in the study participants **eFigure 5.** Relative effect of aerobic exercise on hypoglycemic reactions in the study participants **eFigure 6.** Association of aerobic exercise (30 min/week) with waist circumference (cm) **eFigure 7.** Association of aerobic exercise (30 min/week) with body fat percentage (%) **eFigure 8.** Association of aerobic exercise (30 min/week) with body fat mass (km) **eFigure 9.** Association of aerobic exercise (30 min/week) with visceral adipose tissue (cm2) **eFigure 10.** Association of aerobic exercise (30 min/week) with subcutaneous adipose tissue (cm2) **eFigure 11.** Absolute effect of aerobic exercise on medication reduction **eFigure 12.** Relative effect of aerobic exercise on medication reduction **eFigure 13.** Effect of aerobic exercise on health-related quality of life (mental score) **eFigure 14.** Effect of aerobic exercise on health-related quality of life (physical score) **eFigure 15.** Dose-dependent association of aerobic exercise with visceral adipose tissue (cm2) **eFigure 16.** Dose-dependent association of aerobic exercise with subcutaneous adipose tissue (cm2) **eFigure 17.** Funnel plot of the association of aerobic exercise with body weight (Egger’s test = 0.42) **eFigure 18.** Funnel plot of the association of aerobic exercise with waist circumference (Egger’s test = 0.21) **eFigure 19.** Funnel plot of the association of aerobic exercise with body fat percentage (Egger’s test = 0.003) **eFigure 20.** Funnel plot of the association of aerobic exercise with body fat mass (Egger’s test = 0.55) **eFigure 21.** Funnel plot of the association of aerobic exercise with visceral adipose tissue (Egger’s test = 0.15) **eFigure 22.** Funnel plot of the association of aerobic exercise with subcutaneous adipose tissue (Egger’s test <0.001)

Supplement 2.
Data Sharing Statement
