# Supplementary material for: Aerobic Exercise and Weight Loss in Adults: A Systematic Review and Dose-Response Meta-Analysis
Source: JAMA Netw Open. 2024 Dec 26;7(12):e2452185. doi: 10.1001/jamanetworkopen.2024.52185 (PMC11672165; doi:10.1001/jamanetworkopen.2024.52185)
Supplement: Supplement 1. — eMethods 1. Detailed information on criteria applied for excluding studies eMethods 2. Detailed information on systematic search, data extraction and risk of bias assessment eMethods 3. Detailed information to evaluate the overall quality of the evidence using GRADE tool eTable 1. Search strategy to find potential eligible trials for inclusion in the meta-analysis of supervised aerobic exercise and measures of body weight, waist and fat (April 2024) eTable 2. Description of the ICEMAN domains and how to judge each domain eTable 3. List of studies that were excluded via full text assessment eTable 4. Characteristics of the trials included in the meta-analysis of aerobic exercise and measures of body weight, waist, and fat eTable 5. Dropout, degree of adherence to the intervention program, and adverse events in the trials included in the meta-analysis of aerobic exercise and measures of body weight, waist, and fat eTable 6. Risk of bias of the trials included in the meta-analysis of aerobic exercise and measures of body weight, waist and fat eTable 7. Subgroup analyses of the association of supervised aerobic exercise (each 30 min/week) with body weight (kg) eTable 8. Assessment of credibility of subgroup difference based on ICEMAN for body weight eTable 9. Subgroup analyses of the association of supervised aerobic exercise (each 30 min/week) with waist circumference (cm) eTable 10. Assessment of credibility of subgroup difference based on ICEMAN for waist circumference eTable 11. Subgroup analyses of the association of supervised aerobic exercise (each 30 min/week) with body fat percentage (%) eTable 12. Assessment of credibility of subgroup difference based on ICEMAN for body fat percentage eTable 13. Subgroup analyses of the association of supervised aerobic exercise (each 30 min/week) with body fat mass (kg) eTable 14. Assessment of credibility of subgroup difference based on ICEMAN for body fat mass eTable 15. Subgroup analyses of the association of supervised ae [file jamanetwopen-e2452185-s001.pdf]

Supplementary Online Content

Jayedi A, Soltani S, Emadi A, Zargar MS, Najafi A. Aerobic exercise and weight loss in adults: a systematic review and dose-response meta-analysis. *JAMA Netw Open*. 2024;7(12):e2452185. doi:10.1001/jamanetworkopen.2024.52185

eMethods 1. Detailed information on criteria applied for excluding studies

eMethods 2. Detailed information on systematic search, data extraction and risk of bias assessment

eMethods 3. Detailed information to evaluate the overall quality of the evidence using GRADE tool

eTable 1. Search strategy to find potential eligible trials for inclusion in the meta-analysis of supervised aerobic exercise and measures of body weight, waist and fat (April 2024)

eTable 2. Description of the ICEMAN domains and how to judge each domain

eTable 3. List of studies that were excluded via full text assessment

eTable 4. Characteristics of the trials included in the meta-analysis of aerobic exercise and measures of body weight, waist, and fat

eTable 5. Dropout, degree of adherence to the intervention program, and adverse events in the trials included in the meta-analysis of aerobic exercise and measures of body weight, waist, and fat

eTable 6. Risk of bias of the trials included in the meta-analysis of aerobic exercise and measures of body weight, waist and fat

eTable 7. Subgroup analyses of the association of supervised aerobic exercise (each 30 min/week) with body weight (kg)

eTable 8. Assessment of credibility of subgroup difference based on ICEMAN for body weight

eTable 9. Subgroup analyses of the association of supervised aerobic exercise (each 30 min/week) with waist circumference (cm)

eTable 10. Assessment of credibility of subgroup difference based on ICEMAN for waist circumference

eTable 11. Subgroup analyses of the association of supervised aerobic exercise (each 30 min/week) with body fat percentage (%)

© 2024 Jayedi A et al. *JAMA Network Open*.

eTable 12. Assessment of credibility of subgroup difference based on ICEMAN for body fat percentage

eTable 13. Subgroup analyses of the association of supervised aerobic exercise (each 30 min/week) with body fat mass (kg)

eTable 14. Assessment of credibility of subgroup difference based on ICEMAN for body fat mass

eTable 15. Subgroup analyses of the association of supervised aerobic exercise (each 30 min/week) with visceral adipose tissue (cm2)

eTable 16. Assessment of credibility of subgroup difference based on ICEMAN for visceral adipose tissue

eTable 17. Subgroup analyses of the association of supervised aerobic exercise (each 30 min/week) with subcutaneous adipose tissue (cm2)

eTable 18. Assessment of credibility of subgroup difference based on ICEMAN for visceral adipose tissue

eTable 19. The association of different doses of aerobic exercise with measures of body weight, waist and fat in adults with overweight or obesity (mean difference and 95%CI)

eTable 20. GRADE evidence for the association of aerobic exercise with measures of body weight, waist and fat

eFigure 1. Literature search and study selection process

eFigure 2. Absolute effect of aerobic exercise on adverse events in the study participants

eFigure 3. Relative effect of aerobic exercise on adverse events in the study participants

eFigure 4. Absolute effect of aerobic exercise on hypoglycemic reactions in the study participants

eFigure 5. Relative effect of aerobic exercise on hypoglycemic reactions in the study participants

eFigure 6. Association of aerobic exercise (30 min/week) with waist circumference (cm)

eFigure 7. Association of aerobic exercise (30 min/week) with body fat percentage (%)

eFigure 8. Association of aerobic exercise (30 min/week) with body fat mass (km)

eFigure 9. Association of aerobic exercise (30 min/week) with visceral adipose tissue (cm2)

© 2024 Jayedi A et al. *JAMA Network Open*.

eFigure 10. Association of aerobic exercise (30 min/week) with subcutaneous adipose tissue (cm2)

eFigure 11. Absolute effect of aerobic exercise on medication reduction

eFigure 12. Relative effect of aerobic exercise on medication reduction

eFigure 13. Effect of aerobic exercise on health-related quality of life (mental score)

eFigure 14. Effect of aerobic exercise on health-related quality of life (physical score)

eFigure 15. Dose-dependent association of aerobic exercise with visceral adipose tissue (cm2)

eFigure 16. Dose-dependent association of aerobic exercise with subcutaneous adipose tissue (cm2)

eFigure 17. Funnel plot of the association of aerobic exercise with body weight (Egger's test = 0.42)

eFigure 18. Funnel plot of the association of aerobic exercise with waist circumference (Egger's test = 0.21)

eFigure 19. Funnel plot of the association of aerobic exercise with body fat percentage (Egger's test = 0.003)

eFigure 20. Funnel plot of the association of aerobic exercise with body fat mass (Egger's test = 0.55)

eFigure 21. Funnel plot of the association of aerobic exercise with visceral adipose tissue (Egger's test = 0.15)

eFigure 22. Funnel plot of the association of aerobic exercise with subcutaneous adipose tissue (Egger's test <0.001)

eReferences.

This supplementary material has been provided by the authors to give readers additional information about their work.

eMethod 1. Detailed information on criteria applied for excluding studies

Observational studies, non-randomized trials, trials without sufficient information on training properties (intensity, frequency, and duration), trials with an active control group (e.g., trials that compared the effects of two types of exercise against each other), those with an unsupervised aerobic exercise program as an intervention, and trials conducted in lactating or pregnant women were excluded. In addition, we excluded trials that implemented a high intensity interval training program since this type of aerobic exercise has a different timing regimen compared to continuous aerobic exercise programs and, thus, cannot be pooled with other types in the dose-response meta-analysis. Trials that implemented a co-intervention (e.g., calorie restriction) were eligible if that co-intervention was implemented in both study arms.

eMethod 2. Detailed information on systematic search, data extraction and risk of bias assessment

Based on our search strategy, AJ conducted the literature search and then, teams of two authors (AE/MSZ and SS/MR) independently reviewed the titles and abstracts. The full texts of relevant articles and the reference lists of relevant meta-analyses were then reviewed. There were no restrictions on date or publication status, but for financial reasons we limited the present systematic review to articles published in English. Data extraction was conducted by teams of two reviewers (MSZ/AE and SS/MR), working independently and in duplicate. Disagreements were resolved through consultation with the first author (AJ). We extracted author name, study location, year of publication, intervention duration, population characteristics (% female, mean age, baseline BMI, health status), number of participants, intervention characteristics (frequency, duration, intensity), exercise type (e.g., walking, swimming, cycling) and modality (progressive versus non-progressive), comparison group, co-intervention in both study arms (e.g., calorie restriction), compliance to the intervention, and dropout. We assessed risk of bias of the trials using version 2 of the Cochrane risk of bias tool. [1] Two reviewers (AJ and SS), in duplicate, performed the risk of bias assessments. Disagreements were resolved through consensus.

© 2024 Jayedi A et al. *JAMA Network Open*.

eMethod 3. Detailed information to evaluate the overall quality of the evidence using GRADE tool

According to the GRADE approach, randomized controlled trials yield high certainty initial evidence that can be subsequently upgraded or downgraded in accordance with predefined criteria. Criteria used to upgrade the certainty of evidence include large effect size and dose-response gradient. Serious risk of bias, inconsistency, indications of publication bias, imprecision, and indirectness are among the criteria for downgrading evidence. We adapted a recently published GRADE minimally contextualized approach to rate imprecision based on minimally important difference. [2,3] Accordingly, we considered whether the point estimate of effect size was greater than or less than the minimally important difference, and whether the 95%CI overlapped with that threshold. The minimally important difference thresholds were as follows: 4.5 kg for body weight, 2 cm for waist circumference, 2% for body fat percentage, 2 kg for body fat mass, and 5 cm^2 for visceral and subcutaneous adipose tissue. [4,5] We considered 2% change in absolute effect as important difference for binary outcomes. [6] To assess the presence of inconsistency, we did not rely solely on I^2 value, as this can be misleading when evaluating continuous outcomes, which generally have narrower Cis. [7,8] Instead, the similarity of the point estimates and the degree of overlap of the CIs were the two criteria used to assess inconsistency. [9] We assessed publication bias by examining the impact of small studies using funnel plots as suggested by the GRADE approach. [10]

© 2024 Jayedi A et al. *JAMA Network Open*.

eTable 1. Search strategy to find potential eligible trials for inclusion in the meta-analysis of supervised aerobic exercise and measures of body weight, waist and fat (April 2024)

PubMed (11,624)
1. "Exercis*"[Title/Abstract] OR "aerobic training*"[Title/Abstract] OR "running"[Title/Abstract] OR "swim*"[Title/Abstract] OR "aerobic exercis*"[Title/Abstract] OR "endurance train*"[Title/Abstract] OR "high intensity interval train*"[Title/Abstract] OR "HIIT"[Title/Abstract] OR "moderate intensity interval train*"[Title/Abstract] OR "moderate intensity continuous train*"[Title/Abstract] OR "MICT"[Title/Abstract] OR "low intensity train*"[Title/Abstract] OR "anaerobic train*"[Title/Abstract] OR "physical activit*"[Title/Abstract] OR "Treadmill"[Title/Abstract] OR "cycling"[Title/Abstract] OR "Swimming"[Title/Abstract] OR "physical exercise"[Title/Abstract] OR "exercise therapy"[Title/Abstract] OR "Exercise"[MeSH Terms]
2. "Body Weight"[MeSH Terms] OR "Weight Loss"[MeSH Terms] OR "waist circumference*"[All Fields] OR "adipose tissue*"[All Fields] OR "body fat"[All Fields] OR "body mass"[All Fields] OR "Body Weight"[All Fields] OR "Fat mass"[All Fields] OR "Fat free mass"[All Fields] OR "adipose"[All Fields] OR "adiposities"[All Fields] OR "adiposity"[MeSH Terms] OR "adiposity"[All Fields] OR "obesity"[MeSH Terms] OR "obesity"[All Fields] OR "obese"[All Fields] OR "obesities"[All Fields] OR "obesity "[All Fields] OR "Intra-abdominal fat"[All Fields] OR "abdominal fat"[All Fields] OR "Weight Loss"[All Fields] OR "subcutaneous fat"[All Fields] OR "subcutaneous abdominal fat"[All Fields] OR "visceral adiposit*"[All Fields] OR "visceral fat*"[All Fields] OR "central adiposit*"[All Fields] OR "body mass index"[All Fields] OR "BMI"[All Fields]
3. "Overweight"[MeSH Terms] OR "Overweight"[All Fields] OR "overweighted"[All Fields] OR "overweightness"[All Fields] OR "overweights"[All Fields] OR "obeses"[All Fields] OR "Obesity"[MeSH Terms] OR "Obesity"[All Fields] OR "obese"[All Fields] OR "obesities"[All Fields] OR "obesity s"[All Fields] OR "abdominal obesity"[All Fields] OR "morbid obesity"[All Fields] OR "obese"[All Fields] OR "central obesity"[All Fields] OR "Overweight"[MeSH Terms] OR "Obesity"[MeSH Terms] OR "obesity, abdominal"[MeSH Terms] OR "obesity, morbid"[MeSH Terms] OR "Obesity Management"[MeSH Terms]
4. "Randomized"[Title/Abstract] OR "Randomised"[Title/Abstract] OR "placebo"[Title/Abstract] OR "randomly"[Title/Abstract] OR "trial"[Title] OR "Controlled Clinical Trial"[Publication Type] OR "Clinical Trials as Topic"[MeSH Terms] OR "Randomized Controlled Trial"[Publication Type]
5. #1 AND #2
6. #3 AND #4
7. #5 AND #6
Scopus (15,455)
CENTRAL (2536)
Clinicaltrial.gov (453)
ProQuest (245)
Total (30,493)

© 2024 Jayedi A et al. *JAMA Network Open*.

eTable 2. Description of the ICEMAN domains and how to judge each domain

**1: Is the analysis of effect modification based on comparison within rather than between trials?**			
Completely between	Mostly between or unclear	Mostly within	Completely within
Subgroup analysis or meta-regression comparing overall effects of each individual trial. This is typical for aggregate data meta-analysis.	Subgroup analysis or meta-regression with most information coming from overall effects, but some trials providing within-trial subgroup information	Most trials providing within-trial subgroup information; or individual participant data analysis that combines within and between trial information	All trials providing within-trial subgroup information or individual participant data; and the analysis separates within from between trial information, e.g. meta-analysis of interactions
**2: For within-trial comparisons, is the effect modification similar from trial to trial?** Not applicable: no or one within-RCT comparison			
Definitely not similar	Probably not similar or unclear	Mostly similar	Definitely similar
Effect modification reported for two or more trials and clearly different directions	Effect modification not reported for individual trials or too imprecise to tell	Effect modification reported for two or more trials, mostly similar in direction, but considerable differences in magnitude	Effect modification reported for two or more trials, similar in direction, only some differences in magnitude
**3: For between-trial comparisons, is the number of trials large?** [ ] Not applicable: no between RCT comparison			
Very small	Rather small or unclear	Rather large	Large
1 or 2 or in smallest subgroup; 5 or less in continuous meta-regression	3-4 in smallest subgroup; 6-10 in continuous meta-regression	5-9 in smallest subgroup; 11 to 15in continuous meta-regression	10 or more in smallest subgroup; more than 15 in continuous meta-regression
**4: Was the direction of effect modification correctly hypothesized a priori?**			
Definitely no	Probably no or unclear	Probably yes	Definitely yes
Clearly post-hoc or results inconsistent with hypothesized direction or biologically very implausible	Vague hypothesis or hypothesized direction unclear	No prior protocol available but unequivocal statement of a priori hypothesis with correct direction of effect modification	Prior protocol available and includes correct specification of direction of effect modification, e.g. based on a biologic rationale
**5: Does a test for interaction suggest that chance is an unlikely explanation of the apparent effect modification?** (consider irrespective of number of effect modifiers)			
Chance a very likely explanation	Chance a likely explanation or unclear	Chance may not explain	Chance an unlikely explanation
Interaction or meta-regression p-value >0.05	Interaction or meta-regression p-value ≤0.05 and >0.01, or no test of interaction reported and not computable	Interaction or meta-regression p-value ≤0.01 and >0.005	Interaction or meta-regression p-value ≤0.005
**6: Did the authors test only a small number of effect modifiers or consider the number in their statistical analysis?**			
Definitely no	Probably no or unclear	Probably yes	Definitely yes
Explicitly exploratory analysis or large number of effect modifiers tested (e.g. greater than 10) and multiplicity not considered in analysis	No mention of number or 4-10 effect modifiers tested and number not considered in analysis	No protocol available but unequivocal statement of 3 or fewer effect modifiers tested	Protocol available and 3 or fewer effect modifiers tested or number considered in analysis
**7: Did the authors use a random effects model?**			

© 2024 Jayedi A et al. *JAMA Network Open*.

Definitely no	Probably no or unclear	Probably yes	Definitely yes
Fixed (or common) effect or fixed effects model explicitly stated	Probably fixed effect(s) model	Probably random (or mixed) effects	Random (or mixed) effects explicitly stated
**8: If the effect modifier is a continuous variable, were arbitrary cut points avoided?** [ ] not applicable: not continuous			
Definitely no	Probably no or unclear	Probably yes	Definitely yes
Analysis based on exploratory cut point(s), e.g. picking cut point associated with highest interaction p-value	Analysis based on cut point(s) of unclear origin	Analysis based on pre-specified cut point(s), e.g. suggested by prior RCT	Analysis based on the full continuum, e.g. assuming a linear or logarithmic relationship
**9 Optional: Are there any additional considerations that may increase or decrease credibility?** (manual section 3.9) [ ] not applicable			
yes, probably decrease		yes, probably increase	
**10. How would you rate the overall credibility of the proposed effect modification?**<br>Overall rating: The overall rating should be derived by the items that decrease credibility:<br>**Very low**: All responses definitely or probably decrease credibility or unclear<br>**Maximum usually low**: Two or more responses decrease credibility even if all other responses satisfy credibility criteria<br>**Maximum usually moderate**: One response definitely decreases credibility even if all other responses satisfy credibility criteria<br>**Maximum usually moderate**: Two responses probably decrease credibility even if all other responses satisfy credibility criteria<br>**High very likely**: No response options definitely or probably decrease credibility			
**11. How would you interpret the overall credibility of the proposed effect modification?**<br>**Very low**: Very likely no effect modification. Use overall effect for each subgroup.<br>**Maximum usually low**: Likely no effect modification. Use overall effect for each subgroup but note remaining uncertainty.<br>**Maximum usually moderate**: Likely effect modification. Use separate effect for each subgroup but note remaining uncertainty.<br>**High very likely**: Very likely effect modification. Use separate effect for each subgroup.			

© 2024 Jayedi A et al. *JAMA Network Open*.

eTable 3. List of studies that were excluded via full text assessment

**Excluded (n=235)**
1. Not in patients with overweight or obesity (n=58) [11-68]
2. Not sufficient information (n=30) [69-98]
3. Not relevant intervention (n=30) [99-128]
4. Trials with an active control (n=28) [129-156]
5. Non-supervised aerobic exercise (n=16) [157-172]
6. Duplicate publications (n=13) [173-185]
7. Quasi experimental (n=15) [186-200]
8. High intensity interval training (n=12) [201-212]
9. Multicomponent intervention (n=11) [213-223]
10. Shorter than 8 weeks (n=10) [224-233]
11. Not in English (n=4) [234-237]
12. Follow-up studies (n=3) [238-240]
13. Before after studies (n=2) [241,242]
14. In children (n=2) [243,244]
15. In lactating women (n=1) [245]

© 2024 Jayedi A et al. *JAMA Network Open*.

eTable 4. Characteristics of the trials included in the meta-analysis of aerobic exercise and measures of body weight, waist, and fat

Author[a], year, Country	Participants	Sample size (%female)	Age range (mean/ median)	Mean BMI (kg/m^2)	Exercise program	Intervention duration (weeks)	Co-intervention	Outcome(s)
Abdelaal et al, [26] 2014 Egypt	Patients with obesity, diabetes, and mild essential hypertension	39 (56%)	45-60 (52.5)	34	Int: Supervised moderate intensity exercise program on treadmill (3/week; each 20-30 min on 60—65% of HRmax intensity during the first month, 35—40 min on 65—70% of HRmax intensity during the second month, and 40—50 min on 70—75% of HRmax intensity during the third month)<br><br>Con: Without participation in any supervised regular training	12	None	Waist circumference
Ahmadi et al, [27] 2021 Iran	Men with type 2 diabetes and overweight or obesity	24 (0)	(48.7)	30.7	Int: Supervised running, five sessions per week, for one hour with an intensity of 50 to 75% of the HRR<br><br>Con: No intervention	8	None	Body weight, waist circumference, body fat percentage
Ahmadizad et al, [28] 2007 Iran	Healthy non-smoker men with obesity	16 (0%)	35-48 (40)	29	Int: Supervised running at 75–85% of maximal heart rate for 20–30 min per day (progressive), 3/week<br><br>Con: No intervention	12	None	Body weight, body fat percentage
Akbarpoor et al, [29] 2013 Iran	Non-athlete men with obesity	60 (0%)	20-25 (23)	31	Int: Supervised aerobic training (3/week) included a 10-min warm-up, then, continuous running with intensity between 75 and 85% of the maximum heart rate. The running period was 15 min for the first session, and every two sessions 1.5 min was added to the running period in a stepwise manner until the running period reached 30 min<br><br>Con: No intervention	12	None	Body fat percentage

© 2024 Jayedi A et al. *JAMA Network Open*.

Author[a], year, Country	Participants	Sample size (%female)	Age range (mean/ median)	Mean BMI (kg/m^2)	Exercise program	Intervention duration (weeks)	Co-intervention	Outcome(s)
Alvez et al, [30] 2009 Brazil	Sedentary women with overweight or obesity	145 (100%)	20-60 (38.2)	30	Int: Supervised exercise consisting of three 50-minute moderate intensity aerobic sessions each week on 40% to 60% of heart rate reserve<br><br>Con: No intervention	24	None	Body weight
Amanat et al, [31] 2020 Iran	Women with overweight and metabolic syndrome	29 (100%)	(54.5)	29	Int: Supervised aerobic exercises (3/week; each 60 min/day), on 60% HRmax that was gradually elevated to 75%<br><br>Con: No change in physical activity during the study	12	None	Body weight, body fat percentage
Aminlari et al, [32] 2017 Iran	Middle-aged women with overweight and type 2 diabetes	27 (100%)	45-60	29	Int: Supervised progressive aerobic exercise (3/week), consisted of three phases of warm up (20 min), the main stage (25 min) and a cool-down period (15 min) on 50% to 55% of maximum heart rate<br><br>Con: No intervention	12	None	Body weight, body fat percentage
Anderssen et al, [33] 2007 Norway	Middle-aged males with the metabolic syndrome and overweight or obesity	60 (0%)	40-49 (44.9)	29	Int: Supervised endurance-based exercise, such as aerobics, circuit training, and fast walking/jogging (3/week; each 60 min) on 60–80% of the participant's individual peak heart rate<br><br>Con: No intervention	52	None	Body weight, waist circumference
Armannia et al, [34] 2022 Iran	Middle age with obesity	24 (50%)	(45)	39	Int: Supervised MICT, 40 min of training per session (three times/week) with an intensity of 40- 75% HR max<br><br>Con: No intervention	8	None	Body weight, Body fat percentage
Arsenault et al, [35]	Healthy postmenopausal	349 (100%)	45-75 (57)	32	Int: Supervised aerobic exercise 3 to 4 times per week, cycling and treadmill,	24	None	Body weight, waist

© 2024 Jayedi A et al. *JAMA Network Open*.

Author[a], year, Country	Participants	Sample size (%female)	Age range (mean/ median)	Mean BMI (kg/m^2)	Exercise program	Intervention duration (weeks)	Co-intervention	Outcome(s)
2009<br>US	women with overweight or obesity				at a targeted heart rate corresponding to 50% of the maximal oxygen consumption<br><br>Con: No intervention			circumference, body fat percentage
Arslan et al, [36]<br>2017<br>Turkey	Middle-aged premenopausal sedentary women with overweight or obesity	44<br>(100%)	35-45<br>(39)	29	Int: Supervised aerobic exercise with 1-h main exercise sessions 3 times per week at 60—70% of maximum heart rate during 1—4 weeks and at 60—70% of HRmax<br><br>Con: No intervention	8	None	Body weight, waist circumference, fat mass, body fat percentage
Auerbach et al, [37]<br>2013<br>Denmark	Caucasian young men with moderate overweight	24<br>(0%)	20-40	28	Int: Supervised endurance training (jogging, cycling, rowing, cross training) prescribed 7 days per week equivalent to an increase in daily energy expenditure of 600 kcal. 3-4 days per week the training was intense (~85% of their maximal heart rate reserve) and for the remaining sessions the intensity was moderate (~65% of heart rate reserve)<br><br>Con: Continue a sedentary lifestyle	12	None	Body weight, body fat percentage
Baria et al, [38]<br>2014<br>Brazil	Sedentary men with overweight and chronic kidney disease	19<br>(0%)	(52)	30	Int: Supervised progressive aerobic training on a treadmill (3/week; each 30-60 min) on 40-60% Vo2 peak<br><br>Con: No intervention	12	None	Body weight, waist circumference, fat mass, visceral fat, subcutaneous fat
Beavers et al, [39]<br>2017<br>US	Community dwelling older adults with	166<br>(67%)	60-79<br>(67)	34	Int: 30 minutes of moderately intense supervised activity on most days of the	18	Calorie restriction for a total loss in	Body weight, body fat

© 2024 Jayedi A et al. *JAMA Network Open*.

Author[a], year, Country	Participants	Sample size (%female)	Age range (mean/ median)	Mean BMI (kg/m^2)	Exercise program	Intervention duration (weeks)	Co-intervention	Outcome(s)
	overweight at risk of cardiovascular disease				week for a total of more than 150 minutes/week<br><br>Con: Participants were taught how to actively "take charge" of their health		mass of 7% to 10%	percentage, fat mass
Bell et al, [40] 2010 Canada	Sedentary men and women with overweight or obesity	88 (NR)	25-65 (50)	30	Int: Pedo-meter based progressive daily walking monitored with pedometers (7/weeks; each 30-60 min/week) at 70% VO2 peak<br><br>Con: Asked to maintain their usual activity	24	None	Body weight, waist circumference
Belli et al, [41] 2011 Brazil	Sedentary women with type 2 diabetes and overweight or obesity	19 (100%)	(55)	31	Int: Supervised walking training program (3/week; each 20-60 min; average: 52 min) at 70-80% HRmax<br><br>Con: No intervention	12	None	Body weight, body fat percentage, fat mass, waist circumference
Benito et al, [42] 2015 Spain	Healthy sedentary participants with obesity	48 (50%)	18-50 (39)	33	Int: Progressive supervised aerobic exercise (running or cycling), 200-300 min/week at 50-60% HRmax<br><br>Con: Physical activity recommendations	22	A hypocaloric diet to provide 30% less energy intake	Body weight, waist circumference, body fat percentage,
Bertram et al, [43] 1990 Canada	Women with obesity	21 (100%)	(37)	34	Int: Supervised progressive aerobic exercise (3/week, each 60 min) at 70% HRmax<br><br>Con: No activity	16	A hypocaloric diet with 1200 kcal/day	Body weight, body fat percentage
Blond et al, [44] 2018 Denmark	Physically inactive, otherwise healthy	95 (53%)	20-45 (34)	30	Int: Progressive supervised aerobic exercise with moderate (50% VO2 peak) and vigorous (70% VO2 peak)	24	None	Body weight, waist circumference, fat mass

© 2024 Jayedi A et al. *JAMA Network Open*.

Author[a], year, Country	Participants	Sample size (%female)	Age range (mean/ median)	Mean BMI (kg/m^2)	Exercise program	Intervention duration (weeks)	Co-intervention	Outcome(s)
	participants with overweight or class 1 obesity				intensity (2-5/week; average:4.6, each 45 min/day)<br><br>Con: No intervention			
Blumenthal et al, [45] 2000 US	Sedentary men and women with hypertension and overweight or obesity	78 (56%)	>29 (48)	33	Int: Supervised progressive aerobic exercise program (3-4/week, each 60 min/day) at 70-85 HRmax<br><br>Con: Waitlist control group that were asked to maintain their usual activity and dietary intake	24	None	Body weight, body fat percentage
Chin et al, [46] 2019 China	Young adults with obesity	23 (100%)	18-30 (22.8)	26.4	Int: Supervised MICT, each session consisted of 30 minutes of continuous exercise at an intensity of 60% of heart rate reserve, 3/week<br><br>Con: No intervention	8	None	Body weight, waist circumference, body fat percentage and mass
Chiu et al, [47] 2017 Taiwan	Sedentary adults with obesity	48 (28%)	18-26	≥ 27	Int: Light supervised aerobic exercise for 40%–50% heart rate reserve during weeks 1-12<br><br>Con: No intervention	12	None	Body weight, waist circumference, body fat percentage and mass
Cho et al, [48] 2011 Korea	Sedentary adults with obesity	35 (100%)	34-60 (45.4)	≥ 25	Int: LIEX: supervised moderate-intensity walking exercise progressed to 3 sessions (days) per week by week 5 at an intensity of 40– 50 % of VO2max or HIEX: supervised vigorous-intensity walking / jogging exercise progressed to 3 sessions	12	None	Body weight, waist circumference, visceral abdominal adipose tissue, subcutaneous

© 2024 Jayedi A et al. *JAMA Network Open*.

Author[a], year, Country	Participants	Sample size (%female)	Age range (mean/ median)	Mean BMI (kg/m^2)	Exercise program	Intervention duration (weeks)	Co-intervention	Outcome(s)
					(days) per week by week 5 at an intensity of 70 – 75 % of VO2max<br><br>Con: Maintained their current sedentary lifestyle			abdominal adipose tissue
Chow et al, [49] 2020 China	Young women with obesity	31 (100%)	≥18	24.5	Int: Supervised aerobic exercise including descending or ascending stairs (low or moderate intensity (about 54–73% HRmax), duration regime lasted 8 minutes in the 1st week and increased to 43 min and 12s in the final week<br><br>Con: No training	12	None	Body weight, Body fat percentage and mass
Christiansen et al, [50] 2009 Denmark	Healthy adults with obesity	40 (50%)	18-45 (36.2)	34.3	Int: Supervised aerobic exercise three times per week with a duration of 60–75 min per training session at 70% of HRmax<br><br>Con: No intervention	12	Very low energy diet (600 kcal/day)	Body weight, waist circumference, Body fat percentage, visceral and subcutaneous fat
Coker et al, [51] 2009 US	Elderly adults with overweight	18 (50%)	65-90 (71.1)	30	Int: Supervised moderate-intensity (50% of VO2peak) exercise training or supervised high-intensity (75% of VO2peak) exercise training (4-5/week, each session ~30 min)<br><br>Con: No intervention	12	None	Abdominal visceral fat
Cooper et al, [52] 2016 Australia	Middle-aged men with obesity	29 (0%)	40-60 (51.1)	29.3	Int: Supervised endurance training consisted of cycle ergometry at workplace 3/week, each session ~50–60 min) at 80% HRmax	12	None	Body weight, Body fat percentage and mass, subcutaneous

© 2024 Jayedi A et al. *JAMA Network Open*.

Author[a], year, Country	Participants	Sample size (%female)	Age range (mean/ median)	Mean BMI (kg/m^2)	Exercise program	Intervention duration (weeks)	Co-intervention	Outcome(s)
					Con: No intervention			adipose tissue, abdominal visceral adipose tissue
Cornish et al, [53] 2021 Canada	Adults with Knee osteoarthritis and overweight	16 (56%)	45-65 (57.2)	31.4	Int: Supervised supported low-load walking on an Alter-G treadmill (60- 70 HRmax) each session ~30 min 3/ week<br><br>Con: Maintenance normal activity	12	None	Body weight
Cuff et al, [54] 2023 Canada	Postmenopausal women with type 2 diabetes and obesity	18 (100%)	60	32.8	Int: Supervised aerobic training (75-min 3/week) at 60–75% HRmax<br><br>Con: No intervention	16	None	Body weight, total abdominal obesity, subcutaneous adipose tissue, visceral adipose tissue
Dash et al, [55] 2018 US	Postmenopausal metabolically unhealthy women with obesity	144 (100%)	45-65 (58.3)	35.4	Int: Supervised facility-based aerobic exercise intervention with a goal of 150 min/week of moderate intensity exercise at 45–65% VO2max (each session ~50 min 3/ week)<br><br>Con: Maintain baseline daily activities	24	None	Waist circumference
Davidson et al, [56] 2009 Canada	Sedentary older adults with abdominal obesity	54 (68%)	60-80	30.1	Int: Supervised moderate-intensity treadmill walking with a goal of 150 min/week at 60%-75% VO2max (each session ~50 min 3/ week)	24	None	Body weight, waist circumference, Body fat percentage and mass, abdominal subcutaneous fat, visceral fat

© 2024 Jayedi A et al. *JAMA Network Open*.

Author[a], year, Country	Participants	Sample size (%female)	Age range (mean/ median)	Mean BMI (kg/m^2)	Exercise program	Intervention duration (weeks)	Co-intervention	Outcome(s)
					Con: No intervention			
Dengel et al, [57] 1996 US	Sedentary men with obesity	19 (0%)	>45	NR	Int: Supervised aerobic exercise including stationary cycling, walking and jogging on a treadmill at 75-80 HR max (each session ~45 min 3/ week)<br><br>Con: A healthy diet	40	None	Body weight, fat mass, body fat percentage
Donges et al, [58] 2013 Australia	Middle-aged men with overweight	21 (0%)	40-65	31.3	Int: Supervised insurance training at 40 min for weeks 1–4, and increased to 50-min and 60-min per session for weeks 5–8 and 9–12, respectively at 75-80 HRmax<br><br>Con: No intervention	12	None	Body weight, Body fat percentage and mass, subcutaneous fat, abdominal visceral fat
Donnelly et al, [59] 2003 US	Young adults with obesity	74 (58%)	17-35 (22.8)	29.1	Int: supervised aerobic exercise including walking on treadmill. Exercise duration progressed from 20 minutes at baseline to 45 minutes at 6 months, and the intensity of exercise progressed from 60% of the heart rate reserve at baseline to 75% at 6 months, 5/ week<br><br>Con: Maintain physical activity and dietary intake patterns	64	None	Body weight, fat mass, body fat percentage, visceral fat, subcutaneous fat
Eizadi et al, [60] 2013 Iran	Middle-aged men with obesity	34 (0%)	43	32.1	Int: Supervised aerobic exercise program consisted of 45 minutes of aerobic training consist of running on treadmill or stationary cycling at 60—80% HRmax, 3/week<br><br>Con: No intervention	12	None	Body weight, waist circumference, Body fat percentage

© 2024 Jayedi A et al. *JAMA Network Open*.

**Author[a], year, Country**	**Participants**	**Sample size (%female)**	**Age range (mean/ median)**	**Mean BMI (kg/m^2)**	**Exercise program**	**Intervention duration (weeks)**	**Co-intervention**	**Outcome(s)**
Elsayed et al, [61]<br>2022<br>Egypt	Postmenopausal women with obesity	60<br>(100%)	60-75	35	Int: Supervised aerobic exercise including walked on the treadmill for 40 minutes until reaching the target intensity 60% of HRmax for the first 6 weeks, and then it was increased to 70% HRmax for the last six weeks, 3/week<br><br>Con: No intervention	12	MIND diet by reducing 20% of the total daily energy expenditure	Body weight
Ezpeletka et al, [62]<br>2023<br>US	Adults with non-alcoholic fatty liver disease and obesity	40<br>(81%)	(44)	37	Int: Supervised moderate-intensity aerobic exercise program, 5/week; Training intensity gradually increased over the first four weeks of the study from 65 to 80% of their maximum predicted heart rate, 60 min per session<br><br>Con: Participants were instructed to maintain their body weight throughout the trial, and not to change their eating or physical activity habits	12	None	Body weight, waist circumference, fat mass, body fat percentage
Fenkci et al, [63]<br>2006<br>Turkey	Adults' women with severe eating disorders and obesity	40<br>(100%)	>18	34.2	Int: supervised aerobic exercise including walk briskly for 15 min and then to exercise on a stationary leg cycle ergometer at 50%- 85% of HRmax, 1st month—3 d a week for 12 to 15 min; 2nd month —4 d a week for 20 to 30 min; 3rd month —5 d a week for 30 to 45 min<br><br>Con: No intervention	12	None	Body weight, waist circumference, body fat percentage
Fisher et al, [64]<br>2011<br>US	Healthy premenopausal women with obesity	72<br>(100%)	20–41	29	Int: Supervised aerobic training included 3/ week of walking/ running on a treadmill, starting at 65% of HRmax for 20 min and gradually	8	Followed a calorie-restricted diet Subjects were	Body weight, Body fat, subcutaneous adipose tissue

© 2024 Jayedi A et al. *JAMA Network Open*.

Author[a], year, Country	Participants	Sample size (%female)	Age range (mean/ median)	Mean BMI (kg/m^2)	Exercise program	Intervention duration (weeks)	Co-intervention	Outcome(s)
					increased until subjects exercised at 80% of HRmax for 40 min at week 8<br><br>Con: No intervention		provided a 800 kcal/day	
Fogelholm et al, [65]<br>2000<br>Finland	Premenopausal women with obesity	74<br>(100%)	40	34	Int: Supervised walking 2 to 3 hours weekly, or supervised walking 4 to 6 hours at 50% to 60% HRmax<br><br>Con: Counseling on diet and relapse prevention	40	None	Body weight, waist circumference, body fat mass
Foster-Schubert et al, [66]<br>2012<br>US	Post-menopausal women with overweight and obesity	204 (100%)	50–75<br>(58)	30.9	Int: Supervised moderate-to-vigorous intensity aerobic training, each session ≥45 minutes 5/week (225 minutes/week), a 15-minute session at 60–70% HRmax and progressed to the target 70–85% HRmax for the 7th week<br><br>Con: Maintain physical activity and dietary intake patterns	48	None	Body weight, waist circumference, body fat percentage
Fu et al, [67]<br>2022<br>US	Nondiabetic adults with obesity	121<br>(88%)	(47.8)	≥35	Int: Supervised moderate-intensity aerobic program to such as brisk walking that progressed to 60 minutes for 5/week<br><br>Con: No intervention	24	A reduced calorie diet from 1200 to 2100 kcal/day based on their initial body weight	Body weight, waist circumference, body fat mass, abdominal subcutaneous adipose tissue, abdominal visceral adipose tissue
Geliebter et al, [68]<br>1997	Premenopausal women with obesity	45<br>(62%)	19-48<br>(36)	NR	Int: Supervised exercise including a stationary leg cycle ergometer for ~30 minutes at 70% HRmax for 3/week	8	Received a liquid- formula that provided ~	Body weight, fat mass

© 2024 Jayedi A et al. *JAMA Network Open*.

Author[a], year, Country	Participants	Sample size (%female)	Age range (mean/ median)	Mean BMI (kg/m^2)	Exercise program	Intervention duration (weeks)	Co-intervention	Outcome(s)
US					Con: No intervention		50% energy intake	
Giannopoulou et al, [69] 2005 US	Postmenopausal women with type 2 diabetes with obesity	22 (100%)	50-70	34.7	Int: Supervised exercise program including walking program 3 to 4/ week for 60 minutes at 65% to 70% Vo2 peak<br><br>Con: No intervention	14	Followed a ~1460-kJ deficit from the diet	Body weight, fat mass, total abdominal tissue, visceral adipose tissue, subcutaneous adipose tissue
Goodpaster et al, [70] 2010 US	Nondiabetic adults with severe obesity	130 (77%)	30-55 (45.3)	43.5	Int: Supervised moderate-intensity physical activity, similar in intensity to brisk walking, 60 minutes, 5/week<br><br>Con: Identical dietary intervention	48	Behavioral lifestyle program, energy intake was reduced to 1200 to 2100 kcal/d	Body weight, waist circumference, abdominal subcutaneous fat, visceral fat
Gram et al, [71] 2017 Denmark	Healthy adults with obesity	71 (60%)	20- 45 (34)	30	Int: Supervised moderate intensity leisure time endurance exercise or vigorous intensity leisure time endurance exercise<br><br>Con: No intervention	24	Decrease in energy expenditure by 25% per session	Body weight
Gram et al, [72] 2010 Denmark	Adults with type 2 diabetes with obesity	44 (49%)	25-80	>25	Int: Supervised walking at a speed of at least moderate intensity (>40% of VO2max) for a minimum of 30 minutes<br><br>Con: No supervised training but got the diabetes outpatient clinic's standard information	16	None	Body weight, waist circumference, Fat tissue mass

© 2024 Jayedi A et al. *JAMA Network Open*.

Author[a], year, Country	Participants	Sample size (%female)	Age range (mean/ median)	Mean BMI (kg/m^2)	Exercise program	Intervention duration (weeks)	Co-intervention	Outcome(s)
Gulsin et al, [73] 2020 UK	Working-age adults with type 2 diabetes and obesity	52 (NR)	18- 65 (51)	36.6	Int: Supervised moderate intensity aerobic exercise titrated to ~60% baseline peak VO2 and heart rate, each session 50 min 3/week<br><br>Con: Standard lifestyle advice	12	None	Body weight
Guzel et al, [74] 2022 Turkey	Sedentary postmenopausal women with obesity	24 (100)	(55)	32.9	Int: Supervised walking/jogging exercise on a treadmill, with training volume increased from 25 min/day (75 min a week) at 50% heart rate reserve (HRreserve) in the 1st week to 40 min/day at 70% HRreserve (120 min a week) in the 10th week<br><br>Con: No intervention	10	None	Body weight, fat mass, body fat percentage, waist circumference
Hara et al, [75] 2005, Japan	Young male with obesity	14 (0%)	(19.2)	31.1	Int: Supervised aerobic training including treadmills and cycle ergometers at ~40.8%-54.8% VO2 max, each session more than 30 min 3/week<br><br>Con: No intervention	8	None	Body weight, body fat mass, body fat percentage
Hays et al, [76] 2006 US	Older adults with impaired glucose tolerance with overweight or obese	22 (59%)	55–80 (66)	31.9	Int: Supervised aerobic training including cycle ergometer, each session 45 min at 80% VO2peak 4 /week<br><br>Con: Maintain physical activity	12	A low-fat/high-carbohydrate diet to provide 150% of predicted energy requirements	Body weight, body fat percentage, abdominal subcutaneous fat, abdominal visceral fat
Herzig et al, [77] 2014 Finland	Adults with prediabetes and obesity	68 (73%)	(58.8)	31.4	Int: Supervised moderate walking, each session 60-min walk at a speed of ~3–4 km h $^{-1}$, 3/ week	12	None	Body weight, body fat percentage, waist

© 2024 Jayedi A et al. *JAMA Network Open*.

Author[a], year, Country	Participants	Sample size (%female)	Age range (mean/ median)	Mean BMI (kg/m^2)	Exercise program	Intervention duration (weeks)	Co-intervention	Outcome(s)
					Con: No intervention			circumference, visceral fat area
Ho et al, [78] 2014 Australia	Adults with overweight and obesity	31 (87%)	40-66 (54)	32.5	Int: Supervised aerobic exercise on a treadmill at 60% heart rate reserve, each session 30-min 5/week<br>Con: No intervention	12	None	Body weight, body fat percentage, waist circumference, body fat mass
Hong et al, [79] 2014 Korea	Women with obesity	20 (100%)	30-40	27.4	Int: Supervised walking at 50-60% VO2max, each session 50-70-min, 3/week<br>Con: Maintained their sedentary lifestyle	12	None	Body weight, body fat percentage, waist circumference, subcutaneous abdominal fat, visceral abdominal fat
Irwin et al, [80] 2003 US	Sedentary postmenopausal women with obesity	173 (100%)	50- 75 (60.1)	30.5	Int: Supervised exercise training at least 45 minutes of moderate-intensity exercise 5 d/wk<br>Con: Attended weekly 45-minute stretching sessions and maintain usual physical activity and dietary intake patterns	48	None	Body weight, body fat percentage, body fat mass, waist circumference, intra-abdominal fat, subcutaneous abdominal fat

© 2024 Jayedi A et al. *JAMA Network Open*.

Author[a], year, Country	Participants	Sample size (%female)	Age range (mean/ median)	Mean BMI (kg/m^2)	Exercise program	Intervention duration (weeks)	Co-intervention	Outcome(s)
Jang et al, [81] 2019 Korea	Middle-aged women with obesity	16 (100%)	50-61 (55.9)	25.6	Int: Supervised aerobic training including Treadmill running at 60–75% HRR for 50 min 4/week<br><br>Con: No intervention	8	None	Body weight, body fat percentage, waist circumference
Janssen et al, [82] 1999 Canada	Adults with upper body obesity	40 (50%)	>18 (41.2)	33.4	Int: Supervised aerobic exercise consists of 15 min at the beginning and progressed to a maximum of 60 min at 50% to 85% of the HRmax, 5/week<br><br>Con: No intervention	16	Calorie-restricted diet by 1000 kcal/day	Body weight, Waist circumference, Body fat mass, subcutaneous abdominal fat, subcutaneous fat, visceral adipose tissue
Jung et al, [83] 2012 Korea	Women with type 2 diabetes mellitus and obesity	28 (100%)	45-65 (53.8)	26.6	Int: Supervised moderate intensity exercise group 5/week, for 60 minutes per session, and vigorous intensity exercise group 5/ week, for 30 minutes per session<br><br>Con: Received education regarding walking exercises, according to ADA	12	None	Body weight, Waist circumference
Jung et al, [84] 2014 Korea	Women with type 2 diabetes mellitus and obesity	35 (100%)	45- 65 (56.4)	26.6	Int: Supervised walking exercise 3/week for 60 min/day at an exercise intensity of higher than 4 according to an accelerometer, corresponding to 3 to 5 metabolic equivalents<br><br>Con: No intervention	12	Received one dietary education program at the beginning of the intervention	Body weight, waist circumference, subcutaneous abdominal fat, subcutaneous fat, visceral adipose tissue
Kadoglouet et al, [85] 2010 Greece	Adults with type 2 diabetes mellitus and obesity	43 (63%)	50-70 (58.6)	30.6	Int: Supervised aerobic exercise training program consisting of four 30-45 min 4/ week (50 – 85% *V*O2max)	48	None	Body weight, fat mass percentage

© 2024 Jayedi A et al. *JAMA Network Open*.

Author[a], year, Country	Participants	Sample size (%female)	Age range (mean/ median)	Mean BMI (kg/m^2)	Exercise program	Intervention duration (weeks)	Co-intervention	Outcome(s)
					Con: Maintain their habitual activities			
Kadoglou et al, [86] 2007 Greece	Adults with type 2 diabetes mellitus and obesity	54 (60%)	(59.57)	30.9	Int: Supervised aerobic exercise training program consisting of four 45–60 min sessions per week (50 – 85% *V*O2max)<br><br>Con: Maintain their habitual activities	16	None	Body weight
Kang et al, [87] 2014 Korea	Older women with obesity	80 (100%)	(62)	24.8	Int: Supervised aerobic exercise in the trekking program, 3/week, and 90 min per session, at a moderate intensity.<br><br>Con: No intervention	12	None	Body weight, fat mass percentage
Kempen et al, [88] 1995 Netherlands	Healthy women with obesity	20 (100%)	20-50	31.9	Int: Supervised aerobic started with 15 min warm-up, followed by 60 min of aerobic and fitness at 50-60% VO2 max and ended in a 15-min cool-down, each session lasted 90 min 3/week<br><br>Con: No intervention	8	Energy-restriction program providing 2 MJ/d for 4 week and 3.5 MJ/d from 5 to 9 week	Body weight, fat mass
Kirk et al, [89] 2003 US	Young adults with overweight and obesity	74 (58%)	19-30 (22.8)	26.9	Int: Supervised aerobic exercise progressed from 3 day/week, 20 min session at 60% of heart rate reserve (HRR) to 5-day week, 45 min session at 75% of HRR at 6 months, and this level was maintained until 16 months<br><br>Con: Maintained their normal physical activity and ad libitum diets	64	None	Body weight, fat mass
Koo et al, [90] 2010 Korea	Women with Type 2 diabetes and obesity	31 (100%)	>18 (56)	28	Int: Supervised walk briskly for 120 min every day, which corresponds to an energy expenditure of approximately 500 kcal⁄day	12	None	Body weight, subcutaneous fat area, visceral fat area

© 2024 Jayedi A et al. *JAMA Network Open*.

Author[a], year, Country	Participants	Sample size (%female)	Age range (mean/ median)	Mean BMI (kg/m^2)	Exercise program	Intervention duration (weeks)	Co-intervention	Outcome(s)
					Con: Conventional education for a mild hypocaloric diet			
Kreamer et al, [91] 1998 US	Men with overweight	19 (0%)	>18 (37)	32.2	Int: Supervised aerobic endurance exercise designed to elicit a target heart rate of 70-80%. During the first week, each session lasted 30 min and gradually increased to 50 min over the subsequent weeks, 3/week<br><br>Con: No intervention	12	Behavior modification techniques and educating subjects aim to create a 6- to 9-kg weight loss	Body weight, fat mass, fat mass percentage
Ku et al, [92] 2010 Korea	Women with Type 2 diabetes and obesity	31 (100%)	38-68 (56.4)	27.2	Int: Supervised aerobic training group exercised by walking for 60 min at moderate intensity (3.6 – 5.2 metabolic equivalents, 5/week)<br><br>Con: Diabetes education and maintain their sedentary lifestyle	12	None	Body weight, waist circumference, subcutaneous adipose tissue in abdomen, visceral adipose tissue in abdomen
Lanting et al, [93] 2022 Australia	Adults with diabetes and obesity	21 (45%)	(56.1)	35.8	Int: Supervised aerobic training, sessions incorporated a 5 min warm up and 5 min cool down at 50% VO2peak either side of training and it progressed from 30 min at 50% VO2peak in week 1 to 45 min at 60% VO2peak by week 4<br><br>Con: Sham exercise placebo	12	None	Body weight, waist circumference
Lesser et al, [94] 2016 Canada	Postmenopausal South Asian women with obesity	49 (100%)	(57)	29.6	Int: Supervised aerobic program consisted of a 10-min group warm-up, 40 min of aerobic conditioning (treadmills and stationary bicycles), and a 10-min group cooldown, HR was	12	None	Body weight, waist circumference, body fat mass, body fat

© 2024 Jayedi A et al. *JAMA Network Open*.

Author[a], year, Country	Participants	Sample size (%female)	Age range (mean/ median)	Mean BMI (kg/m^2)	Exercise program	Intervention duration (weeks)	Co-intervention	Outcome(s)
					prescribed at 55% of HRmax and increased 10% every 3 weeks with the last 3 week of the program prescribed at 85% of HRmax<br><br>Con: Maintain their current physical activity			percentage, total abdominal adipose tissue, visceral adipose tissue, subcutaneous abdominal adipose tissue
Mager et al, [95] 2008 Finland	Patients with metabolic syndrome and obesity	33 (45%)	40-70 (60)	32.9	Int: Supervised aerobic program, at first a 4- to 8-week 30 min at low intensity per session, 2-3/week. After that, training frequency was increased to a minimum of 4/week for at least 30 min per session and at 55–65% of the maximum level<br><br>Con: Continue their normal lifestyle	33	None	Body weight, waist circumference
Makiel et al, [96] 2023 Australia	Males with Metabolic Syndrome with obesity	41 (0%)	30–45 (36.6)	33.8	Int: Supervised walking on treadmill consist of starting with a 5-min warm-up, not exceeding 50% HRmax, the intensity was elevated to 70% HR max duration was 45 min, 3/week<br><br>Con: No intervention	12	None	Body weight, Body fat mass
Marks et al, [97] 1995 US	In active women with overweight	18 (100%)	20- 49 (38.4)	29.4	Int: Supervised aerobic training including stationary ergometer, 3/week for at least 30 min per session at 70-80 HR max, the training program progressed from 12 to 36 minutes per session<br><br>Con: No intervention	20	Low-fat low-calorie diet (calorie intake was reduced 628 kcal/day)	Body weight, body fat mass, body fat percentage

© 2024 Jayedi A et al. *JAMA Network Open*.

Author[a], year, Country	Participants	Sample size (%female)	Age range (mean/ median)	Mean BMI (kg/m^2)	Exercise program	Intervention duration (weeks)	Co-intervention	Outcome(s)
Middlebrooke et al, [98] 2006 UK	Adults with type 2 diabetes mellitus and obesity	59 (46%)	40-75 (62.9)	30.4	Int: Supervised aerobic exercise program (30 min, 3/week, 70–80% of HRmax)<br><br>Con: standard care	24	None	Body weight, waist circumference
Moghadasi et al, [99] 2012 Iran	Sedentary middle-aged men with overweight and obesity	16 (0%)	(41)	31.5	Int: Supervised endurance training 4/ week at an intensity corresponding to 75–80% individual maximum oxygen consumption for 45 min<br><br>Con: Maintenance their physical activity and diet	12	None	Body weight, body fat mass, body fat percentage, central visceral and subcutaneous adipose tissue
Mohanka et al, [100] 2006 US	Postmenopausal women with overweight and obesity	171 (100%)	50-75 (60.4)	31.5	Int: Supervised moderate-intensity exercise each session at least 45 min 5/ week, training program started at 40% of observed HRmax for 16 min per session and gradually increased to 60–75% of HRmax for 45 min per session by week 8<br><br>Con: Attended one weekly 60-min stretching and asked not to change other exercise habits	12	None	Body weight, body fat percentage, intraabdominal fat, subcutaneous abdominal fat
Moraleda et al, [101] 2013 Spain	Adults with obesity	48 (49%)	18-50 (36.2)	32	Int: Supervised endurance training including treadmill, biking or cross trainer. In weeks 2-5 exercise was at an intensity of 50% of the 15 RM and HRR, and lasted an overall 51 min and 15 s<br><br>Con: Followed the habitual hospital clinical practice	24	Underwent a hypocaloric diet (between 1,200 and 3,000 kcal)	Body weight, body fat percentage
Nie et al, [102] 2017 China	Women with obesity	27 (100)	18-25 (20.9)	26.8	Int: Supervised continuous cycling exercise at an intensity of 60% $\dot{V}$O2 max, each session lasted 30 min and	12	None	Body weight, body fat mass,

© 2024 Jayedi A et al. *JAMA Network Open*.

Author[a], year, Country	Participants	Sample size (%female)	Age range (mean/ median)	Mean BMI (kg/m^2)	Exercise program	Intervention duration (weeks)	Co-intervention	Outcome(s)
					completed 10 min warm up and 5-min cool-down at 50–60% of HRmax, for the first 4 weeks, the training frequency was 3/ week. During weeks 5– 12, the training was frequency was increased to 4/ week.<br><br>Con: Maintenance their physical activity and diet			body fat percentage
Nishijima et al, [103]<br>2007<br>Japan	Adults with multiple cardiovascular risk factors and obesity	541<br>(60%)	40–89<br>(67)	26.4	Int: Supervised bicycle exercise (duration was initially set at 20 min, and an was increase it in steps up to 40 min) at 40% of the estimated VO2peak from the maximal exercise test at baseline; and it was made to increase the workload in two to three steps to 70% of the maximum with training effects, the duration of each exercise session was set at 60–90 min<br><br>Con: Follow lifestyle-modification advice	24	None	Body weight, waist circumference
Pavlou et al, [104]<br>1985<br>US	Male with mildly obesity	72<br>(0%)	26-52<br>(43.5)	NR	Int: Supervised exercise training consisted of a walk-jog type activity for the initial 2 week (70% HRmax) and later extended to walk-jog-run (85% HRmax) for the remaining 6 week, each session lasted 45 min 3/week<br><br>Con: No intervention	8	A diet provide 800 kcal/day energy intake supplemented with multivitamins	Body weight
Poon et al, [105]<br>2002<br>Hong Kong	Asian middle-aged men with obesity	20<br>(0%)	40-59<br>(42)	26.3	Int: Supervised brisk-walking at 65-70% HRmax 40-min for each session, 3/week	16	None	Body weight, waist circumference, body fat percentage

© 2024 Jayedi A et al. *JAMA Network Open*.

Author[a], year, Country	Participants	Sample size (%female)	Age range (mean/ median)	Mean BMI (kg/m^2)	Exercise program	Intervention duration (weeks)	Co-intervention	Outcome(s)
					Con: No specific exercise intervention			
Potteiger et al, [106] 2003 US	Individuals with overweight or moderate obesity	66 (57%)	17-35	29.7	Int: Supervised and verified exercise on 3 to 5 days per week in 20-to-45-minute sessions at 60% to 75% of heart rate reserve<br><br>Con: Maintain their normal physical activity and dietary intake patterns	64	None	Body weight, waist circumference, body fat percentage
Pugh et al, [107] 2014 UK	Nonalcoholic fatty liver disease with obesity	21 (48%)	(47.5)	30.3	Int: Supervised training began the with 30 min moderate-intensity aerobic exercise 3 times/week at 30% of heart rate reserve (HRR) for the initial 4 week. Intensity increased to 45% HRR for the following 4 week, until week 8, where HRR remained at 45%, but the duration of each session increased to 45 min. From week 12, participants were exercising 5 /week for 45 min at 60% of their individual HRR<br><br>Con: Lifestyle advice at clinical consultation	16	None	Body weight, waist circumference, body fat percentage, visceral adipose tissue, subcutaneous adipose tissue
Racette et al, [108] 1995 US	Healthy premenopausal women with obesity	30 (100%)	21-47 (39)	33.9	Int: supervised aerobic exercise including a treadmill, an air-dyne bicycle, a bicycle ergometer, a rowing ergometer, and stair-stepping machine, for 45 min on each of 3/week, the exercise intensity was set at 65% VO2max<br><br>Con: Maintain their normal physical activity	12	A reducing diet with energy intake approximately 75% of BMR to promote a weight loss of 1 kg/week	Body weight
Rezaeeshirazi et al, [109] 2021 Iran	Men with type-2 diabetes and obesity	28 (0%)	17-25 (21.8)	32.2	Int: Supervised aerobic program was fulfilled on the treadmill with intensity of 65% of the maximum aerobic power	8	None	Body weight, waist circumference,

© 2024 Jayedi A et al. *JAMA Network Open*.

Author[a], year, Country	Participants	Sample size (%female)	Age range (mean/ median)	Mean BMI (kg/m^2)	Exercise program	Intervention duration (weeks)	Co-intervention	Outcome(s)
					for 45 minutes on the first session, 4/week<br><br>Con: Maintain their normal physical activity and dietary pattern			fat mass, body fat percentage
Rezende et al, [110]<br>2016<br>Brazil	Postmenopausal women with nonalcoholic fatty liver disease and obesity	40 (100%)	(55)	33	Int: Supervised aerobic physical activity program composed of a 5-minute warm up followed by 30 to 50 minutes of treadmill aerobic exercise and 5 minutes of cooling down. Exercise sessions lasted 30 to 50 minutes, with increases in exercise duration every 8 weeks, 2/ week (approximately 120 min/week)<br><br>Con: No intervention	24	Followed diet produce an energy deficit of 500 kcal/d	Waist circumference, fat mass, body fat percentage
Rice et al, [111]<br>1999<br>Canada	Healthy men with obesity	19 (0%)	(45.9)	32.1	Int: Supervised aerobic exercise (walking on a motorized treadmill, stationary cycling, or stair stepping), initial duration of each exercise period was 19 min and gradually progressed to 60 min. Intensity of exercise progressed from 50 to 85% HRmax, 5/week<br><br>Con: No intervention	16	Calorie-restricted diet by 1,000 kcal/day	Body weight, waist circumference, subcutaneous adipose tissue, visceral adipose tissue
Roberson et al, [112]<br>2018<br>US	Older adults with obesity	15 (80%)	(69)	31	Int: Supervised treadmill exercise program at 55% of each participant's heart rate reserve, each session lasted 35 min, 3/week<br><br>Con: No intervention	12	None	Waist circumference
Ross et al, [113]<br>2000<br>Canada	Men with obesity	30 (0%)	>18 (44.3)	31.3	Int: Supervised aerobic training including brisk walking or light jogging on a motorized treadmill, at an	12	Reduce the calorie intake by 700 kcal/d	Body weight, body fat mass, Waist circumference,

© 2024 Jayedi A et al. *JAMA Network Open*.

Author[a], year, Country	Participants	Sample size (%female)	Age range (mean/ median)	Mean BMI (kg/m^2)	Exercise program	Intervention duration (weeks)	Co-intervention	Outcome(s)
					intensity 80% HRmax, approximately 60 minutes of daily exercise<br><br>Con: Maintain body weight throughout study			subcutaneous adipose tissue, visceral adipose tissue
Ross et al, [114] 2015 Canada	Adults with abdominal obesity	300 (65%)	(51.4)	33.4	Int: Supervised low-amount, low-intensity exercise (180 and 300 kcal/session for women and men, respectively, at 50% of V˙O2peak), 5/week<br><br>Con: Maintain their level of physical activity	24	None	Waist circumference
Ryan et al, [115] 2012 US	Postmenopausal women with overweight or obesity	96 (100)	49-76 (61.5)	32.7	Int: Supervised aerobic training, 3/week, at >85% heart rate reserve for 45 min<br><br>Con: maintain their current lifestyles	24	A calorie restricted diet	Body weight, waist circumference, body fat percentage, fat mass, visceral fat, subcutaneous fat
Saeidi et al, [116] 2021 Iran	Men with obesity	30 (0%)	(27.6)	32.6	Int: Supervised jogging or running on the treadmill at 70% of VO2peak, 36 min 3/ week<br><br>Con: maintain their current lifestyles	12	None	Body weight, body fat mass
Said et al, [117] 2020 Suadi Arabia	Young adults with obesity	30 (0%)	(21.7)	36.2	Int: Supervised aerobic training (walking on a treadmill, pedaling a stationary bike, or exercising on a stair-climber or elliptical machine) session routine progressed gradually in intensity [from 50%–55% to 65%–70% of the subject's HRmax], 4/week for 60 minutes each session	12	Maintaining a daily energy intake of less than the baseline energy intake of -500 kcal/day	Body weight, body fat mass, Waist circumference, body fat percentage

© 2024 Jayedi A et al. *JAMA Network Open*.

Author[a], year, Country	Participants	Sample size (%female)	Age range (mean/ median)	Mean BMI (kg/m^2)	Exercise program	Intervention duration (weeks)	Co-intervention	Outcome(s)
					Con: No intervention			
Saremi et al, [118] 2010 Iran	Middle-aged and sedentary men with overweight and obesity	21 (0%)	(44.3)	29.3	Int: Supervised aerobic training 50-60 min/d, Exercise intensity progressed from 15-20 min at 60–65% of HRmax during the first week to 25-30 min at 60–70% of HRmax by week 3, 35-40 min at 75-80% of HRmax by week 7 and 45-50 min at 80–85% of HRmax by week 12, 5/week<br><br>Con: No intervention	12	None	Body weight, waist circumference, body fat percentage, visceral fat, subcutaneous fat
Sarsan et al, [119] 2006 Turkey	Women with obesity	40 (100%)	20-60 (42.6)	35.4	Int: Supervised aerobic exercise including walk briskly for 15 min out of the door and then to exercise on a stationary leg cycle ergometer at 50-85% of the heart rate reserve. first month: 3/week for 12-15 min, second month: 4/week for 20-30 min, third month: 5/week<br><br>Con: No intervention	12	None	Body weight, waist circumference
Sigal et al, [120] 2007 Canada	Inactive adults with type 2 diabetes and obesity	123 (35%)	39-70 (54.3)	35.3	Int: Supervised aerobic training group on treadmills or bicycle ergometers. Training progressed from 15 to 20 minutes per session at 60% of the HRmax to 45 minutes per session at 75% of the HRmax, 3/week<br><br>Con: No intervention	22	Followed a diet did not cause weight loss	Body weight, waist circumference, body fat percentage, body fat mass, abdominal subcutaneous fat, abdominal visceral fat
Slentz et al, [121] 2005	Sedentary adults with	175 (51%)	40–65 (52.7)	29.6	Int: Supervised low amount, moderate intensity, equivalent to walking 12	32	None	Body weight, visceral fat,

© 2024 Jayedi A et al. *JAMA Network Open*.

Author[a], year, Country	Participants	Sample size (%female)	Age range (mean/ median)	Mean BMI (kg/m^2)	Exercise program	Intervention duration (weeks)	Co-intervention	Outcome(s)
US	overweight and mild to moderate dyslipidemia				miles/week at 40–55% of peak oxygen consumption, 204 min/week<br><br>Con: Maintained their normal diet and level of physical activity			subcutaneous fat
Soori et al, [122]<br>2017<br>Iran	Sedentary postmenopausal women with obesity	16<br>(100%)	45-60	≥30	Int: Supervised endurance training consisting of swimming or walking in the water, 3/week, 45 min per day, starting at 40% of HRmax. The training intensity was increased by 5% every other week<br><br>Con: No regular physical activity.	10	None	Body weight, fat mass percentage
Swift et al, [123]<br>2021<br>Canada	Adults with obesity	30<br>(82%)	40-65<br>(52.6)	36	Int: Supervised aerobic exercise training on a treadmill (3–4/ week) at 50%–75% of VO2peak, 130.8 min/week in month1, 151.9 min/week in month 2, 146.5 min/week in month 3, 128.4 min/week in month 4, 142.5 min/week in month 5, 134.4 min/week in month 6<br><br>Con: No intervention	24	None	Body weight, waist circumference
Tok et al, [124]<br>2021<br>Turkey	Adults with impaired glucose metabolism and obesity	48<br>(58%)	18-60<br>(40.4)	31.3	Int: Supervised aerobic activity moderate to high intensity (60–65%) weekly (30 min/day for 5 days in a week), 150 min/ week<br><br>Con: No intervention	12	Calorie-restricted diet (-750 kcal/d)	Body weight, waist circumference, body fat percentage
Tokeranko et al, [125]<br>2021<br>Ukraine	Sedentary adults with overweight and obesity	69<br>(46%)	29-57<br>(41.2)	33.4	Int: Supervised aerobic exercise training program included mainly of walking or running on a treadmill, cycling, and calisthenics involving upper and lower limb consisting of 45–60 min sessions 4/ week (50–85% VO2peak)	12	None	Body weight

© 2024 Jayedi A et al. *JAMA Network Open*.

Author[a], year, Country	Participants	Sample size (%female)	Age range (mean/ median)	Mean BMI (kg/m^2)	Exercise program	Intervention duration (weeks)	Co-intervention	Outcome(s)
					Con: Maintained their normal diet and level of physical activity			
Tseng et al, [126]<br>2013<br>Taiwan	Men with obesity	20<br>(0%)	18- 29<br>(22.1)	31	Int: Supervised aerobic-training group performed 60 min/day, 5 /week, from 15 min/session at 50–60% of HRmax to 45 min/session at 60–70% of HRmax during Weeks 1–12<br><br>Con: Maintained their normal diet and level of physical activity	12	None	Body weight, waist circumference
Utter et al, [127]<br>1998<br>US	Women with obesity	43<br>(100%)	25-76<br>(44.9)	32.3	Int: Supervised walking, 45 min per session, at 60-80% HRmax, 5/week, duration and intensity were gradually increased over 3 weeks from 25- 30 min/ session at 60-65 HRmax during first week to 45 min at 70-80% HRmax weeks 4 through 12<br><br>Con: 4/week for 45 min of stretching and mild range of motion calisthenic exercise	12	None	Body weight, waist circumference, body fat percentage, body fat mass
van Aggel-Leijssen et al, [128]<br>2021<br>Netherlands	Healthy men with obesity	23<br>(0%)	>18<br>(42.2)	31.2	Int: Supervised aerobic training consisted of cycling on an ergometer at low intensity 40% VO2max, 3/ week<br><br>Con: No intervention	12	None	Body weight, fat mass percentage
Venojärvi et al, [129]<br>2013<br>Finland	Middle-aged men with impaired glucose regulation with obesity	79<br>(0%)	40-65<br>(54.5)	29.1	Int: Supervised walking, sessions were carried out at strain levels increasing from 55% to 75% of heart rate reserve (weeks 1–4 at 55%, weeks 5 – 8 at 65%, and weeks 9 – 12 at 75%), 60 min per session 3/week<br><br>Con: No intervention	12	None	Body weight, waist circumference, body fat mass, fat mass percentage

© 2024 Jayedi A et al. *JAMA Network Open*.

Author[a], year, Country	Participants	Sample size (%female)	Age range (mean/ median)	Mean BMI (kg/m^2)	Exercise program	Intervention duration (weeks)	Co-intervention	Outcome(s)
Verity et al, [130] 1988 US	Postmenopausal women with NIDDM and obesity	10 (100%)	50- 70 (59.2)	30	Int: Supervised walking at 65- 80% of cardiac reserve, 1-1.5h 3/week<br><br>Con: No intervention	16	None	Body weight, body fat percentage
Villareal et al, [131] 2017 US	Elderly with obesity	80 (67%)	>65 (70)	36.3	Int: Supervised aerobic exercise training consisted of treadmill walking, stationary cycling, and stair climbing, sessions were approximately 60 minutes long and included 10 minutes of flexibility exercises, followed by 40 minutes of aerobic exercises and 10 minutes of balance exercises. The intensity was at approximately 65% of their peak heart rate, which was gradually increased to 70 to 85%, 3/week<br><br>Con: Attended group educational sessions about a healthful diet during monthly visits	26	Nutritional counseling to achieve a 500–750 kcal deficit in energy requirements per day and 1 g protein/kg body weight per day	Body weight, body fat mass
Wang et al, [132] 2017 China	Adults with obesity	24 (NR)	>18	>28	Int: Supervised aerobic exercises such as jogging at 60%-70% of HRmax, 3/week, 60 min per times<br><br>Con: No intervention	16	None	Body weight, body fat mass, body fat percentage
Womack et al, [133] 2000 US	Older men with obesity	70 (0%)	46–80 (58)	30.5	Int: Supervised training predominantly on treadmills and occasionally cycle ergometers, 30–45 minutes 3/ week. The initial intensity of the exercise was set at 50%–60% of the subject's heart rate reserve. Exercise intensity was gradually increased to 70%–80% of heart rate reserve for 30–45 minutes per session.	36	None	Body weight, waist circumference, body fat percentage

© 2024 Jayedi A et al. *JAMA Network Open*.

Author[a], year, Country	Participants	Sample size (%female)	Age range (mean/ median)	Mean BMI (kg/m^2)	Exercise program	Intervention duration (weeks)	Co-intervention	Outcome(s)
					Con: Consume an isocaloric AHA Phase I diet and maintained their normal diet and level of physical activity			
Wood et al, [134] 1991 US	Adults with overweight and obesity	177 (50%)	25-49 (39.7)	29.3	Int: Supervised aerobic exercise (primarily brisk walking and jogging) continuously at 60-80% HRmax for at least 25 min initially and gradually increased to 45 min by the fourth month of study, 3/week<br><br>Con: No change in exercise level	48	Followed a prudent diet with concomitant reduction	Body weight, body fat mass
Wu et al, [135] 2017 US	Premenopausal Women with obesity	23 (100%)	(28.2)	34.4	Int: Supervised low intensity training at 50–56% VO2max, the average running time per session were 34.5± 1.5 min and 41.8 ± 1.95 min in Mexican American and Korean respectively 5/week<br><br>Con: Maintained their normal diet and level of physical activity	12	None	Body weight, body fat mass, body fat percentage
You et al, [136] 2004 US	Healthy, nonsmoking postmenopausal women with obesity	34 (100%)	50-70 (58)	32.8	Int: Supervised walked on a treadmill 3 d/week, the duration and intensity of the exercise progressed from 20 min at 50–55% of HRR during the first week of exercise to 45–60 min at 65–70% HRR by the third month<br><br>Con: No intervention	24	Followed a hypocaloric diet designed to elicit a 0.5- to 1.0-kg weight loss/week (250–350 kcal/d deficit)	Body weight, body fat mass, fat percentage, visceral fat area, subcutaneous fat area
Zhang et al, [137] 2015 China	Women with obesity	23 (100%)	>18 (20.8)	25.7	Int: Supervised moderate-intensity continuous training, 33-minute running at 60–70% HRpeak, 4/week	12	None	Body weight, waist circumference, body fat percentage, abdominal visceral fat

© 2024 Jayedi A et al. *JAMA Network Open*.

Author[a], year, Country	Participants	Sample size (%female)	Age range (mean/ median)	Mean BMI (kg/m^2)	Exercise program	Intervention duration (weeks)	Co-intervention	Outcome(s)
					Con: Maintained their normal diet and level of physical activity			area, abdominal subcutaneous fat area
Zhang et al, [138] 2017 China	Young women with obesity	28 (100%)	18-22 (20.9)	NR	Int: Moderate-intensity continuous training on a cycle ergometer at an intensity of 60% VO˙2max until the targeted 300 kJ of work was achieved; session duration 51.2 ± 5.8 at week 1-4, 74.4 ± 10.5 at 4-8 week, 62.6 ± 5.0 at 8-12; 4/week<br><br>Con: No intervention	12	None	Body weight, body fat percentage, abdominal visceral fat area, abdominal subcutaneous fat area
Zhang et al, [139] 2016 China	Adults with non-alcoholic fatty liver disease and obesity	220 (68%)	40- 65 (53.9)	28	Int: Supervised vigorous-moderate exercise (jogging 150 minutes per week at 65%-80% of maximum heart rate for 6 months and brisk walking 150 minutes per week at 45%-55% of maximum heart rate for another 6 months), or moderate exercise (brisk walking 150 minutes per week for 12 months)<br><br>Con: Maintained their normal diet and level of physical activity	48	Education sessions	Body weight, waist circumference, body fat mass, body fat percentage, Visceral fat, Subcutaneous fat
Zhao et al, [140] 2021 China	Men with overweight and obesity	45 (0%)	>18 (50.1)	28.4	Int: Supervised moderate-intensity aerobic exercise program consisting of a 90-minute session for 12 weeks, 3/week<br><br>Con: No intervention	12	Participants were taught how to restrict their calorie intake to 1680 kcal/day	Body weight, waist circumference, body fat percentage
Donnelly et al, [141] 2013 US	Adults with overweight and obesity	92 (50%)	18-30	29.8	Int: Supervised aerobic exercise including a walking/jogging on motor-driven treadmills, each session ~45 min at 70-80% HRmax, 5/week	40	None	Body weight, fat mass, body fat percentage

© 2024 Jayedi A et al. *JAMA Network Open*.

Author[a], year, Country	Participants	Sample size (%female)	Age range (mean/ median)	Mean BMI (kg/m^2)	Exercise program	Intervention duration (weeks)	Co-intervention	Outcome(s)
					Con: Maintain physical activity and dietary intake patterns			

Abbreviations: HRmax, maximum heart rate; HRR, heart rate reserve; VO2Max, volume oxygen maximum.
[a] Numbers pertain to the reference numbers cited in the main text.

eTable 5. Dropout, degree of adherence to the intervention program, and adverse events in the trials included in the meta-analysis of aerobic exercise and measures of body weight, waist, and fat

Author[a], year	Dropout, n (%)	Adherence	Adverse events
Abdelaal et al, [26] 2014	0	Not reported	No adverse events were recorded during the study
Ahmadi et al, [27] 2021	0	Not reported	Not assessed
Ahmadizad et al, [28] 2007	0	Not reported	Not assessed
Akbarpoor et al, [29] 2013	0	Not reported	Not assessed
Alvez et al, [30] 2009	Control: 3 (4%)<br>Intervention: 7 (9%)	Not reported	Not assessed
Amanat et al, [31] 2020	Control: 1 (7%)<br>Intervention: 1 (7%)	Not reported	Not assessed
Aminlari et al, [32] 2017	Control: 0<br>Intervention: 3 (25%)	Not reported	Not assessed
Anderssen et al, [33] 2007	0	61.4%	Not assessed
Armannia et al, [34] 2022	Control: 2 (14%)<br>Intervention: 2 (14%)	Not reported	Not assessed
Arsenault et al, [35] 2009	Control: 6 (6%)<br>Intervention: 31 (9%)	92%	The 4 individuals who dropped out of the study because of injury were in the intervention groups.
Arslan et al, [36] 2017	Control: 3 (12%)<br>Intervention: 5 (19%)	Not reported	Not assessed
Auerbach et al, [37] 2013	Control: 0<br>Intervention: 4 (25%)	Excellent	Not assessed
Baria et al, [38] 2014	Control: 1 (10%)<br>Intervention: 1 (5%)	Not reported	Not assessed
Beavers et al, [39] 2017	Control: 9 (11%)<br>Intervention: 9 (10%)	83%	Those four events possibly related to the interventions included a critically low fasting glucose of 11 mg/dL that was detected following a testing visit; surgical repair of torn meniscus in the knee; diagnosis of bulging disc in conjunction with spinal stenosis; and intensification of shoulder pain while the intervention.
Bell et al, [40] 2010	Control: 26 (38%)<br>Intervention: 26 (37%)	92%	Not assessed
Belli et al, [41] 2011	Control: 2 (17%)<br>Intervention: 3 (25%)	92%	Not assessed
Benito et al, [42] 2015	Control: 8 (27%)<br>Intervention: 4 (13%)	>80%	Not assessed
Bertram et al, [43] 1990	Control: 7 (47%)<br>Intervention: 2 (13%)	Not reported	Not assessed
Blond et al, [44] 2018	Control: 2 (11%)<br>Intervention: 13 (17%)	High (>85%)	Not assessed
Blumenthal et al, [45] 2000	Control: 2 (8%)<br>Intervention: 10 (19%)	77%	One patient in the intervention group did not complete the study due to increase in blood pressure.
Chin et al, [46] 2019	Control: 13 (48%)<br>Intervention: 9 (43%)	High	One knee and ankle injury in the intervention group
Chiu et al, [47] 2017	Control: 2 (14%)<br>Intervention: 3 (8%)	Not reported	Not assessed
Cho et al, [48]	Control: 5 (33%)	80%	Not assessed

Author[a], year	Dropout, n (%)	Adherence	Adverse events
2011	Intervention: 3 (17%)		
Chow et al, [49] 2020	Control: 2 (17%) Intervention: 3 (13%)	Not reported	Not assessed
Christiansen et al, [50] 2009	Control: 6 (21%) Intervention: 4 (16%)	92%	Not assessed
Coker et al, [51] 2009	0	Not reported	Not assessed
Cooper et al, [52] 2016	Control: 1 (7%) Intervention: 0	91%	Not assessed
Cornish et al, [53] 2021	0	Not reported	Not assessed
Cuff et al, [54] 2023	Control: 1 (10%) Intervention: 1 (10%)	Not reported	Not assessed
Dash et al, [55] 2018	Control: 16 (22.5%) Intervention: 24 (33%)	65%	Not assessed
Davidson et al, [56] 2009	Control: 5 (23%) Intervention: 7 (19%)	Excellent	2 had knee/arthritis pain in the control group and 1 had knee/arthritis pain and 1 had back pain in the intervention group
Dengel et al, [57] 1996	0	Not reported	Not assessed
Donges et al, [58] 2013	5 (19%)	92%	Not assessed
Donnelly et al, [59] 2003	Control: 11 (25%) Intervention: 46 (53%)	90%	1 injury in the intervention group
Eizadi et al, [60] 2013	0	Not reported	Not assessed
Elsayed et al, [61] 2022	Control: 2 (6%) Intervention: 3 (9%)	Not reported	No adverse events related to the intervention were reported in either group during the study
Ezpeletka et al, [62] 2023	Control: 0 Intervention: 5 (25%)	93%	Not assessed
Fenkci et al, [63] 2006	Control: 3 (15%) Intervention: 3 (15%)	Not reported	Not assessed
Fisher et al, [64] 2011	83 (39%)	Not reported	Not assessed
Fogelholm et al, [65] 2000	Control: 1 (3%) Intervention: 1 (2%)	Good	Not assessed
Foster-Schubert et al, [66] 2012	Control: 7 (8%) Intervention: 9 (8%)	Excellent	Not reported
Fu et al, [67] 2022	9 (7%)	Not reported	Not assessed
Geliebter et al, [68] 1997	0	Not reported	Not assessed
Giannopoulou et al, [69] 2005	7 (17.5%)	Not reported	Not assessed
Goodpaster et al, [70] 2010	Control: 11 (17%) Intervention: 18 (27%)	High	Assessed but not reported
Gram et al, [71] 2017	Control: 2 (11%) Intervention: 22 (29%)	High	Two adverse events occurred in the intervention arm
Gram et al, [72] 2010	Control: 0 Intervention: 1 (5%)	63.5%	One hypoglycemic event was observed during a Nordic walking training session in an insulin-treated participant and 1 subject had musculoskeletal symptoms
Gulsin et al, [73]	Control: 0	Not reported	Not assessed

© 2024 Jayedi A et al. *JAMA Network Open*.

Author[a], year	Dropout, n (%)	Adherence	Adverse events
2020	Intervention: 9 (29%)		
Guzel et al, [74] 2022	0	91%	Not assessed
Hara et al, [75] 2005	0	Not reported	Not assessed
Hays et al, [76] 2006	Control: 1 (9%)<br>Intervention: 1 (9%)	Not reported	Not assessed
Herzig et al, [77] 2014	Control: 5 (13%)<br>Intervention: 1 (13%)	67%	Not assessed
Ho et al, [78] 2014	Control: 3 (16%)<br>Intervention: 4 (21%)	Not reported	Not assessed
Hong et al, [79] 2014	0	Not reported	Not assessed
Irwin et al, [80] 2003	Control: 0<br>Intervention: 6 (7%)	Excellent	Not assessed
Jang et al, [81] 2019	0	Not reported	Not assessed
Janssen et al, [82] 1999	0	90.5%	Not assessed
Jung et al, [83] 2012	16 (36%)	Not reported	Not assessed
Jung et al, [84] 2014	0	Not reported	Not assessed
Kadoglouet et al, [85] 2010	Control: 4 (16%)<br>Intervention: 3 (12%)	Not reported	Not assessed
Kadoglou et al, [86] 2007	Control: 4 (13%)<br>Intervention: 2 (7%)	Not reported	Not assessed
Kang et al, [87] 2014	Control: 2 (4%)<br>Intervention: 18 (36%)	Not reported	Not assessed
Kempen et al, [88] 1995	0	Not reported	Not assessed
Kirk et al, [89] 2003	Control: 46 (53%)<br>Intervention: 11 (25%)	90%	No major adverse events occurred during this study for either the exercise or control group
Koo et al, [90] 2010	6 (9%)	Not reported	Not assessed
Kreamer et al, [91] 1998	Not reported	Not reported	Not assessed
Ku et al, [92] 2010	Not reported	Not reported	Not assessed
Lanting et al, [93] 2022	Control: 0<br>Intervention: 1 (9%)	Not reported	Not assessed
Lesser et al, [94] 2016	0	78%	Not assessed
Mager et al, [95] 2008	0	Not reported	Not assessed
Makiel et al, [96] 2023	Control: 3 (15%)<br>Intervention: 3 (14%)	Not reported	Not assessed
Marks et al, [97] 1995	3 (4%)	Not reported	Not assessed
Middlebrooke et al, [98] 2006	Control: 0<br>Intervention: 7 (24%)	99%	Not assessed
Moghadasi et al, [99]	0	Not reported	Not assessed

© 2024 Jayedi A et al. *JAMA Network Open*.

Author[a], year	Dropout, n (%)	Adherence	Adverse events
2012			
Mohanka et al, [100] 2006	Control: 0<br>Intervention: 2 (2%)	Excellent	Not assessed
Moraleda et al, [101] 2013	Control: 8 (26%)<br>Intervention: 4 (13%)	89%	Not assessed
Nie et al, [102] 2017	Control: 3 (19%)<br>Intervention: 1 (7%)	96%	No adverse events occurred during this study for either the exercise or control group
Nishijima et al, [103] 2007	Control: 28 (10%)<br>Intervention: 28 (10%)	55%	Not assessed
Pavlou et al, [104] 1985	38 (34%)	94%	Not assessed
Poon et al, [105] 2002	Control: 2 (17%)<br>Intervention: 2 (17%)	84%	No adverse events occurred during this study for either the exercise or control group
Potteiger et al, [106] 2003	65 (50%)	90%	Not assessed
Pugh et al, [107] 2014	Control: 5 (38%)<br>Intervention: 5 (28%)	Not reported	Not assessed
Racette et al, [108] 1995	Control: 2 (38%)<br>Intervention: 4 (28%)	Not reported	Not assessed
Rezaeeshirazi et al, [109] 2021	Control: 2<br>Intervention: 2 (13%)	Not reported	Not assessed
Rezende et al, [110] 2016	Control: 2 (9%)<br>Intervention: 2 (10%)	Not reported	Not assessed
Rice et al, [111] 1999	7 (17.5%)	92%	Not assessed
Roberson et al, [112] 2018	Control: 2 (20%)<br>Intervention: 3 (30%)	83%	Not assessed
Ross et al, [113] 2000	Control: 3 (27%)<br>Intervention: 2 (13%)	98%	Not assessed
Ross et al, [114] 2015	Control: 22 (29%)<br>Intervention: 61 (27%)	87%	Not available
Ryan et al, [115] 2012	Control: 20 (23%)<br>Intervention: 28 (32%)	87%	Not assessed
Saeidi et al, [116] 2021	Control: 2 (12%)<br>Intervention: 2 (12%)	Not reported	Not assessed
Said et al, [117] 2020	Control: 1 (7%)<br>Intervention: 2 (13%)	89%	Not assessed
Saremi et al, [118] 2010	Control: 2 (17%)<br>Intervention: 2 (15%)	Not reported	Not assessed
Sarsan et al, [119] 2006	Control: 4 (17%)<br>Intervention: 6 (23%)	Not reported	Not assessed
Sigal et al, [120] 2007	Control: 3 (5%)<br>Intervention: 12 (20%)	80%	Four participants in the intervention arm reported adverse events
Slentz et al, [121] 2005	155 (53%)	89%	Not assessed
Soori et al, [122] 2017	0	Not reported	Not assessed
Swift et al, [123] 2021	0	90%	Not assessed
Tok et al, [124] 2021	Control: 6 (20%)<br>Intervention: 6 (20%)	Subjects with poor adherence were excluded	Not assessed

© 2024 Jayedi A et al. *JAMA Network Open*.

Author[a], year	Dropout, n (%)	Adherence	Adverse events
Tokeranko et al, [125] 2021	0	Not reported	Not assessed
Tseng et al, [126] 2013	Control: 1 (9%)<br>Intervention: 1 (9%)	Not reported	Not assessed
Utter et al, [127] 1998	11 (11%)	84%	Not assessed
van Aggel-Leijssen et al, [128] 2021	0	89%	Not assessed
Venojärvi et al, [129] 2013	Control: 7 (18%)<br>Intervention: 9 (19%)	64%	Not assessed
Verity et al, [130] 1988	0	Not reported	Not assessed
Villareal et al, [131] 2017	Control: 4 (10%)<br>Intervention: 5 (13%)	High	19 adverse events were recorded in the intervention group and 9 in the control group
Wang et al, [132] 2017	0	Not reported	Not assessed
Womack et al, [133] 2000	Control: 8 (31%)<br>Intervention: 19 (27%)	>75%	Not assessed
Wood et al, [134] 1991	33 (13%)	Not reported	Not assessed
Wu et al, [135] 2017	0	High	Not assessed
You et al, [136] 2004	14 (28%)	Not reported	Not assessed
Zhang et al, [137] 2015	Control: 3 (21%)<br>Intervention: 3 (20%)	90%	Not assessed
Zhang et al, [138] 2017	Control: 2 (13%)<br>Intervention: 1 (16%)	95%	No adverse events were reported during testing or training in either group
Zhang et al, [139] 2016	Control: 1 (1%)<br>Intervention 1: 4 (5%)<br>Intervention 2: 7 (10%)	96%	No deaths or serious adverse events were reported throughout the study
Zhao et al, [140] 2021	0	Not reported	Not assessed
Donnelly et al, [141] 2013	Control: 8 (32%)<br>Intervention: 41 (37%)	91%	Not assessed

[a] Numbers pertain to the reference numbers cited in the main text.

© 2024 Jayedi A et al. *JAMA Network Open*.

eTable 6. Risk of bias of the trials included in the meta-analysis of aerobic exercise and measures of body weight, waist and fat

Author[a], Year	Bias arising from the randomization process	Bias due to deviations from the intended interventions	Bias due to missing outcome data	Bias in the measurement of the outcome	Bias in the selection of the reported result	Overall risk of bias
Abdelaal et al, [26] 2014	Low risk	High risk	Low risk	Low risk	Some concern	High risk
Ahmadi et al, [27] 2021	High risk	High risk	Low risk	Low risk	Low risk	High risk
Ahmadizad et al, [28] 2007	Some concern	High risk	Low risk	Some concern	Some concern	High risk
Akbarpoor et al, [29] 2013	Some concern	High risk	Low risk	Some concern	Some concern	High risk
Alvez et al, [30] 2009	High risk	Some concern	Low risk	Low risk	Low risk	High risk
Amanat et al, [31] 2020	Low risk	Some concern	Low risk	Some concern	Low risk	Some concern
Aminlari et al, [32] 2017	Some concern	Some concern	Low risk	Some concern	Low risk	Some concern
Anderssen et al, [33] 2007	Some concern	Some concern	Low risk	Low risk	Low risk	Some concern
Armannia et al, [34] 2022	Low risk	Some concern	Some concern	Some concern	Low risk	Some concern
Arsenault et al, [35] 2009	Low risk	Low risk	Low risk	Low risk	Low risk	Low risk
Arslan et al, [36] 2017	Some concern	High risk	Low risk	Some concern	Some concern	High risk
Auerbach et al, [37] 2013	Some concern	Low risk	Low risk	Low risk	Low risk	Some concern
Baria et al, [38] 2014	Some concern	Some concern	Low risk	Low risk	Low risk	Some concern
Beavers et al, [39] 2017	Low risk	Some concern	Low risk	Low risk	Low risk	Some concern
Bell et al, [40] 2010	Some concern	Low risk	High risk	Low risk	Some concern	High risk
Belli et al, [41] 2011	Low risk	Low risk	Some concern	Some concern	Some concern	Some concern
Benito et al, [42] 2015	Low risk	Low risk	Low risk	Low risk	Low risk	Low risk
Bertram et al, [43] 1990	Some concern	High risk	High risk	Some concern	Some concern	High risk
Blond et al, [44] 2018	Low risk	Some concern	Low risk	Low risk	Low risk	Some concern
Blumenthal et al, [45] 2000	Some concern	Low risk	Low risk	Some concern	Low risk	Some concern
Chin et al, [46] 2019	Some concern	Low risk	High risk	Some concern	Low risk	High risk
Chiu et al, [47] 2017	Some concern	Some concern	Low risk	Some concern	Low risk	Some concern
Cho et al, [48] 2011	Low risk	Low risk	Low risk	Low risk	Low risk	Low risk

© 2024 Jayedi A et al. *JAMA Network Open*.

Author[a], Year	Bias arising from the randomization process	Bias due to deviations from the intended interventions	Bias due to missing outcome data	Bias in the measurement of the outcome	Bias in the selection of the reported result	Overall risk of bias
Chow et al, [49] 2020	Some concern	Some concern	Low risk	Low risk	Low risk	Some concern
Christiansen et al, [50] 2009	Some concern	Low risk	Low risk	Low risk	Some concern	Some concern
Coker et al, [51] 2009	Some concern	High risk	Low risk	Low risk	Low risk	High risk
Cooper et al, [52] 2016	Some concern	Low risk	Low risk	Low risk	Low risk	Some concern
Cornish et al, [53] 2021	Low risk	High risk	Some concern	Low risk	Low risk	High risk
Cuff et al, [54] 2023	Some concern	Low risk	Low risk	Low risk	Low risk	Some concern
Dash et al, [55] 2018	Low risk	Some concern	Low risk	Low risk	Low risk	Some concern
Davidson et al, [56] 2009	Low risk	Low risk	Low risk	Low risk	Low risk	Low risk
Dengel et al, [57] 1996	Some concern	High risk	Low risk	Some concern	Low risk	High risk
Donges et al, [58] 2013	Some concern	Low risk	Low risk	Low risk	Low risk	Some concern
Donnelly et al, [59] 2003	Some concern	Low risk	Some concern	Low risk	Low risk	Some concern
Eizadi et al, [60] 2013	Some concern	Low risk	High risk	High risk	Some concern	High risk
Elsayed et al, [61] 2022	Low risk	High risk	Low risk	Low risk	Low risk	High risk
Ezpeletka et al, [62] 2023	Low risk	Low risk	Low risk	Low risk	Low risk	Low risk
Fenkci et al, [63] 2006	Some concern	High risk	Low risk	Some concern	Some concern	High risk
Fisher et al, [64] 2011	Some concern	High risk	High risk	Low risk	Low risk	High risk
Fogelholm et al, [65] 2000	Some concern	Some concern	Low risk	Some concern	Low risk	Some concern
Foster-Schubert et al, [66] 2012	Low risk	Low risk	Low risk	Low risk	Low risk	Low risk
Fu et al, [67] 2022	Some concern	High risk	Low risk	Low risk	Low risk	High risk
Geliebter et al, [68] 1997	Low risk	High risk	Low risk	Some concern	Low risk	High risk
Giannopoulou et al, [69] 2005	Some concern	High risk	Low risk	Low risk	Some concern	High risk
Goodpaster et al, [70] 2010	Low risk	Low risk	Low risk	Low risk	Low risk	Low risk
Gram et al, [71] 2017	Some concern	Low risk	Some concern	Low risk	Low risk	Some concern
Gram et al, [72] 2010	Low risk	High risk	Low risk	Low risk	Low risk	High risk
Gulsin et al, [73] 2020	Some concern	Some concern	Low risk	Low risk	Low risk	Some concern

© 2024 Jayedi A et al. *JAMA Network Open*.

Author[a], Year	Bias arising from the randomization process	Bias due to deviations from the intended interventions	Bias due to missing outcome data	Bias in the measurement of the outcome	Bias in the selection of the reported result	Overall risk of bias
Guzel et al, [74] 2022	Some concern	Low risk	Low risk	Low risk	Low risk	Some concern
Hara et al, [75] 2005	Some concern	High risk	Low risk	Some concern	Some concern	High risk
Hays et al, [76] 2006	Some concern	High risk	Low risk	Low risk	Low risk	High risk
Herzig et al, [77] 2014	Some concern	Some concern	Low risk	Some concern	Low risk	Some concern
Ho et al, [78] 2014	Some concern	High risk	High risk	Low risk	Low risk	High risk
Hong et al, [79] 2014	Some concern	High risk	Low risk	Low risk	Low risk	High risk
Irwin et al, [80] 2003	Low risk	Low risk	Low risk	Low risk	Low risk	Low risk
Jang et al, [81] 2019	Some concern	High risk	Low risk	Some concern	Low risk	High risk
Janssen et al, [82] 1999	Some concern	Low risk	Low risk	Low risk	Low risk	Some concern
Jung et al, [83] 2012	Some concern	High risk	High risk	Low risk	Some concern	High risk
Jung et al, [84] 2014	High risk	High risk	Low risk	Low risk	Some concern	High risk
Kadoglouet et al, [85] 2010	Some concern	Low risk	Low risk	Some concern	Low risk	Some concern
Kadoglou et al, [86] 2007	Some concern	High risk	Low risk	High risk	Low risk	High risk
Kang et al, [87] 2014	Some concern	High risk	High risk	Some concern	Some concern	High risk
Kempen et al, [88] 1995	Some concern	High risk	Low risk	Low risk	Some concern	High risk
Kirk et al, [89] 2003	Some concern	Low risk	High risk	Some concern	Low risk	High risk
Koo et al, [90] 2010	Some concern	Low risk	Low risk	Low risk	Some concern	Some concern
Kreamer et al, [91] 1998	Some concern	High risk	High risk	High risk	Some concern	High risk
Ku et al, [92] 2010	Some concern	High risk	Low risk	Low risk	Low risk	High risk
Lanting et al, [93] 2022	Low risk	Low risk	Low risk	Low risk	Low risk	Low risk
Lesser et al, [94] 2016	Low risk	Some concern	Low risk	Low risk	Low risk	Some concern
Mager et al, [95] 2008	Some concern	High risk	Low risk	Low risk	Low risk	High risk
Makiel et al, [96] 2023	Some concern	High risk	Low risk	Low risk	Low risk	High risk
Marks et al, [97] 1995	Some concern	Some concern	Low risk	High risk	Low risk	High risk
Middlebrooke et al, [98] 2006	Some concern	Low risk	Low risk	Low risk	Low risk	Some concern

© 2024 Jayedi A et al. *JAMA Network Open*.

Author[a], Year	Bias arising from the randomization process	Bias due to deviations from the intended interventions	Bias due to missing outcome data	Bias in the measurement of the outcome	Bias in the selection of the reported result	Overall risk of bias
Moghadasi et al, [99] 2012	Some concern	Low risk	Low risk	Low risk	Low risk	Some concern
Mohanka et al, [100] 2006	Low risk	Low risk	Low risk	Low risk	Low risk	Low risk
Moraleda et al, [101] 2013	Some concern	Low risk	Some concern	Low risk	Low risk	Some concern
Nie et al, [102] 2017	Some concern	Low risk	Low risk	Some concern	Low risk	Some concern
Nishijima et al, [103] 2007	Low risk	Low risk	Low risk	Low risk	Low risk	Low risk
Pavlou et al, [104] 1985	Some concern	High risk	Low risk	Some concern	Some concern	High risk
Poon et al, [105] 2002	Low risk	Low risk	Low risk	Some concern	Low risk	Some concern
Potteiger et al, [106] 2003	High risk	Low risk	Low risk	Low risk	Low risk	High risk
Pugh et al, [107] 2014	Low risk	Low risk	Some concern	Low risk	Low risk	Some concern
Racette et al, [108] 1995	Some concern	High risk	Some concern	Low risk	Low risk	High risk
Rezaeeshirazi et al, [109] 2021	Some concern	High risk	Low risk	Some concern	Low risk	High risk
Rezende et al, [110] 2016	Some concern	High risk	Low risk	Some concern	Low risk	High risk
Rice et al, [111] 1999	Some concern	Low risk	Some concern	Low risk	Low risk	Some concern
Roberson et al, [112] 2018	Some concern	Low risk	Low risk	Some concern	Low risk	Some concern
Ross et al, [113] 2000	Some concern	Low risk	Low risk	Low risk	Low risk	Some concern
Ross et al, [114] 2015	Low risk	Low risk	Low risk	Low risk	Low risk	Low risk
Ryan et al, [115] 2012	Some concern	Low risk	Low risk	Low risk	Low risk	Some concern
Saeidi et al, [116] 2021	Some concern	High risk	Low risk	Some concern	Some concern	High risk
Said et al, [117] 2020	Low risk	Low risk	Low risk	Some concern	Low risk	Some concern
Saremi et al, [118] 2010	Some concern	Low risk	Low risk	Some concern	Low risk	Some concern
Sarsan et al, [119] 2006	Low risk	High risk	Low risk	Low risk	Some concern	High risk
Sigal et al, [120] 2007	Low risk	Low risk	Low risk	Some concern	Low risk	Some concern
Slentz et al, [121] 2005	Some concern	Low risk	Some concern	Low risk	Low risk	Some concern
Soori et al, [122] 2017	Some concern	High risk	Low risk	Low risk	Some concern	High risk
Swift et al, [123] 2021	Low risk	Low risk	Low risk	Low risk	Low risk	Low risk

© 2024 Jayedi A et al. *JAMA Network Open*.

Author[a], Year	Bias arising from the randomization process	Bias due to deviations from the intended interventions	Bias due to missing outcome data	Bias in the measurement of the outcome	Bias in the selection of the reported result	Overall risk of bias
Tok et al, [124] 2021	Some concern	High risk	Low risk	Some concern	Low risk	High risk
Tokeranko et al, [125] 2021	Some concern	High risk	Low risk	Low risk	Some concern	High risk
Tseng et al, [126] 2013	Some concern	High risk	Low risk	Low risk	Low risk	High risk
Utter et al, [127] 1998	Some concern	Low risk	Low risk	Low risk	Low risk	Some concern
van Aggel-Leijssen et al, [128] 2021	Some concern	Low risk	Low risk	Some concern	Low risk	Some concern
Venojärvi et al, [129] 2013	High risk	Some concern	Low risk	Some concern	Low risk	High risk
Verity et al, [130] 1988	Some concern	High risk	Low risk	Some concern	Some concern	High risk
Villareal et al, [131] 2017	Some concern	Low risk	Low risk	Low risk	Low risk	Some concern
Wang et al, [132] 2017	Some concern	High risk	Low risk	High risk	Some concern	High risk
Womack et al, [133] 2000	Some concern	Low risk	High risk	Some concern	Low risk	High risk
Wood et al, [134] 1991	Some concern	High risk	Low risk	Low risk	Some concern	High risk
Wu et al, [135] 2017	Some concern	Low risk	Low risk	Low risk	Low risk	Some concern
You et al, [136] 2004	Some concern	Some concern	High risk	Low risk	Low risk	High risk
Zhang et al, [137] 2015	Some concern	Low risk	Low risk	Low risk	Low risk	Some concern
Zhang et al, [138] 2017	Some concern	Low risk	Low risk	Low risk	Low risk	Some concern
Zhang et al, [139] 2016	Low risk	Low risk	Low risk	Low risk	Low risk	Low risk
Zhao et al, [140] 2021	Some concern	High risk	Low risk	Some concern	Low risk	High risk
Donnelly et al, [141] 2013	Low risk	Low risk	Some concern	Low risk	Low risk	Some concern

[a] Numbers pertain to the reference numbers cited in the main text.

© 2024 Jayedi A et al. *JAMA Network Open*.

eTable 7. Subgroup analyses of the association of supervised aerobic exercise (each 30 min/week) with body weight (kg)

	No. of trials	Mean difference (95%CI)	I^2, $P_{heterogeneity}$	P group difference
All trials	109	-0.52 (-0.61, -0.44)	88%, <0.001	-
**Study risk of bias**				0.85
Low	13	-0.59 (-0.94, -0.25)	93%, <0.001	
Some concerns	46	-0.49 (-0.59, -0.39)	81%, <0.001	
High	50	-0.52 (-0.66, -0.38)		
**Geographical location**				0.70
North America	48	-0.48 (-0.57, -0.36)	73%, <0.001	
Europe	18	-0.57 (-0.81, -0.34)	88%, <0.001	
East Asia and Australia	26	-0.54 (-0.73, -0.36)	95%, <0.001	
South America	3	-0.31 (-0.69, -0.06)	0, 0.40	
Middel East	18	-0.60 (-0.91, -0.29)	81%, <0.001	
**Intervention characteristics**				
**Intensity**				0.14
Light	1	-0.33 (-0.46, -0.20)	-	
Light to moderate	7	-0.62 (-0.98, -0.24)	0, 0.98	
Moderate	53	-0.45(-0.54, -0.35)	72%, <0.001	
Moderate to vigorous	35	-0.60 (-0.81, -0.40)	95%, <0.001	
Vigorous	19	-0.56 (-0.78, -0.33)	87%, <0.001	
**Degree of adherence**				0.92
≥80%	45	-0.57 (-0.87, -0.27)	92%, <0.001	
<80%	8	-0.54 (-0.67, -0.41)	30%, 0.18	
Not reported	56	-0.51 (-0.64, -0.38)	84%, <0.001	
**Drop out**				0.85
<20%	81	-0.50 (-0.61, -0.39)	88%, <0.001	
≥20%	26	-0.62 (-0.80, -0.44)	87%, <0.001	
Not reported	2	-0.21 (-0.55, 0.13)	0, 0.59	
**Duration**				0.88
8 to ≤12 weeks	63	-0.51 (-0.62, -0.40)	77%, <0.001	
12 to ≤24 weeks	30	-0.56 (-0.76, -0.37)	94%, <0.001	
>24 weeks	16	-0.52 (-0.61, -0.44)	86%, <0.001	
**Type of exercise**				0.63
Walking or treadmill	64	-0.48 (-0.60, -0.37)	90%, <0.001	
Cycling	9	-0.53 (-0.83, -0.23)	46%, 0.06	
Mixed	36	-0.58 (-0.74, -0.42)	86%, <0.001	
**Frequency (per week)**				<0.001
1 to 2	2	-0.88 (-2.44, 0.68)	74%, 0.05	
3	63	-0.58 (-0.69, -0.47)	80%, <0.001	
3 to 5	9	-0.26 (-0.57, -0.04)	64%, 0.02	
4	13	-0.32 (-0.50, -0.15)	51%, 0.02	
5	19	-0.62 (-0.95, -0.29)	95%, <0.001	
6 to 7	3	-0.16 (-0.34, -0.01)	0%, 0.84	
**Progressive (frequency, intensity, or duration)**				0.02
Yes	73	-0.58 (-0.69, -0.46)	91%, <0.001	
No	36	-0.39 (-0.50, -0.28)	59%, <0.001	
**Calorie-restricted diet**				0.52
Yes	26	-0.57 (-0.74, -0.41)	88%, <0.001	

© 2024 Jayedi A et al. *JAMA Network Open*.

	No. of trials	Mean difference (95%CI)	I^2, $P_{heterogeneity}$	P group difference
No	83	-0.51 (-0.61, -0.40)	89%, <0.001	
**Participants characteristics**				
**Sex**				0.42
Women	44	-0.47 (-0.58, -0.37)	94%, <0.001	
Men	29	-0.49 (-0.70, -0.28)	77%, <0.001	
Mixed	36	-0.62 (-0.81, -0.44)	77%, <0.001	
**Weight status**				0.03
Overweight	3	-0.45 (-0.58, -0.32)	0%, 0.61	
Obese	36	-0.90 (-1.22, -0.58)	84%, <0.001	
Mixed	70	-0.47 (-0.59, -0.35)	90%, <0.001	
**Health status**				0.44
Otherwise healthy	71	-0.54 (-0.66, -0.43)	91%, <0.001	
With morbidity	38	-0.48 (-0.61, -0.35)	78%, <0.001	

© 2024 Jayedi A et al. *JAMA Network Open*.

eTable 8. Assessment of credibility of subgroup difference based on ICEMAN for body weight

Variable	Q1	Q2	Q3	Q4	Q5	Q6	Q7	Q8	Overall interpretation
Frequency	Completely between	Not applicable	Very small	Definitely no	Chance an unlikely explanation	Definitely no	Definitely yes	Probably no	Low
Progressive	Completely between	Not applicable	Large	Definitely no	Chance a likely explanation	Definitely no	Definitely yes	Not applicable	Low
Weight status	Completely between	Not applicable	Rather small	Definitely no	Chance a likely explanation	Definitely no	Definitely yes	Not applicable	Low

Q1, Is the analysis of effect modification based on comparison within rather than between trials? Q2, For within-trial comparisons, is the effect modification similar from trial to trial? Q3, For between-trial comparisons, is the number of trials large? Q4, Was the direction of the effect modification correctly hypothesized priori? Q5, Does a test for interaction suggest that chance is an unlikely explanation of the apparent effect modification? Q6, Did the authors test only a small number of effect modifiers? Q7, Did the authors use a random effects model? Q8, If the effect modifier is a continuous variable, were arbitrary cut points avoided?

© 2024 Jayedi A et al. *JAMA Network Open*.

eTable 9. Subgroup analyses of the association of supervised aerobic exercise (each 30 min/week) with waist circumference (cm)

	No. of trials	Mean difference (95%CI)	I^2, $P_{heterogeneity}$	P group difference
All trials	62	-0.56 (-0.67, -0.45)	88%, <0.001	-
**Study risk of bias**				0.10
Low	13	-0.39 (-0.54, -0.25)	79%, <0.001	
Some concerns	23	-0.67 (-0.88, -0.45)	81%, <0.001	
High	26	-0.55 (-0.72, -0.37)	78%, <0.001	
**Geographical location**				0.41
North America	21	-0.44 (-.50, -0.30)	72%, <0.001	
Europe	10	-0.56 (-0.93, -0.19)	91%< 0.001	
East Asia and Australia	17	-0.72 (-0.96, -0.48)	90%, <0.001	
South America	3	-0.46 (-0.86, -0.07)	0, 0.66	
Middel East	11	-0.49 (-0.73, -0.25)	83%, <0.001	
**Intervention characteristics**				
**Intensity**				0.01
Light	1	-0.95 (-1.17, -0.74)	-	
Light to moderate	4	-0.37 (-0.62, -0.12)	0, 0.74	
Moderate	33	-0.58 (-0.75, -0.41)	89%, <0.001	
Moderate to vigorous	22	-0.61 (-0.79, -0.43)	81%, <0.001	
Vigorous	7	-0.62 (-1.23, -0.04)	94%, <0.001	
**Degree of adherence**				0.80
≥80%	30	-0.60 (-0.81, -0.38)	88%, <0.001	
<80%	7	-0.80 (-1.17, -0.42)	52%, 0.10	
Not reported	25	-0.62 (-0.85, -0.38)	81%, <0.001	
**Drop out**				0.57
<20%	40	-0.52 (-0.65, -0.39)	83%, <0.001	
≥20%	19	-0.64 (-0.85, -0.43)	81%, <0.001	
Not reported	3	-0.43 (-0.90, 0.02)	51%, 0.13	
**Duration**				0.61
8 to ≤12 weeks	34	-0.61 (-0.78, -0.44)	82%, <0.001	
12 to ≤24 weeks	19	-0.50 (-0.66, -0.33)	78%, <0.001	
>24 weeks	9	-0.51 (-0.82, -0.19)	89%, <0.001	
**Type of exercise**				0.80
Walking or treadmill	42	-0.53 (-0.66, -0.39)	81%, <0.001	
Cycling	2	-0.66 (-1.36, 0.04)	92%, <0.001	
Mixed	18	-0.62 (-0.92, -0.33)	88%, <0.001	
**Frequency (per week)**				0.29
1 to 2	2	-0.92 (-2.47, 0.63)	0, 0.60	
3	33	-0.81 (-1.20, -0.42)	62%, 0.02	
3 to 5	6	-0.59 (-0.75, -0.42)	87%, <0.001	
4	4	-0.61 (-0.95, -0.27)	59%, 0.06	
5	15	-0.39 (-0.54, -0.24)	64%, <0.001	
6 to 7	2	-0.50 (-1.56, -0.57)	90%, 0.001	
**Progressive (frequency, intensity, or duration)**				0.72
Yes	44	-0.63 (-0.81, -0.44)	87%, <0.001	
No	18	-0.58 (-0.76, -0.40)	61%, 0.001	
**Calorie-restricted diet**				0.54
Yes	11	-0.49 (-0.74, -0.24)	83%, 0<0.001	

© 2024 Jayedi A et al. *JAMA Network Open*.

	No. of trials	Mean difference (95%CI)	I^2, $P_{heterogeneity}$	P group difference
No	51	-0.57 (-0.70, -0.45)	85%, 0<0.001	
**Participants characteristics**				
**Sex**				0.90
Women	23	-0.53 (-0.67, -0.39)	56%, 0.01	
Men	15	-0.60 (-0.88, -0.33)	88%, <0.001	
Mixed	24	-0.54 (-0.71, -0.36)	88%, <0.001	
**Weight status**				
Overweight	1	-0.42 (-0.96, 0.13)	-	0.47
Obese	24	-0.61 (-0.76, -0.45)	78%, <0.001	
Mixed	37	-0.47 (-0.63, -0.32)	86%, <0.001	
**Health status**				0.54
Otherwise healthy	36	-0.61 (-0.78, -0.43)	89%, <0.001	
With morbidity	26	-0.50 (-0.64, -0.37)	71%, <0.001	

© 2024 Jayedi A et al. *JAMA Network Open*.

eTable 10. Assessment of credibility of subgroup difference based on ICEMAN for waist circumference

Variable	Q1	Q2	Q3	Q4	Q5	Q6	Q7	Q8	Overall interpretation
Intensity	Completely between	Not applicable	Very small	Definitely yes	Chance a likely explanation	Definitely yes	Definitely yes	Not applicable	Low

Q1, Is the analysis of effect modification based on comparison within rather than between trials? Q2, For within-trial comparisons, is the effect modification similar from trial to trial? Q3, For between-trial comparisons, is the number of trials large? Q4, Was the direction of the effect modification correctly hypothesized priori? Q5, Does a test for interaction suggest that chance is an unlikely explanation of the apparent effect modification? Q6, Did the authors test only a small number of effect modifiers? Q7, Did the authors use a random effects model? Q8, If the effect modifier is a continuous variable, were arbitrary cut points avoided?

© 2024 Jayedi A et al. *JAMA Network Open*.

eTable 11. Subgroup analyses of the association of supervised aerobic exercise (each 30 min/week) with body fat percentage (%)

	No. of trials	Mean difference (95%CI)	I^2, $P_{heterogeneity}$	P group difference
All trials	65	-0.37 (-0.43, -0.31)	83%, <0.001	-
**Study risk of bias**				0.94
Low	8	-0.36 (-0.50, -0.22)	0, 0.77	
Some concerns	27	-0.37 (-0.45, -0.28)	82%, <0.001	
High	30	-0.39 (-0.49, -0.28)	87%, <0.001	
**Geographical location**				0.28
North America	24	0.37 (-0.46, -0.25)	80%, <0.001	
Europe	7	-0.25 (-0.39, -0.11)	87%, <0.001	
East Asia and Australia	17	-0.43 (-0.55, -0.31)	75%, <0.001	
South America	2	-0.24 (-0.46, -0.02)	0, 0.79	
Middel East	15	-0.41 (-0.57, -0.25)	81%, <0.001	
**Intervention characteristics**				
**Intensity**				<0.001
Light	1	-0.22 (-0.47, -0.17)	-	
Light to moderate	3	-0.11 (-0.22, -0.01)	0, 0.58	
Moderate	32	-0.30 (-0.39, -0.22)	83%, <0.001	
Moderate to vigorous	20	-0.37 (-0.47, -0.28)	83%, <0.001	
Vigorous	12	-0.59 (-0.76, -0.42)	73%, <0.001	
**Degree of adherence**				0.10
≥80%	29	-0.32 (-0.42, -0.22)	81%, <0.001	
<80%	4	-0.26 (-0.45, -0.07)	86%, <0.001	
Not reported	32	-0.44 (-0.54, -0.35)	79%, <0.001	
**Drop out**				0.08
<20%	47	-0.40 (-0.47, -0.32)	81%, <0.001	
≥20%	15	-0.34 (-0.46, -0.22)	74%, <0.001	
Not reported	3	-0.14 (-0.36, 0.08)	46%, 0.16	
**Duration**				0.97
8 to ≤12 weeks	41	-0.37 (-0.45, -0.29)	77%, <0.001	
12 to ≤24 weeks	14	-0.37 (-0.56, -0.18)	84%, <0.001	
>24 weeks	10	-0.39 (-0.50, -0.27)	84%, <0.001	
**Type of exercise**				0.02
Walking or treadmill	7	-0.25 (-0.32, -0.17)	14%, 0.20	
Cycling	38	-0.37 (-0.46, -0.29)	78%, <0.001	
Mixed	20	-0.40 (-0.54, -0.27)	87%, <0.001	
**Frequency (per week)**				<0.001
1 to 2	2	-0.16 (-0.70, 0.38)	0, 0.87	
3	37	-0.40 (-0.52, -0.29)	85%, <0.001	
3 to 5	5	-0.45 (-0.60, -0.30)	34%, 0.20	
4	10	-0.36 (-0.48, -0.25)	86%, <0.001	
5	10	-0.22 (-0.30, -0.13)	53%, 0.02	
6 to 7	1	-1.17 (-1.54, -0.80)	-	
**Progressive (frequency, intensity, or duration)**				0.34
Yes	47	-0.36 (-0.43, -0.28)	83%, <0.001	
No	18	-0.43 (-0.55, -0.30)	83%, <0.001	
**Calorie-restricted diet**				0.76
Yes	14	-0.35 (-0.53, -0.16)	79%, <0.001	

© 2024 Jayedi A et al. *JAMA Network Open*.

	No. of trials	Mean difference (95%CI)	I^2, $P_{heterogeneity}$	P group difference
No	51	-0.38 (-0.45, -0.31)	82%, <0.001	
**Participants characteristics**				
**Sex**				0.02
Women	29	-0.30 (-0.39, -0.21)	75%, <0.001	
Men	21	-0.52 (-0.66, -0.39)	82%, <0.001	
Mixed	15	-0.33 (-0.44, -0.22)	85%, <0.001	
**Weight status**				0.33
Overweight	3	-0.41 (-0.55, -0.26)	80%, 0.007	
Obese	20	-0.58 (-1.09, -0.07)	89%, <0.001	
Mixed	42	-0.34 (-0.41, -0.27)	78%, <0.001	
**Health status**				0.20
Otherwise healthy	46	-0.40 (-0.48, -0.32)	85%, <0.001	
With morbidity	19	-0.31 (-0.42, -0.21)	77%, <0.001	

© 2024 Jayedi A et al. *JAMA Network Open*.

eTable 12. Assessment of credibility of subgroup difference based on ICEMAN for body fat percentage

Variable	Q1	Q2	Q3	Q4	Q5	Q6	Q7	Q8	Overall interpretation
Intensity	Completely between	Not applicable	Very small	Definitely yes	Chance an unlikely explanation	Definitely yes	Definitely yes	Not applicable	Low
Drop out	Completely between	Not applicable	Large	Definitely no	Chance a very likely explanation	Definitely no	Definitely yes	Definitely yes	Low
Type of exercise	Completely between	Not applicable	Rather large	Definitely no	Chance a likely explanation	Definitely no	Definitely yes	Definitely yes	Low
Frequency	Completely between	Not applicable	Very small	Definitely no	Chance an unlikely explanation	Definitely no	Definitely yes	Not applicable	Low
Sex	Completely between	Not applicable	Large	Definitely no	Chance a likely explanation	Definitely no	Definitely yes	Not applicable	Low

Q1, Is the analysis of effect modification based on comparison within rather than between trials? Q2, For within-trial comparisons, is the effect modification similar from trial to trial? Q3, For between-trial comparisons, is the number of trials large? Q4, Was the direction of the effect modification correctly hypothesized priori? Q5, Does a test for interaction suggest that chance is an unlikely explanation of the apparent effect modification? Q6, Did the authors test only a small number of effect modifiers? Q7, Did the authors use a random effects model? Q8, If the effect modifier is a continuous variable, were arbitrary cut points avoided?

eTable 13. Subgroup analyses of the association of supervised aerobic exercise (each 30 min/week) with body fat mass (kg)

	No. of trials	Mean difference (95%CI)	I^2, $P_{heterogeneity}$	P group difference
All trials	50	-0.43 (-0.53, -0.33)	85%, <0.001	-
**Study risk of bias**				0.007
Low	7	-0.20 (-0.32, -0.08)	26%, 0.23	
Some concerns	23	-0.37 (-0.51, -0.24)	80%, <0.001	
High	20	-0.55 (-0.75, -0.36)	87%, <0.001	
**Geographical location**				0.09
North America	26	-0.43 (-0.58, -0.29)	87%, <0.001	
Europe	4	-0.51 (-0.76, -0.27)	45%, 0.14	
East Asia and Australia	12	-0.42 (-0.62, -0.22)	74%, <0.001	
South America	3	-0.42 (-0.72, -0.11)	58%, 0.09	
Middel East	5	-0.35 (-0.85, 0.15)	92%, <0.001	
**Intervention characteristics**				
**Intensity**				0.62
Light	1	-0.51 (-0.72, -0.31)	-	
Light to moderate	1	-0.21 (-0.55, -0.13)	-	
Moderate	27	-0.41 (-0.53, -0.28)	77%, <0.001	
Moderate to vigorous	13	-0.46 (-0.65, -0.27)	88%, <0.001	
Vigorous	11	-0.44 (-0.54, -0.34)	93%, <0.001	
**Degree of adherence**				0.07
≥80%	27	-0.33 (-0.45, -0.22)	70%, <0.001	
<80%	2	-0.24 (-0.48, -0.01)	81%, <0.001	
Not reported	21	-0.55 (-0.72, -0.37)	89%, <0.001	
**Drop out**				0.06
<20%	36	-0.48 (-0.62, -0.34)	87%, <0.001	
≥20%	13	-0.27 (-0.36, -0.18)	41%, 0.06	
Not reported	1	-0.13 (-0.68, 0.42)	-	
**Duration**				0.43
8 to ≤12 weeks	27	-0.38 (-0.51, -0.25)	83%, <0.001	
12 to ≤24 weeks	13	-0.54 (-0.75, -0.33)	80%, <0.001	
>24 weeks	10	-0.40 (-0.66, -0.14)	90%, <0.001	
**Type of exercise**				0.34
Walking or treadmill	31	-0.40 (-0.53, -0.27)	84%, <0.001	
Cycling	6	-0.31 (-0.49, -0.14)	0, 0.96	
Mixed	13	-0.51 (-0.71, -0.31)	89%, <0.001	
**Frequency (per week)**				<0.001
1 to 2	2	-0.49 (-0.87, -0.11)	47%, 0.17	
3	1	-0.70 (-1.08, -0.32)	-	
3 to 5	2	-0.77 (-1.30, -0.24)	68%, 0.08	
4	28	-0.47 (-0.62, -0.32)	88%, <0.001	
5	6	-0.29 (-0.41, -0.18)	0, 0.51	
6 to 7	11	-0.17 (-0.24, -0.11)	0, 0.45	
**Progressive (frequency, intensity, or duration)**				0.67
Yes	38	-0.44 (-0.56, -0.32)	86%, <0.001	
No	12	-0.41 (-0.61, -0.20)	81%, <0.001	
**Calorie-restricted diet**				0.06
Yes	15	-0.58 (-0.79, -0.37)	84%, <0.001	

© 2024 Jayedi A et al. *JAMA Network Open*.

	No. of trials	Mean difference (95%CI)	I^2, $P_{heterogeneity}$	P group difference
No	35	-0.36 (-0.47, -0.25)	80%, <0.001	
**Participants characteristics**				
**Sex**				0.38
Women	22	-0.50 (-0.66, -0.34)	82%, <0.001	
Men	13	-0.32 (-0.52, -0.13)	79%, <0.001	
Mixed	15	-0.41 (-0.60, -0.22)	89%, <0.001	
**Weight status**				0.99
Overweight	1	-0.42 (-0.96, 0.13)	-	
Obese	19	-0.44 (-0.61, -0.26)	89%, <0.001	
Mixed	30	-0.43 (-0.56, -0.29)	82%, <0.001	
**Health status**				0.70
Otherwise healthy	39	-0.44 (-0.56, -0.32)	87%, <0.001	
With morbidity	11	-0.40 (-0.59, -0.21)	66%, 0.001	

© 2024 Jayedi A et al. *JAMA Network Open*.

eTable 14. Assessment of credibility of subgroup difference based on ICEMAN for body fat mass

Variable	Q1	Q2	Q3	Q4	Q5	Q6	Q7	Q8	Overall interpretation
Study risk of bias	Completely between	Not applicable	Rather large	Definitely yes	Chance an unlikely explanation	Definitely yes	Definitely yes	Not applicable	Moderate
Degree of adherence	Completely between	Not applicable	Very small	Definitely no	Chance a very likely explanation	Definitely no	Definitely yes	Definitely yes	Low
Dropout	Completely between	Not applicable	Very small	Definitely no	Chance a very likely explanation	Definitely no	Definitely yes	Definitely yes	Low
Frequency	Completely between	Not applicable	Very small	Definitely no	Chance an unlikely explanation	Definitely no	Definitely yes	Not applicable	Low
Calorie-restricted diet	Completely between	Not applicable	Large	Definitely no	Chance a very likely explanation	Definitely no	Definitely yes	Not applicable	Low

Q1, Is the analysis of effect modification based on comparison within rather than between trials? Q2, For within-trial comparisons, is the effect modification similar from trial to trial? Q3, For between-trial comparisons, is the number of trials large? Q4, Was the direction of the effect modification correctly hypothesized priori? Q5, Does a test for interaction suggest that chance is an unlikely explanation of the apparent effect modification? Q6, Did the authors test only a small number of effect modifiers? Q7, Did the authors use a random effects model? Q8, If the effect modifier is a continuous variable, were arbitrary cut points avoided?

© 2024 Jayedi A et al. *JAMA Network Open*.

eTable 15. Subgroup analyses of the association of supervised aerobic exercise (each 30 min/week) with visceral adipose tissue (cm²)

	No. of trials	Mean difference (95%CI)	I^2, $P_{heterogeneity}$	P group difference
All trials	26	-1.60 (-2.12, -1.07)	66%, <0.001	-
**Study risk of bias**				0.46
Low	5	-1.31 (-2.06, -0.55)	0, 0.52	
Some concerns	13	-1.28 (-1.87, -0.69)	51%, 0.01	
High	8	-2.15 (-3.41, -0.89)	81%, <0.001	
**Geographical location**				0.33
North America	13	-1.39 (-2.18, -0.60)	77%, <0.001	
Europe	1	-0.92 (-2.47, 0.63)	-	
East Asia and Australia	11	-2.00 (-2.71, -1.29)	39%, 0.09	
South America	0	-	-	
Middel East	1	-1.60 (-2.12, -1.07)	-	
**Intervention characteristics**				
**Intensity**				0.03
Light	0	-	-	
Light to moderate	0	-	-	
Moderate	19	-1.54 (-2.27, -0.81)	70%, <0.001	
Moderate to vigorous	5	-1.03 (-2.06, 0.01)	66%, 0.02	
Vigorous	8	-2.29 (-3.50, -1.09)	82%, <0.001	
**Degree of adherence**				0.36
≥80%	14	-1.51 (-2.09, -0.93)	61%, 0.002	
<80%	1	-2.67 (-4.15, -1.19)	-	
Not reported	11	-1.55 (-2.66, -0.44)	71%, <0.001	
**Drop out**				<0.001
<20%	19	-1.47 (-1.98, -0.96)	52%, 0.004	
≥20%	6	-0.96 (-1.58, -0.34)	0, 0.72	
Not reported	1	-5.11 (-6.39, -3.83)	-	
**Duration**				0.004
8 to ≤12 weeks	15	-1.99 (-2.81, -1.18)	74%, <0.001	
12 to ≤24 weeks	6	-0.54 (-1.13, 0.05)	2%, 0.59	
>24 weeks	5	-1.79 (-2.49, -1.10)	23%, 0.26	
**Type of exercise**				0.02
Walking or treadmill	16	-1.66 (-2.19, -1.13)	31%, 0.10	
Cycling	5	-2.37 (-4.09, -0.64)	91%, <0.001	
Mixed	5	-0.59 (-1.23, 0.04)	7%, 0.42	
**Frequency (per week)**				0.03
1 to 2	0	-	-	
3	11	-2.06 (-2.72, -1.40)	25%, 0.20	
3 to 5	0	-	-	
4	5	-1.92 (-3.60, -0.24)	91%, <0.001	
5	10	-0.91 (-1.47, -0.34)	27%, 0.26	
6 to 7	-			
**Progressive (frequency, intensity, or duration)**				0.67
Yes	20	-1.46 (-1.94, -0.99)	51%, 0.006	
No	6	-1.87 (-3.67, -0.08)	82%, <0.001	
**Calorie-restricted diet**				<0.001
Yes	7	-0.60 (-1.04, -0.16)	0, 0.75	

© 2024 Jayedi A et al. *JAMA Network Open*.

	No. of trials	Mean difference (95%CI)	I^2, $P_{heterogeneity}$	P group difference
No	19	-2.00 (-2.14, -1.06)	64%, <0.001	
**Participants characteristics**				
**Sex**				0.96
Women	12	-1.52 (-2.14, -0.90)	29%, 0.13	
Men	5	-1.46 (-2.77, -0.15)	73%, 0.006	
Mixed	9	-1.69 (-2.78, -0.60)	83%, <0.001	
**Weight status**				<0.001
Overweight	0	-	-	
Obese	6	-1.88 (-2.48, -1.28)	70%, <0.001	
Mixed	20	-0.49 (-1.25, 0.29)	12%, 0.61	
**Health status**				0.93
Otherwise healthy	17	-1.55 (-2.33, -0.77)	74%, <0.001	
With morbidity	9	-1.59 (-2.21, -0.98)	39%, 0.24	

© 2024 Jayedi A et al. *JAMA Network Open*.

eTable 16. Assessment of credibility of subgroup difference based on ICEMAN for visceral adipose tissue

Variable	Q1	Q2	Q3	Q4	Q5	Q6	Q7	Q8	Overall interpretation
Intensity	Completely between	Not applicable	Rather large	Definitely yes	Chance a likely explanation	Definitely yes	Definitely yes	Not applicable	Low
Drop out	Completely between	Not applicable	Very small	Definitely no	Chance an unlikely explanation	Definitely no	Definitely yes	Definitely yes	Low
Duration	Completely between	Not applicable	Very small	Definitely no	Chance an unlikely explanation	Definitely no	Definitely yes	Definitely yes	Low
Type of exercise	Completely between	Not applicable	Very small	Definitely no	Chance a likely explanation	Definitely no	Definitely yes	Not applicable	Low
Frequency	Completely between	Not applicable	Large	Definitely no	Chance a likely explanation	Definitely no	Definitely yes	Not applicable	Low
Calorie-restricted diet	Completely between	Not applicable	Large	Definitely no	Chance an unlikely explanation	Definitely no	Definitely yes	Not applicable	Low
Weight status	Completely between	Not applicable	Large	Definitely no	Chance an unlikely explanation	Definitely no	Definitely yes	Not applicable	Low

Q1, Is the analysis of effect modification based on comparison within rather than between trials? Q2, For within-trial comparisons, is the effect modification similar from trial to trial? Q3, For between-trial comparisons, is the number of trials large? Q4, Was the direction of the effect modification correctly hypothesized priori? Q5, Does a test for interaction suggest that chance is an unlikely explanation of the apparent effect modification? Q6, Did the authors test only a small number of effect modifiers? Q7, Did the authors use a random effects model? Q8, If the effect modifier is a continuous variable, were arbitrary cut points avoided?

eTable 17. Subgroup analyses of the association of supervised aerobic exercise (each 30 min/week) with subcutaneous adipose tissue (cm²)

	No. of trials	Mean difference (95%CI)	I^2, $P_{heterogeneity}$	P group difference
All trials	27	-1.37 (-1.82, -0.92)	74%, <0.001	-
**Study risk of bias**				0.42
Low	5	-1.70 (-2.89, 0.01)	40%, 0.15	
Some concerns	15	-1.16 (-1.67, -0.65)	77%, <0.001	
High	7	-1.92 (-3.12, -0.72)	30%, 0.20	
**Geographical location**				0.51
North America	15	-1.30 (-1.89, -0.71)	70%, <0.001	
Europe	-	-	-	
East Asia and Australia	10	-1.83 (-2.88, -0.77)	78%, <0.001	
South America	-	-	-	
Middel East	2	-1.07 (-1.79, -0.36)	0, 0.55	
**Intervention characteristics**				
**Intensity**				0.15
Light	0	-	-	
Light to moderate	0	-	-	
Moderate	16	-2.12 (-3.02, -1.21)	77%, <0.001	
Moderate to vigorous	7	-0.99 (-1.75, -0.22)	83%, <0.001	
Vigorous	8	-1.74 (-2.44, -1.04)	32%, 0.17	
**Degree of adherence**				0.13
≥80%	15	-1.02 (-1.53, -0.51)	63%, 0.01	
<80%	1	-1.70 (-2.47, -0.93)	-	
Not reported	11	-2.10 (-3.22, -0.98)	77%, <0.001	
**Drop out**				0.50
<20%	18	-1.22 (-1.74, -0.70)	62%, <0.001	
≥20%	8	-1.59 (-2.66,-0.52)	84%, <0.001	
Not reported	1	-2.42 (-4.56, -0.29)	-	
**Duration**				0.02
8 to ≤12 weeks	15	-1.87 (-2.71, -1.02)	74%, <0.001	
12 to ≤24 weeks	7	-0.69 (-1.21, -0.17)	68%, 0.005	
>24 weeks	5	-1.66 (-2.39, -0.93)	0, 0.43	
**Type of exercise**				0.07
Walking or treadmill	18	-0.95 (-1.42, -0.48)	62%, <0.001	
Cycling	4	-2.33 (-3.47, -1.20)	21%, 0.28	
Mixed	5	-1.47 (-2.20, -0.74)	40%, 0.15	
**Frequency (per week)**				0.59
1 to 2	0	-	-	
3	12	-1.77 (-2.62, -0.91)	72%, <0.001	
3 to 5	0	-	-	
4	5	-1.58 (-2.89, -0.27)	81%, <0.001	
5	10	-1.16 (-1.98, -0.33)	67%, 0.001	
6 to 7	0	-		
**Progressive (frequency, intensity, or duration)**				0.63
Yes	21	-1.43 (-1.95, -0.90)	73%, <0.001	
No	6	-1.85 (-3.53, -0.18)	57%, 0.01	
**Calorie-restricted diet**				0.43
Yes	9	-1.20 (-1.94, -0.45)	71%, <0.001	

© 2024 Jayedi A et al. *JAMA Network Open*.

	No. of trials	Mean difference (95%CI)	I^2, $P_{heterogeneity}$	P group difference
No	18	-1.59 (-2.22, -0.96)	73%, <0.001	
**Participants characteristics**				
**Sex**				0.02
Women	14	-1.49 (-2.19, -0.78)	67%, <0.001	
Men	6	-0.45 (-1.09, 0.19)	61%, 0.02	
Mixed	7	-1.70 (-2.43, -0.97)	30%, 0.20	
**Weight status**				0.02
Overweight	-	-	-	
Obese	7	-1.87 (-2.57, -1.17)	69%, <0.001	
Mixed	20	-0.79 (-1.37, -0.22)	73%, 0.001	
**Health status**				0.40
Otherwise healthy	18	-1.37 (-1.92, -0.82)	76%, <0.001	
With morbidity	9	-1.97 (-3.25, -0.69)	60%, 0.01	

© 2024 Jayedi A et al. *JAMA Network Open*.

eTable 18. Assessment of credibility of subgroup difference based on ICEMAN for subcutaneous adipose tissue.

Variable	Q1	Q2	Q3	Q4	Q5	Q6	Q7	Q8	Overall interpretation
Duration	Completely between	Not applicable	Rather large	Definitely no	Chance a likely explanation	Definitely no	Definitely yes	Not applicable	Low
Type of exercise	Completely between	Not applicable	Rather large	Definitely no	Chance a very likely explanation	Definitely no	Definitely yes	Not applicable	Low
Sex	Completely between	Not applicable	Rather large	Definitely no	Chance a likely explanation	Definitely no	Definitely yes	Not applicable	Low
Weight status	Completely between	Not applicable	Rather large	Definitely no	Chance a likely explanation	Definitely no	Definitely yes	Not applicable	Low

Q1, Is the analysis of effect modification based on comparison within rather than between trials? Q2, For within-trial comparisons, is the effect modification similar from trial to trial? Q3, For between-trial comparisons, is the number of trials large? Q4, Was the direction of the effect modification correctly hypothesized priori? Q5, Does a test for interaction suggest that chance is an unlikely explanation of the apparent effect modification? Q6, Did the authors test only a small number of effect modifiers? Q7, Did the authors use a random effects model? Q8, If the effect modifier is a continuous variable, were arbitrary cut points avoided?

© 2024 Jayedi A et al. *JAMA Network Open*.

eTable 19. The association of different doses of aerobic exercise with measures of body weight, waist and fat in adults with overweight or obesity (mean difference and 95%CI)

Aerobic exercise (min/week)	0 (Ref)	50	100	150	200	250	300
**Body weight (kg)**							
All trials (n=109)	0	-1.26 (-1.82, -0.70)	-2.16 (-2.80, -1.52)	-2.79 (-3.29, -2.29)	-3.28 (-3.97, -2.59)	-3.74 (-4.94, -2.54)	-4.19 (-5.98, -2.41)
Moderate (n=53)	0	-0.88 (-1.45, -0.30)	-1.64 (-2.32, -0.95)	-2.31 (-2.87, -1.74)	-2.92 (-3.68, -2.17)	-3.52 (-4.84, -2.21)	-4.12 (-6.08, -2.17)
Moderate to vigorous (n=35)	0	-1.97 (-2.84, -1.11)	-3.08 (-4.06, -2.10)	-3.47 (-4.34, -2.61)	-3.47 (-5.02, -1.92)	-3.38 (-6.03, -0.72)	-3.28 (-7.12, 0.56)
Vigorous (n=19)	0	-1.25 (-3.93, 0.89)	-2.17 (-4.45, 0.10)	-2.87 (-4.25, -1.49)	-3.47 (-5.54, -1.40)	-4.07 (-7.98, -0.16)	-
**Waist circumference (cm)**							
All trials (n=62)	0	-1.60 (-2.24, -0.96)	-2.65 (-3.43, -1.86)	-3.26 (-3.91, -2.61)	-3.61 (-4.39, -2.83)	-3.87 (-5.19, -2.54)	-4.12 (-6.10, -2.14)
Moderate (n=33)	0	-1.47 (-2.12, -0.82)	-2.48 (-3.24, -1.72)	-3.13 (-3.80, -2.46)	-3.55 (-4.58, -2.51)	-3.88 (-5.68, -2.09)	-4.21 (-6.85, -1.58)
Moderate to vigorous (n=22)	0	-0.84 (-2.31, 0.64)	-1.71 (-3.45, 0.04)	-2.06 (-3.74, -1.47)	-3.51 (-4.60, -2.43)	-4.43 (-6.72, -2.13)	-5.34 (-9.05, -1.63)
Vigorous (n=7)	-	-	-	-	-	-	-
**Body fat percentage (%)**							
All trials (n=65)	0	-1.29 (-1.76, -0.82)	-1.92 (-2.46, -1.39)	-2.08 (-2.47, -1.69)	-2.01 (-2.51, -1.50)	-1.89 (-2.79, -1.00)	-1.78 (-3.12, -0.44)
Moderate (n=32)	0	-1.09 (-1.68, -0.51)	-1.61 (-2.31, -0.91)	-1.70 (-2.24, -1.17)	-1.59 (-2.12, -1.06)	-1.43 (-2.31, -0.54)	-1.27 (-2.62, 0.08)
Moderate to vigorous (n=20)	0	-1.32 (-2.79, -0.84)	-2.00 (-2.51, -1.48)	-2.16 (-2.70, -1.61)	-2.06 (-3.13, -1.00)	-1.92 (-3.68, -0.17)	-
Vigorous (n=12)	0	-1.85 (-3.42, -0.28)	-2.84 (-4.62, -1.05)	-3.21 (-4.36, -2.07)	-3.35 (-4.26, -2.45)	-3.48 (-5.26, -1.70)	-

© 2024 Jayedi A et al. *JAMA Network Open*.

Aerobic exercise (min/week)	0 (Ref)	50	100	150	200	250	300
**Body fat mass (kg)**							
All trials (n=50)	0	-1.27 (-1.87, -0.68)	-2.03 (-2.77, -1.29)	-2.40 (-3.00, -1.80)	-2.56 (-3.13, -2.00)	-2.68 (-3.56, -1.80)	-2.79 (-4.12, -1.46)
Moderate (n=27)	0	-0.67 (-1.31, -0.03)	-1.35 (-2.11, -0.58)	-2.03 (-2.65, -1.41)	-2.72 (-3.57, -1.87)	-3.41 (-4.91, -1.92)	-4.11 (-6.34, -1.87)
Moderate to vigorous (n=13)	0	-1.45 (-2.44, -0.45)	-2.23 (-3.36, -1.10)	-2.51 (-3.42, -1.60)	-2.60 (-3.92, -1.28)	-2.68 (-4.84, -0.52)	-2.75 (-5.86, 0.35)
Vigorous (n=11)	-	-	-	-	-	-	-
**Visceral adipose tissue (cm^2)**							
All trials (n=26)	0	-6.33 (-9.79, -2.86)	-9.68 (-12.24, -5.11)	-10.36 (-14.10, -6.62)	-9.43 (-12.25, -6.62)	-7.96 (-12.17, -3.74)	-6.45 (-13.30, 0.39)
**Subcutaneous adipose tissue (cm^2)**							
All trials (n=27)	0	-4.22 (-6.18, -2.27)	-7.21 (-9.48, -4.93)	-9.11 (-11.33, -6.89)	-10.37 (-14.33, -6.40)	-11.40 (-18.16, -4.56)	-12.43 (-22.17, -2.69)

© 2024 Jayedi A et al. *JAMA Network Open*.

eTable 20. GRADE evidence for the association of aerobic exercise with measures of body weight, waist and fat

Certainty assessment							№ of patients		Effect		Certainty	Importance
№ of studies	Study design	Risk of bias	Inconsistency	Indirectness	Imprecision	Other considerations	[intervention]	[comparison]	Relative (95% CI)	Absolute (95% CI)		
**Body weight (kg)**												
109	randomised trials	serious[a]	not serious[b]	not serious	serious[c]	dose response gradient	3125	3173	-	MD **0.52 kg lower** (0.61 lower to 0.44 lower)	⨁⨁⨁◯ Moderate	CRITICAL
**Waist circumference (cm)**												
62	randomised trials	serious[a]	not serious[d]	not serious	not serious[e]	dose response gradient	2119	2162	-	MD **0.56 cm lower** (0.67 lower to 0.45 lower)	⨁⨁⨁⨁ High	IMPORTANT
**Body fat percentage (%)**												

© 2024 Jayedi A et al. *JAMA Network Open*.

Certainty assessment							№ of patients		Effect		Certainty	Importance
№ of studies	Study design	Risk of bias	Inconsistency	Indirectness	Imprecision	Other considerations	[intervention]	[comparison]	Relative (95% CI)	Absolute (95% CI)		
65	randomised trials	serious[a]	not serious[f]	not serious	not serious[g]	publication bias strongly suspected dose response gradient	1728	1738	-	MD **0.37 % lower** (0.43 lower to 0.31 lower)	⨁⨁⨁◯ Moderate	IMPORTANT
**Body fat mass (kg)**												
50	randomised trials	serious[h]	not serious[i]	not serious	not serious[j]	dose response gradient	1451	1476	-	MD **0.43 kg lower** (0.53 lower to 0.33 lower)	⨁⨁⨁⨁ High	IMPORTANT
**Visceral adipose tissue (cm^2)**												
26	randomised trials	serious[a]	not serious[k]	not serious	not serious[l]	dose response gradient	741	760	-	MD **1.60 cm^2 lower** (2.12 lower to 1.07 lower)	⨁⨁⨁⨁ High	IMPORTANT

© 2024 Jayedi A et al. *JAMA Network Open*.

Certainty assessment							№ of patients		Effect		Certainty	Importance
№ of studies	Study design	Risk of bias	Inconsistency	Indirectness	Imprecision	Other considerations	[intervention]	[comparison]	Relative (95% CI)	Absolute (95% CI)		
**Subcutaneous adipose tissue (cm^2)**												
27	randomised trials	serious[a]	not serious[m]	not serious	not serious[n]	publication bias strongly suspected dose response gradient	805	829	-	MD **1.37 cm^2 lower** (1.82 lower to 0.92 lower)	⊕⊕⊕◯ Moderate	IMPORTANT
**Adverse events**												
10	randomised trials	serious[o]	not serious	not serious	serious[p]	none	36/757 (4.8%)	11/413 (2.7%)	**RR 1.74** (1.02 to 2.97)	**20 more per 1,000** (from 10 more to 20 more)	⊕⊕◯◯ Low	CRITICAL
**Hypoglycemic reactions**												

© 2024 Jayedi A et al. *JAMA Network Open*.

Certainty assessment							№ of patients		Effect		Certainty	Importance
№ of studies	Study design	Risk of bias	Inconsistency	Indirectness	Imprecision	Other considerations	[intervention]	[comparison]	Relative (95% CI)	Absolute (95% CI)		
3	randomised trials	serious[o]	not serious	not serious	very serious[q]	none	4/168 (2.4%)	1/167 (0.6%)	**RR 2.51** (0.49 to 12.78)	**10 more per 1,000** (from 10 fewer to 30 more)	⨁◯◯◯ Very low	CRITICAL
**Medication reduction**												
2	randomised trials	serious[o]	not serious	not serious	serious[r]	none	18/422 (4.3%)	6/165 (3.6%)	**RR 1.43** (0.57 to 3.60)	**10 more per 1,000** (from 20 fewer to 50 more)	⨁⨁◯◯ Low	CRITICAL
**Health-related quality of life (mental score)**												

© 2024 Jayedi A et al. *JAMA Network Open*.

Certainty assessment							№ of patients		Effect		Certainty	Importance
№ of studies	Study design	Risk of bias	Inconsistency	Indirectness	Imprecision	Other considerations	[intervention]	[comparison]	Relative (95% CI)	Absolute (95% CI)		
1	randomised trials	serious[s]	not serious	not serious	serious[t]	none	40	40	-	SMD **1.69 SD higher** (1.18 higher to 2.2 higher)	⨁⨁◯◯ Low	IMPORTANT
**Health-related quality of life (physical score)**												
1	randomised trials	serious[s]	not serious	not serious	serious[t]	none	40	40	-	SMD **0.74 SD higher** (0.29 higher to 1.19 higher)	⨁⨁◯◯ Low	IMPORTANT

CI: confidence interval; **MD:** mean difference; **RR:** risk ratio; **SMD:** standardized mean difference

Explanations

a. Most studies were at risk of bias as a result of bias arising from randomization process and deviation from the intended intervention. Although studies with a low risk of bias showed similar result to the main analysis and we did not find a significant difference between studies that had high or low risk of bias, we conservatively chose to rate down the certainty of the evidence. Downgraded.

b. There was evidence of a substantial heterogeneity in the data (I^2=88%, Phet<0.001); however, out of 109 trials included in the analysis, 105 trials reported consistent decreasing effects. Indeed, there was high consistency in terms of the direction of the effects and, thus, the large heterogeneity in the data is due to difference in the magnitude of the effects. Not downgraded.

© 2024 Jayedi A et al. *JAMA Network Open*.

c. Serious imprecision since the point estimates did not surpass the threshold set as MID for weight loss (4.4 kg) either in linear or in the nonlinear dose-response analyses. Downgraded.

d. There was evidence of a substantial heterogeneity in the data (I^2=88%, Phet<0.001); however, out of 62 trials included in the analysis, 58 trials reported consistent decreasing effects. Indeed, there was high consistency in terms of the direction of the effects and, thus, the large heterogeneity in the data is due to difference in the magnitude of the effects. Not downgraded.

e. The point estimate in the nonlinear dose-response meta-analysis surpassed the threshold set as MID for waist circumference (2 cm). Not downgraded.

f. There was evidence of a substantial heterogeneity in the data (I^2=83%, Phet<0.001); however, out of 65 trials included in the analysis, 62 trials reported consistent decreasing effects. Indeed, there was high consistency in terms of the direction of the effects and, thus, the large heterogeneity in the data is due to difference in the magnitude of the effects. In addition, heterogeneity was partly explained by a priori subgroup analysis by intensity of aerobic exercise with pre-specified direction of the effects across subgroups. Not downgraded.

g. The point estimate in the nonlinear dose-response meta-analysis surpassed the threshold set as MID for body fat percentage (2%). Not downgraded.

h. Most studies were at risk of bias as a result of bias arising from randomization process and deviation from the intended intervention and we t found a significant difference between studies that had high or low risk of bias. Downgraded.

i. There was evidence of a substantial heterogeneity in the data (I^2=85%, Phet<0.001); however, out of 50 trials included in the analysis, 49 trials reported consistent decreasing effects. Indeed, there was high consistency in terms of the direction of the effects and, thus, the large heterogeneity in the data is due to difference in the magnitude of the effects. Not downgraded.

j. The point estimate in the nonlinear dose-response meta-analysis surpassed the threshold set as MID for fat mass (2 kg). Not downgraded.

k. There was evidence of a substantial heterogeneity in the data (I^2=66%, Phet<0.001); however, out of 26 trials included in the analysis, 25 trials reported consistent decreasing effects. Indeed, there was high consistency in terms of the direction of the effects and, thus, the large heterogeneity in the data is due to difference in the magnitude of the effects. Not downgraded.

l. The point estimate in the nonlinear dose-response meta-analysis surpassed the threshold set as MID for visceral adipose tissue (5 cm^2). Not downgraded.

m. There was evidence of a substantial heterogeneity in the data (I^2=74%, Phet<0.001); however, out of 27 trials included in the analysis, 26 trials reported consistent decreasing effects. Indeed, there was high consistency in terms of the direction of the effects and, thus, the large heterogeneity in the data is due to difference in the magnitude of the effects. Not downgraded.

n. The point estimate in the nonlinear dose-response meta-analysis surpassed the threshold set as MID for subcutaneous adipose tissue (5 cm^2). Not downgraded.

o. Most studies were at risk of bias as a result of bias arising from randomization process and selective outcome reporting. Downgraded.

p. Serious imprecision since the upper bound of the 95%CI in the absolute effects surpassed the threshold set as important harm (2%). Downgraded.

© 2024 Jayedi A et al. *JAMA Network Open*.

q. Serious imprecision since the upper bound of the 95%CI in the absolute effects surpassed the threshold set as important harm (2%), and the 95%CI was very wide. Downgraded by two levels.

r. Serious imprecision since the upper bound of the 95%CI in the absolute effects surpassed the threshold set as important harm (-2%). Downgraded.

s. Serious risk of bias as a result of bias arising from selective outcome reporting. Downgraded.

t. Serious imprecision since the number of participants in the trial was smaller than the optimal information size (n=400 in each study arm). Downgraded.

© 2024 Jayedi A et al. *JAMA Network Open*.

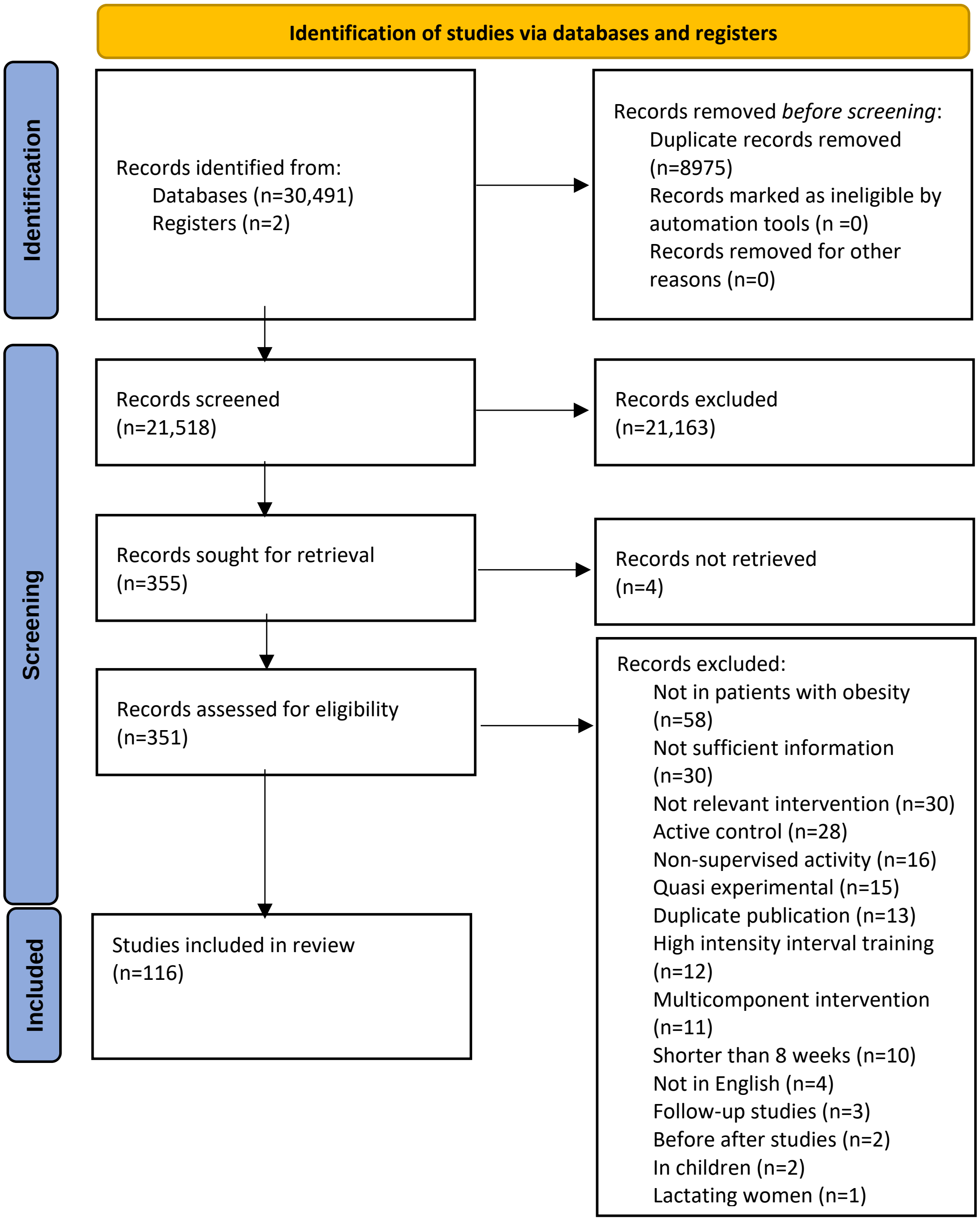

eFigure 1. Literature search and study selection process

© 2024 Jayedi A et al. *JAMA Network Open*.

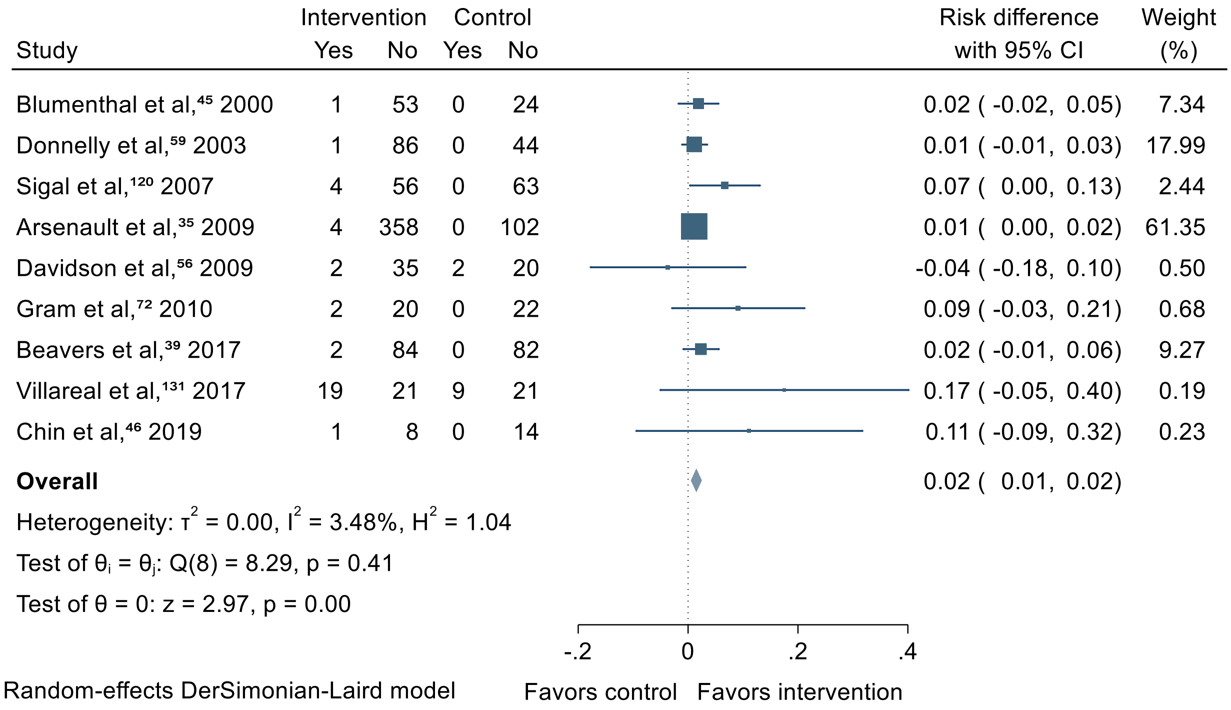

eFigure 2. Absolute effect of aerobic exercise on adverse events in the study participants

© 2024 Jayedi A et al. *JAMA Network Open*.

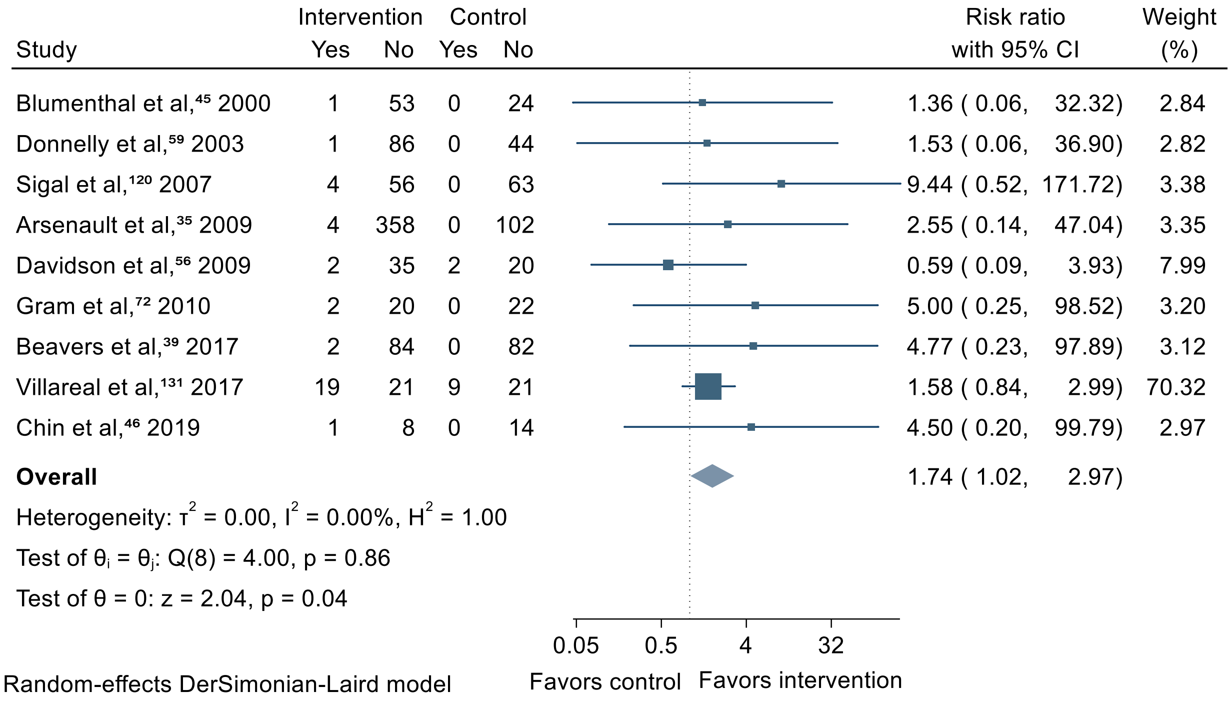

eFigure 3. Relative effect of aerobic exercise on adverse events in the study participants.

© 2024 Jayedi A et al. *JAMA Network Open*.

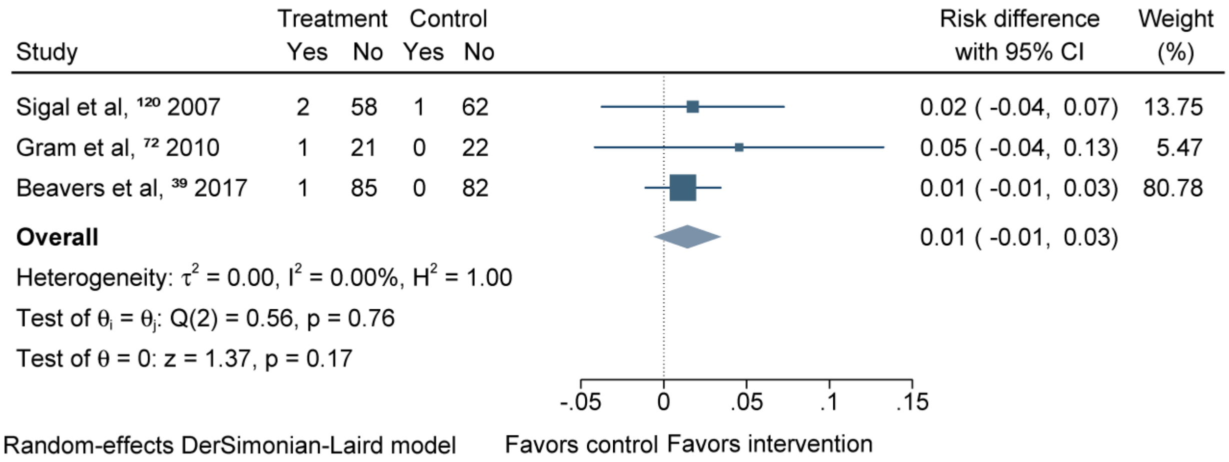

eFigure 4. Absolute effect of aerobic exercise on hypoglycemic reactions in the study participants.

© 2024 Jayedi A et al. *JAMA Network Open*.

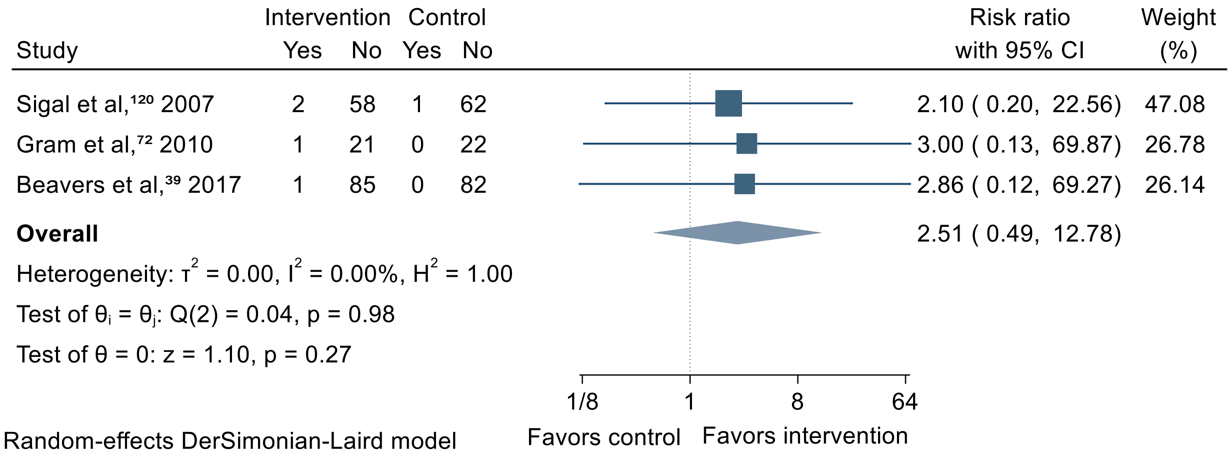

eFigure 5. Relative effect of aerobic exercise on hypoglycemic reactions in the study participants

© 2024 Jayedi A et al. *JAMA Network Open*.

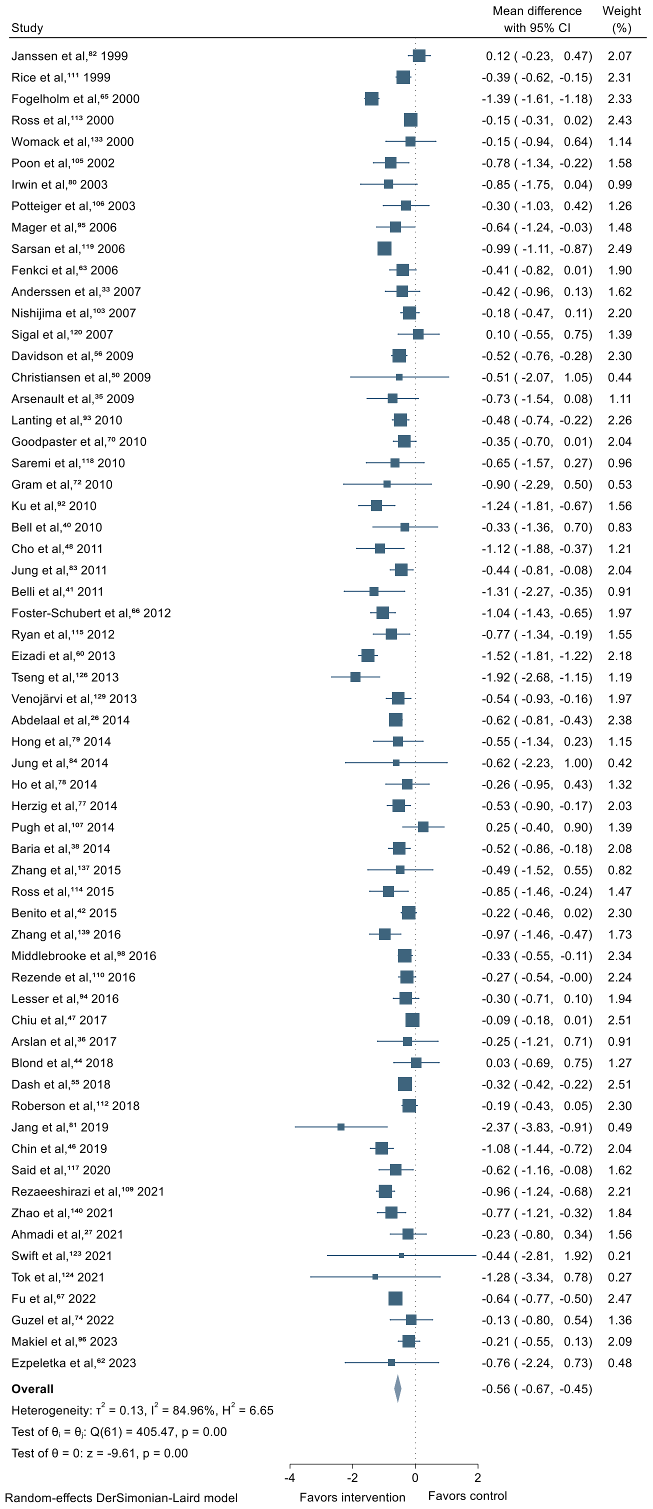

eFigure 6. Association of aerobic exercise (30 min/week) with waist circumference (cm)

© 2024 Jayedi A et al. *JAMA Network Open*.

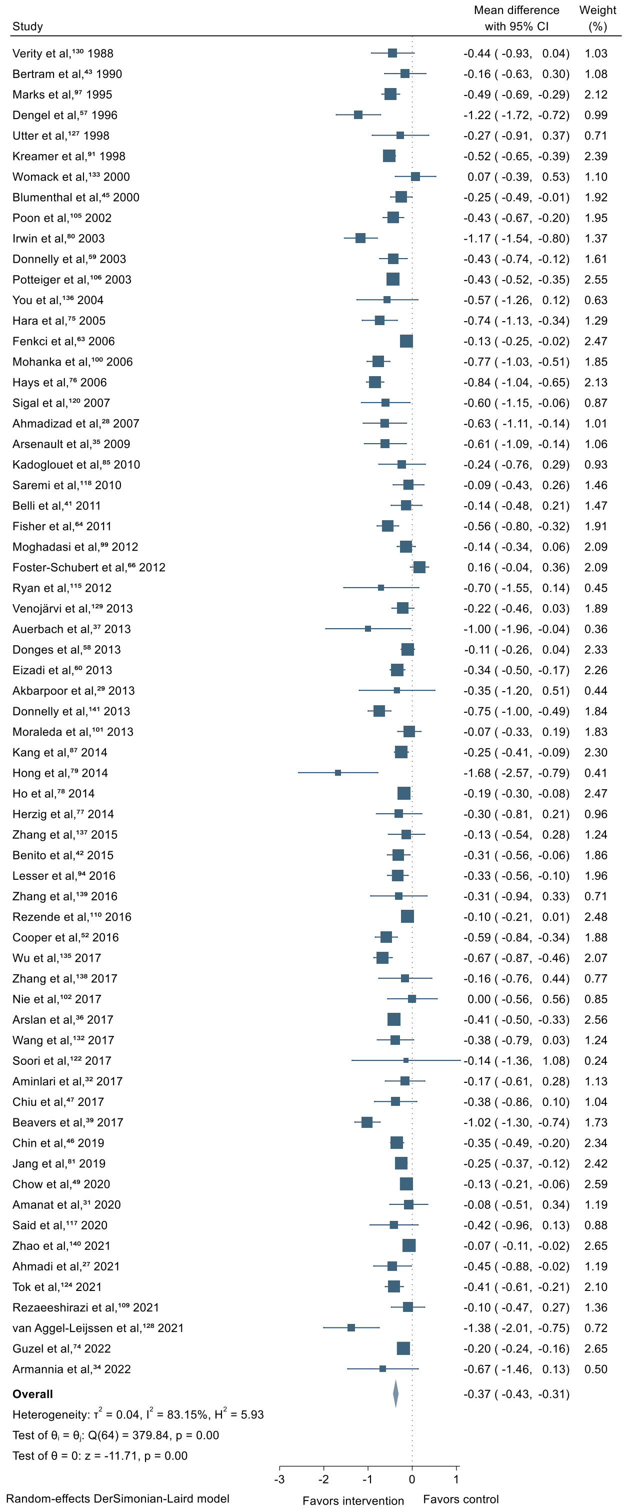

eFigure 7. Association of aerobic exercise (30 min/week) with body fat percentage (%)

© 2024 Jayedi A et al. *JAMA Network Open*.

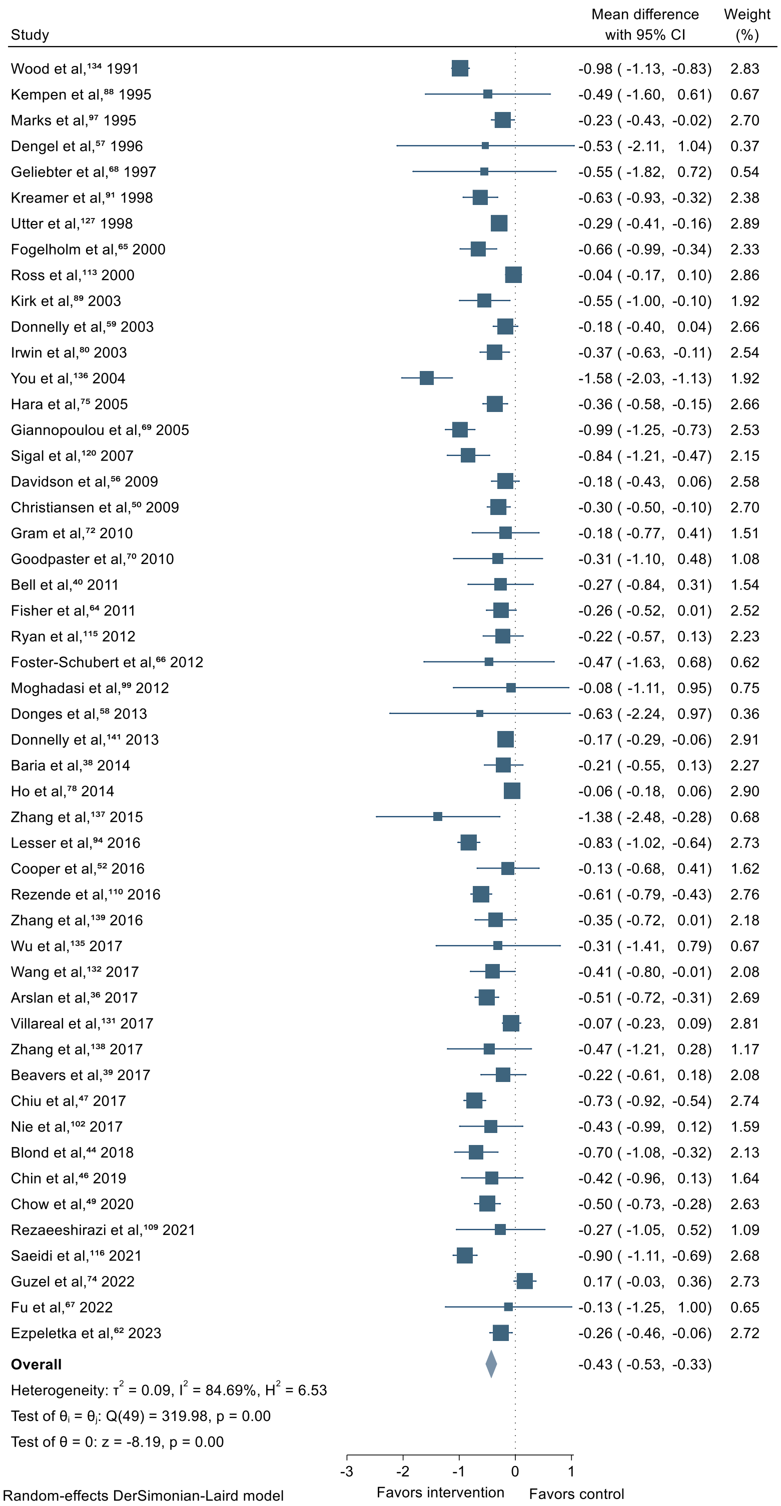

eFigure 8. Association of aerobic exercise (30 min/week) with body fat mass (kg)

© 2024 Jayedi A et al. *JAMA Network Open*.

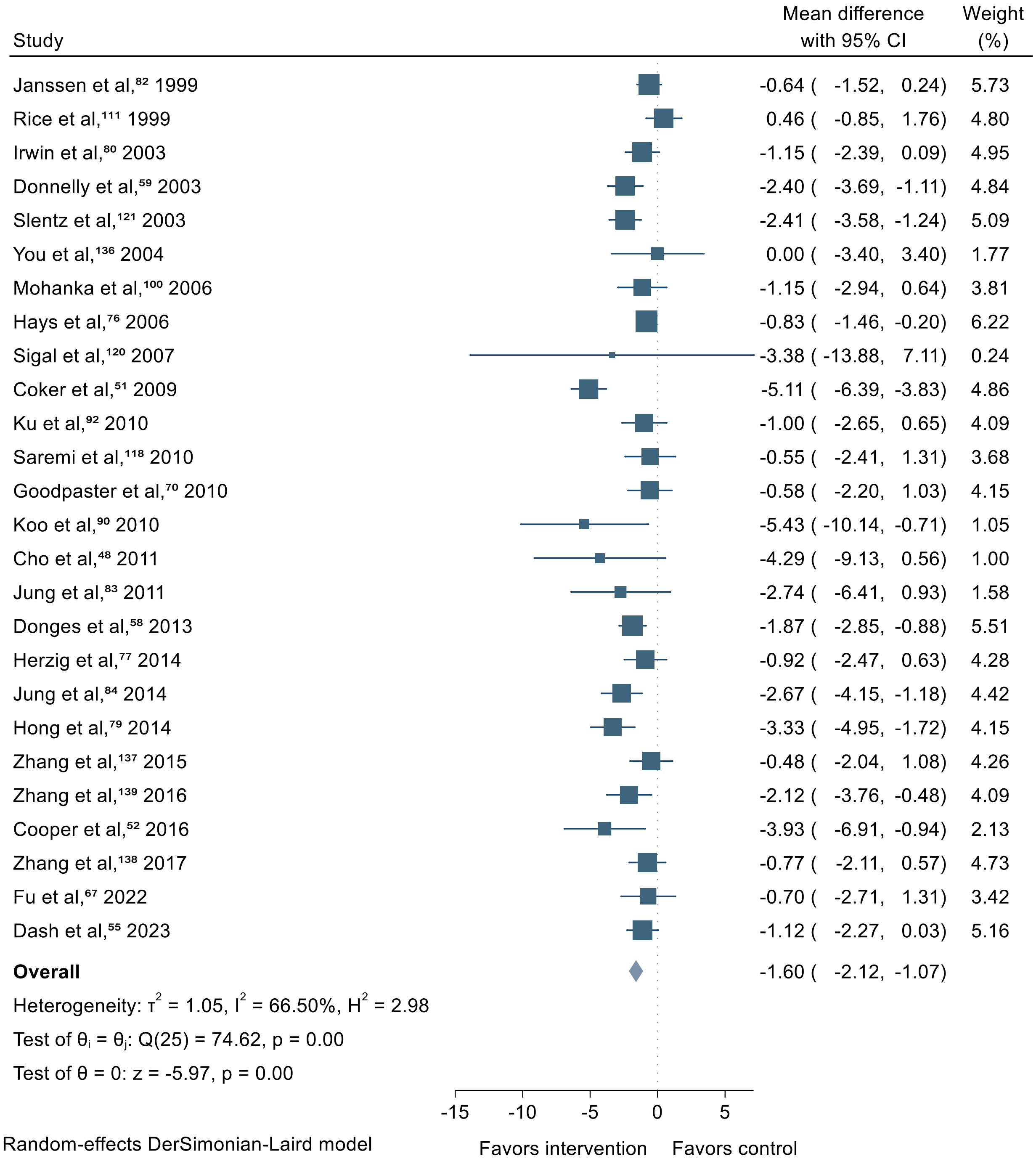

eFigure 9. Association of aerobic exercise (30 min/week) with visceral adipose tissue (cm^2)

© 2024 Jayedi A et al. *JAMA Network Open*.

Study	Mean difference with 95% CI	Weight (%)
Rice et al,[111] 1999	0.06 [-0.30, 0.42]	9.09
Janssen et al,[82] 1999	-1.06 [-1.78, -0.34]	7.74
Slentz et al,[121] 2003	-2.76 [-7.71, 2.19]	0.75
Irwin et al,[80] 2003	-4.00 [-19.07, 11.07]	0.09
Donnelly et al,[59] 2003	-2.95 [-8.03, 2.13]	0.72
You et al,[136] 2004	-1.24 [-2.16, -0.32]	6.87
Mohanka et al,[100] 2006	-0.67 [-1.98, 0.65]	5.27
Hays et al,[76] 2006	-3.84 [-7.33, -0.35]	1.41
Sigal et al,[120] 2007	-1.72 [-3.47, 0.03]	3.91
Ku et al,[92] 2010	-5.00 [-8.76, -1.24]	1.23
Saremi et al,[118] 2010	-1.20 [-3.86, 1.46]	2.20
Goodpaster et al,[70] 2010	-1.40 [-2.18, -0.62]	7.50
Koo et al,[90] 2010	-2.51 [-5.71, 0.69]	1.63
Cho et al,[48] 2011	-5.49 [-11.37, 0.39]	0.55
Fisher et al,[64] 2011	-1.50 [-5.59, 2.59]	1.06
Ryan et al,[115] 2012	-1.11 [-3.43, 1.21]	2.69
Moghadasi et al,[99] 2012	-0.16 [-0.47, 0.15]	9.24
Jung et al,[83] 2012	-4.22 [-14.45, 6.00]	0.19
Donges et al,[58] 2013	-3.84 [-6.32, -1.36]	2.44
Jung et al,[84] 2014	-0.52 [-2.25, 1.21]	3.95
Zhang et al,[137] 2015	-2.42 [-4.56, -0.28]	3.02
Cooper et al,[52] 2016	-2.40 [-5.96, 1.16]	1.36
Zhang et al,[139] 2016	-4.00 [-6.70, -1.30]	2.14
Lesser et al,[94] 2016	-2.33 [-3.20, -1.47]	7.12
Zhang et al,[138] 2017	-1.70 [-2.47, -0.93]	7.52
Fu et al,[67] 2022	-4.22 [-8.28, -0.17]	1.08
Cuff et al,[54] 2023	-0.30 [-0.61, 0.01]	9.24
**Overall**	-1.37 [-1.82, -0.92]	

Heterogeneity: $\tau^2 = 0.54$, $I^2 = 71.41\%$, $H^2 = 3.50$

Test of $\theta_i = \theta_j$: $Q(26) = 90.93$, $p = 0.00$

Test of $\theta = 0$: $z = -5.99$, $p = 0.00$

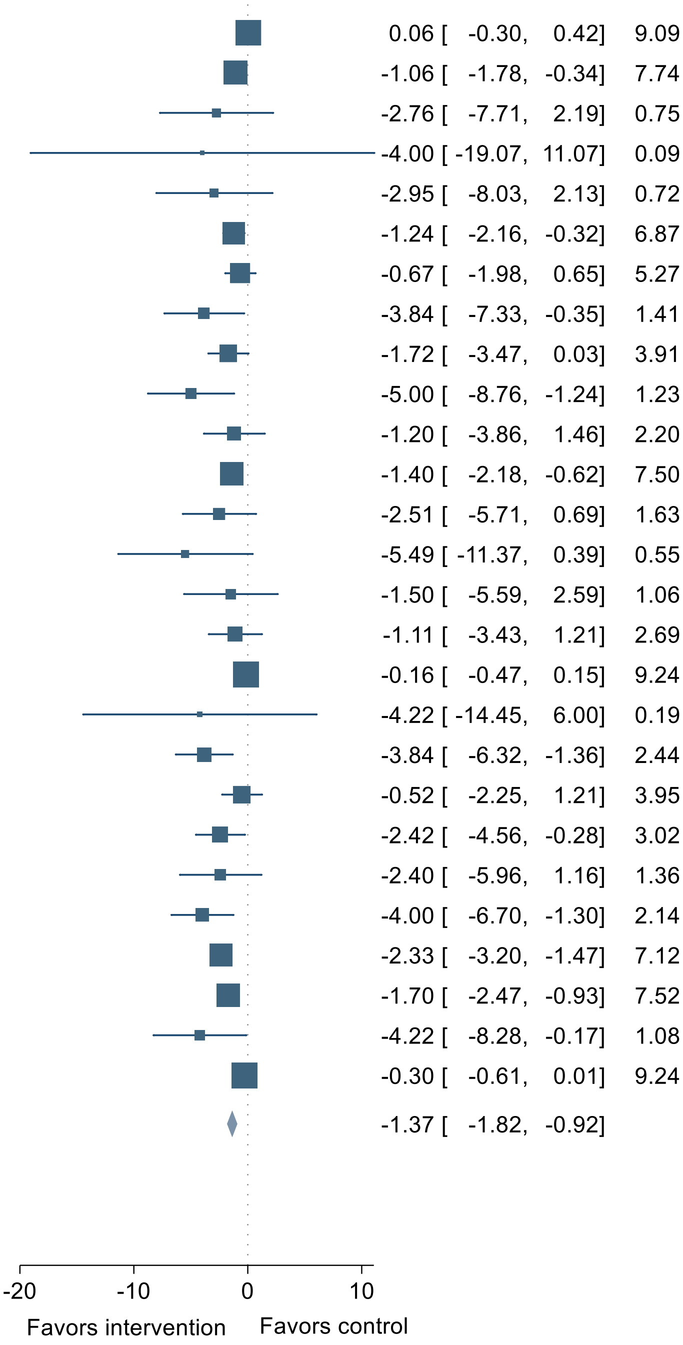

Random-effects DerSimonian-Laird model

eFigure 10. Association of aerobic exercise (30 min/week) with subcutaneous adipose tissue (cm^2)

© 2024 Jayedi A et al. *JAMA Network Open*.

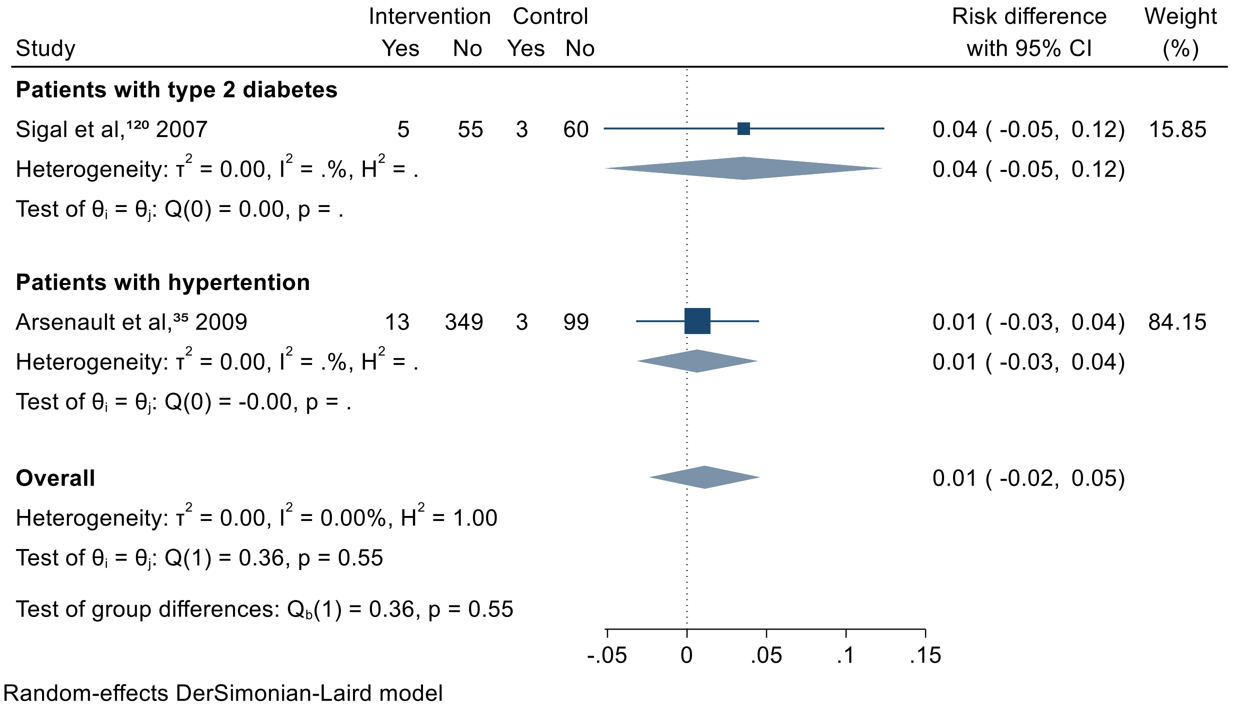

eFigure 11. Absolute effect of aerobic exercise on medication reduction

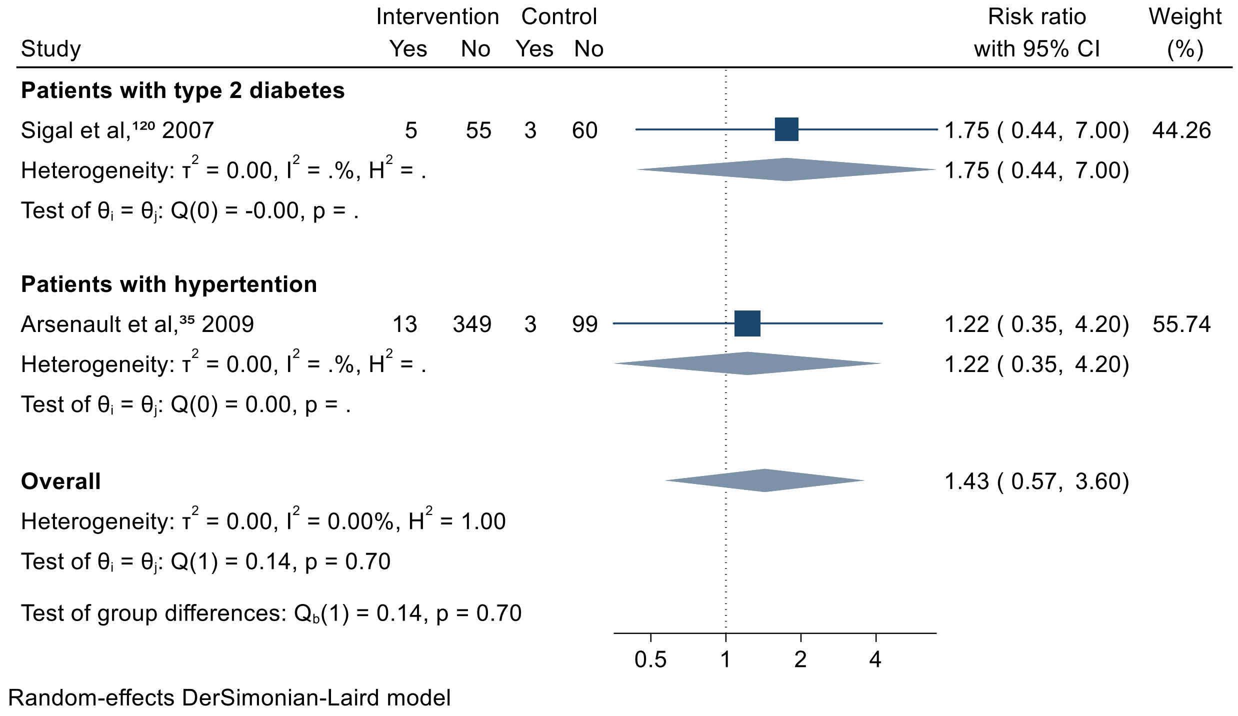

eFigure 12. Relative effect of aerobic exercise on medication reduction

© 2024 Jayedi A et al. *JAMA Network Open*.

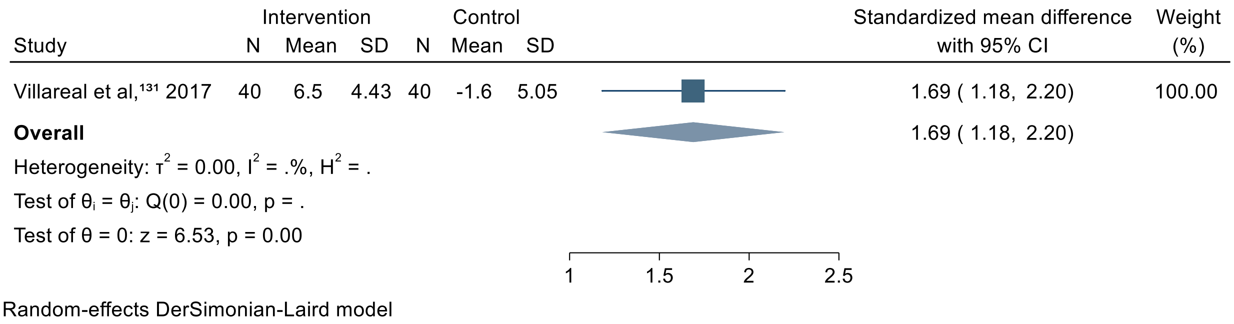

eFigure 13. Effect of aerobic exercise on health-related quality of life (mental score)

© 2024 Jayedi A et al. *JAMA Network Open*.

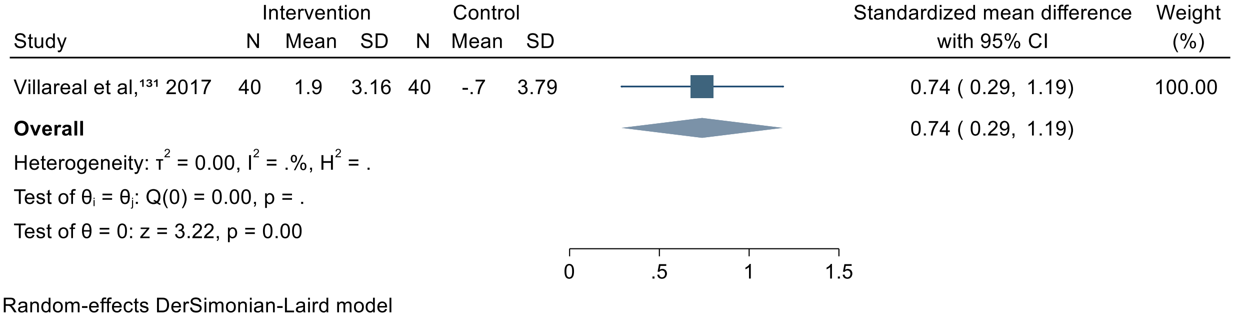

eFigure 14. Effect of aerobic exercise on health-related quality of life (physical score)

© 2024 Jayedi A et al. *JAMA Network Open*.

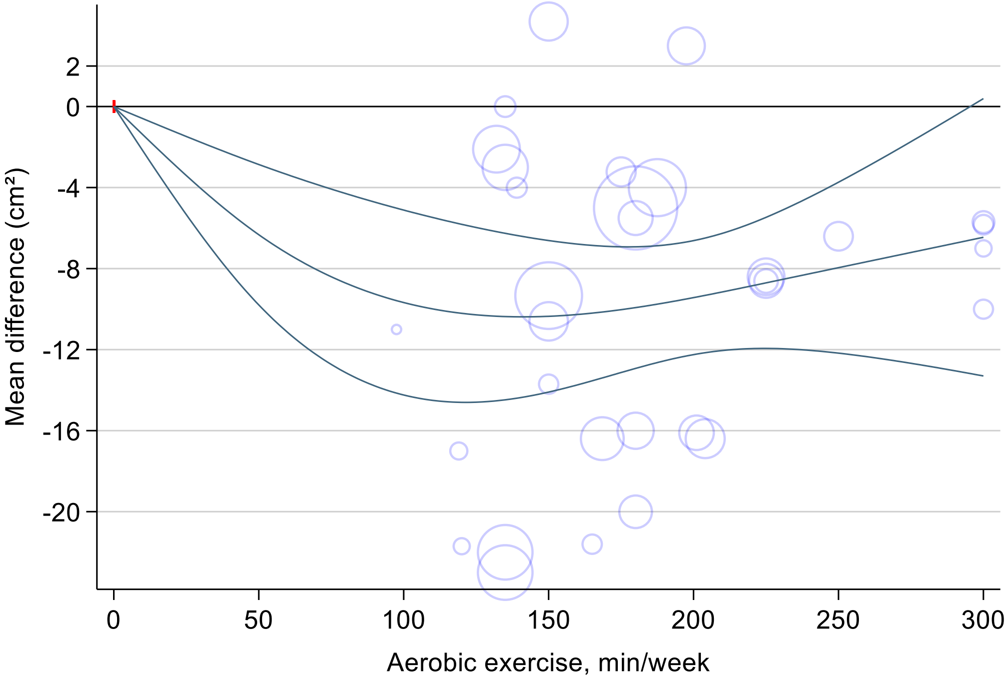

$P_{dose\text{-}response} < 0.001$; $P_{non\text{-}linearity} = 0.017$

eFigure 15. Dose-dependent association of aerobic exercise with visceral adipose tissue (cm^2). Solid lines represent nonlinear dose-response and its 95%CI. Circles represent relative risk point estimates for aerobic exercise from each study with circle size proportional to inverse of standard error. Small vertical red line is baseline dose across studies.

© 2024 Jayedi A et al. *JAMA Network Open*.

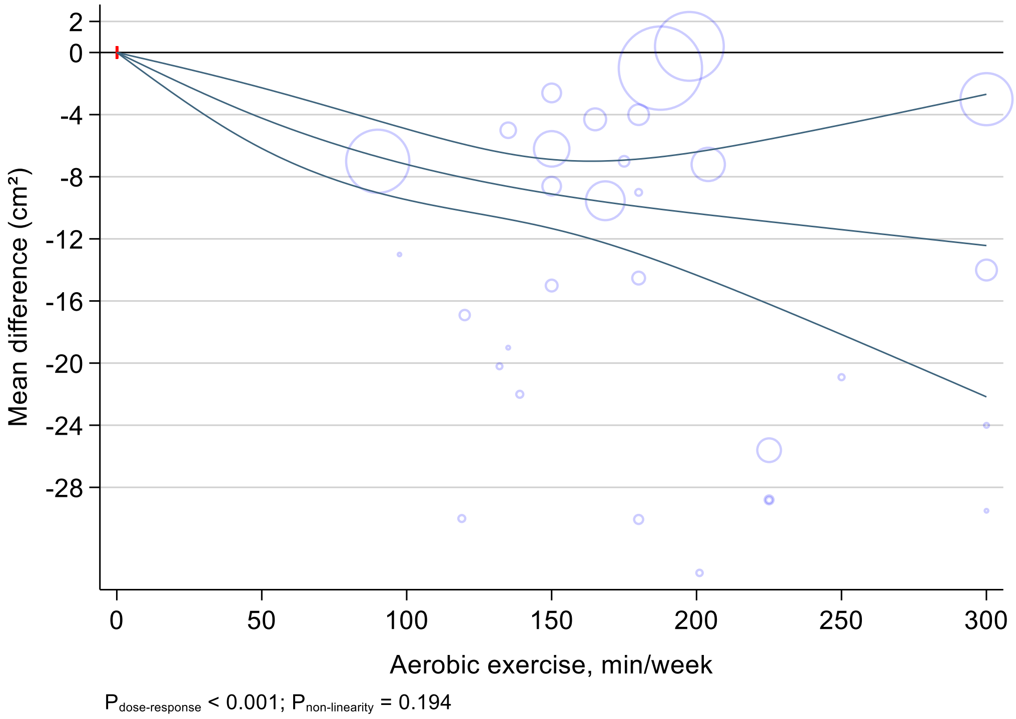

eFigure 16. Dose-dependent association of aerobic exercise with subcutaneous adipose tissue (cm^2). Solid lines represent nonlinear dose-response and its 95%CI. Circles represent relative risk point estimates for aerobic exercise from each study with circle size proportional to inverse of standard error. Small vertical red line is baseline dose across studies.

© 2024 Jayedi A et al. *JAMA Network Open*.

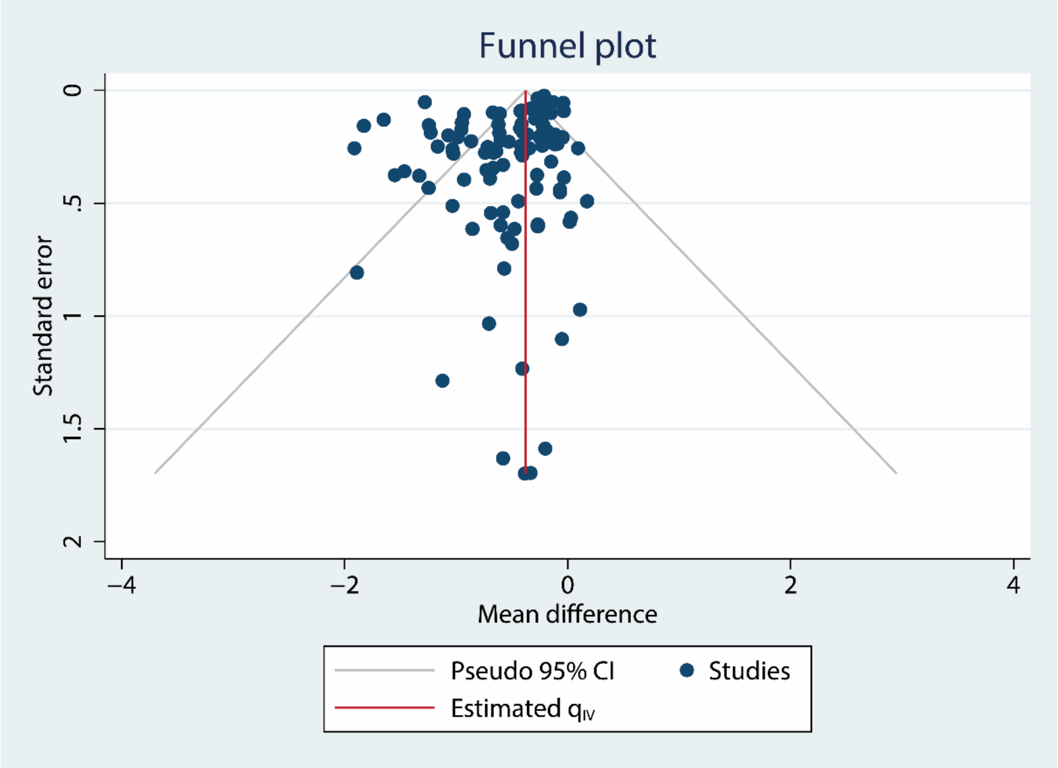

eFigure 17. Funnel plot of the association of aerobic exercise with body weight (Egger test = 0.42).

© 2024 Jayedi A et al. *JAMA Network Open*.

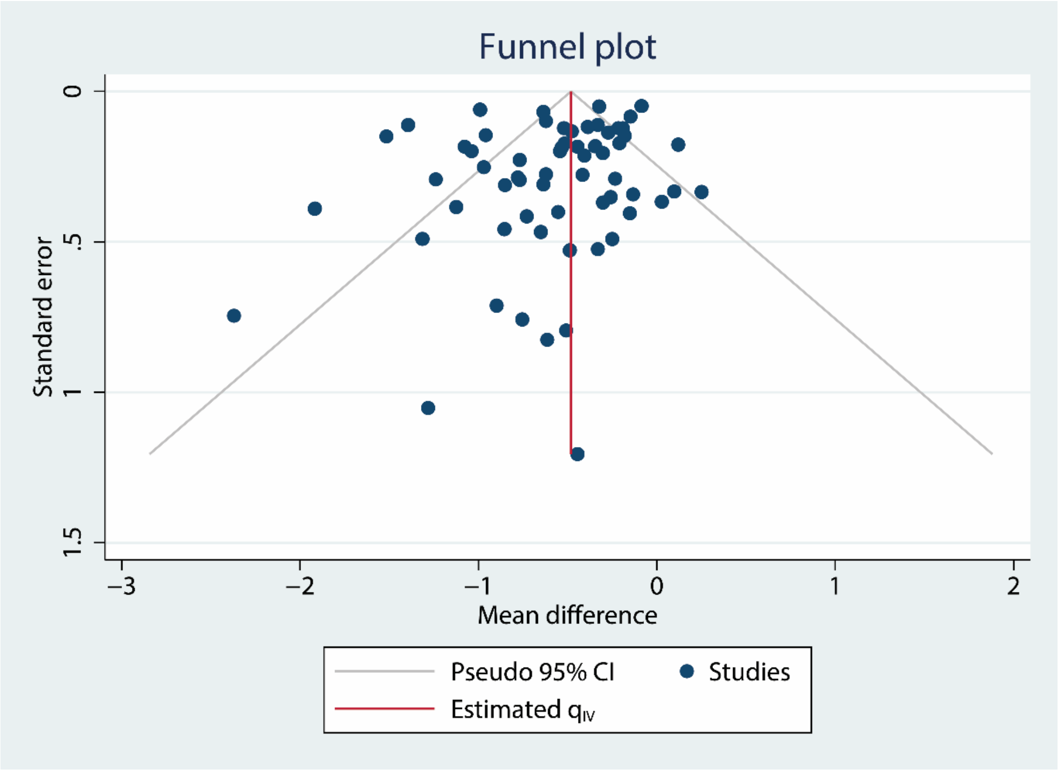

eFigure 18. Funnel plot of the association of aerobic exercise with waist circumference (Egger test = 0.21)

© 2024 Jayedi A et al. *JAMA Network Open*.

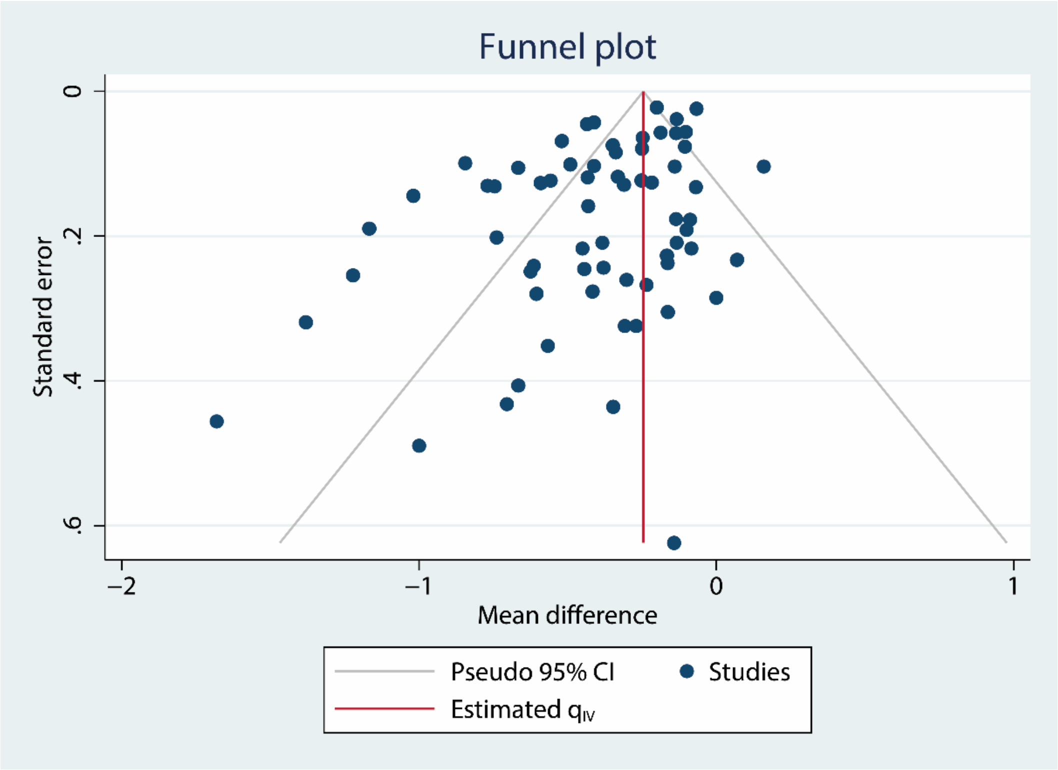

eFigure 19. Funnel plot of the association of aerobic exercise with body fat percentage (Egger test = 0.003). Trim-and-fill analysis found one potentially missing study, and the main effect size (mean difference = -0.37 %, 95%CI: -0.43, -0.31%; n = 65 trials) did not change after imputing this study (mean difference = -0.37 %, 95%CI: -0.43, -0.30%; n = 66 trials).

© 2024 Jayedi A et al. *JAMA Network Open*.

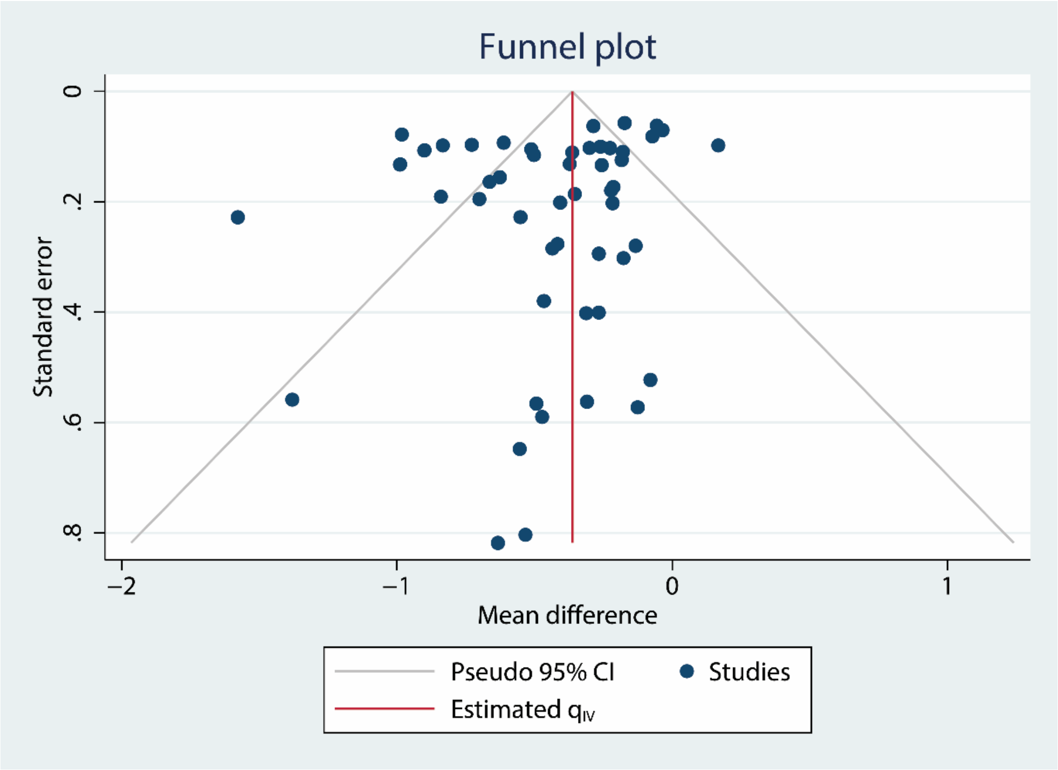

eFigure 20. Funnel plot of the association of aerobic exercise with body fat mass (Egger test = 0.55)

© 2024 Jayedi A et al. *JAMA Network Open*.

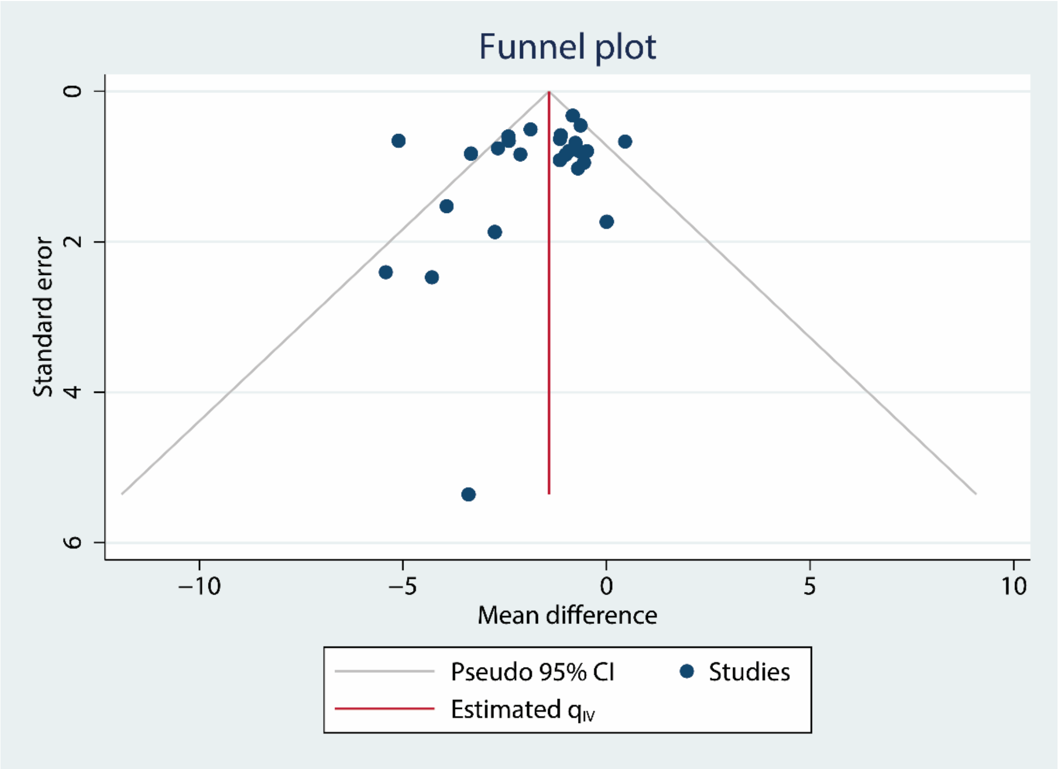

eFigure 21. Funnel plot of the association of aerobic exercise with visceral adipose tissue (Egger test = 0.15)

© 2024 Jayedi A et al. *JAMA Network Open*.

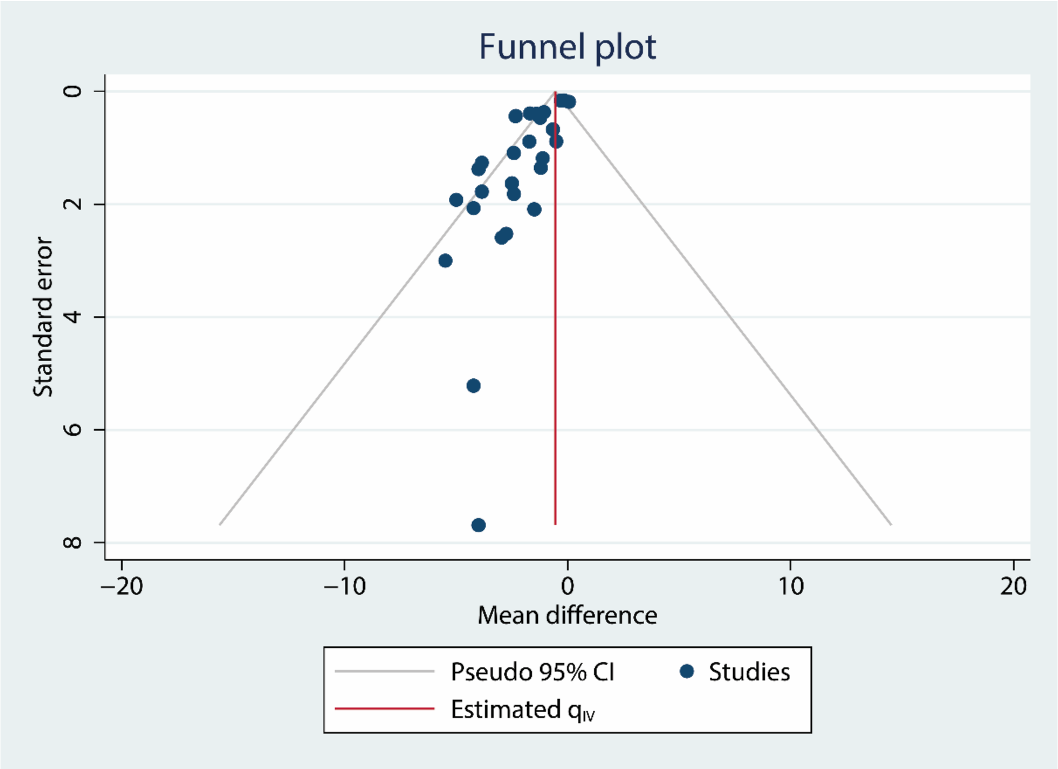

eFigure 22. Funnel plot of the association of aerobic exercise with subcutaneous adipose tissue (Egger's test <0.001). Trim-and-fill analysis found 11 potentially missing studies, and the main effect size (mean difference = -1.37 cm^2, 95%CI: -1.82, -0.92 cm^2; n = 27 trials) attenuated but remained statistically significant after imputing these studies (mean difference = -1.03 cm^2, 95%CI: -1.46, -0.60 cm^2; n = 38 trials).

© 2024 Jayedi A et al. *JAMA Network Open*.

eReferences.

1. Higgins JP, Thomas J, Chandler J, et al. *Cochrane handbook for systematic reviews of interventions*. John Wiley & Sons; 2019.
2. Hultcrantz M, Rind D, Akl EA, et al. The GRADE Working Group clarifies the construct of certainty of evidence. *Journal of clinical epidemiology*. 2017;87:4-13.
3. Zeng L, Brignardello-Petersen R, Hultcrantz M, et al. GRADE guidelines 32: GRADE offers guidance on choosing targets of GRADE certainty of evidence ratings. *Journal of Clinical Epidemiology*. 2021;137:163-175.
4. Ge L, Sadeghirad B, Ball GD, et al. Comparison of dietary macronutrient patterns of 14 popular named dietary programmes for weight and cardiovascular risk factor reduction in adults: systematic review and network meta-analysis of randomised trials. *bmj*. 2020;369
5. Jovanovski E, Mazhar N, Komishon A, et al. Effect of viscous fiber supplementation on obesity indicators in individuals consuming calorie-restricted diets: A systematic review and meta-analysis of randomized controlled trials. *European journal of nutrition*. 2021;60:101-112.
6. Johnston BC, Zeraatkar D, Steen J, et al. Saturated fat and human health: a protocol for a methodologically innovative systematic review and meta-analysis to inform public health nutrition guidelines. *Systematic Reviews*. 2023;12(1):1-8.
7. Iorio A, Spencer FA, Falavigna M, et al. Use of GRADE for assessment of evidence about prognosis: rating confidence in estimates of event rates in broad categories of patients. *BMJ : British Medical Journal*. 2015;350:h870. doi:10.1136/bmj.h870
8. Rücker G, Schwarzer G, Carpenter JR, Schumacher M. Undue reliance on I2 in assessing heterogeneity may mislead. *BMC Medical Research Methodology*. 2008/11/27 2008;8(1):79. doi:10.1186/1471-2288-8-79
9. Guyatt G, Zhao Y, Mayer M, et al. GRADE guidance 36: updates to GRADE's approach to addressing inconsistency. *Journal of Clinical Epidemiology*. 2023/06/01/ 2023;158:70-83. doi:https://doi.org/10.1016/j.jclinepi.2023.03.003
10. Guyatt GH, Oxman AD, Montori V, et al. GRADE guidelines: 5. Rating the quality of evidence—publication bias. *Journal of Clinical Epidemiology*. 2011/12/01/ 2011;64(12):1277-1282. doi:https://doi.org/10.1016/j.jclinepi.2011.01.011
11. Abe T, Kawakami Y, Sugita M, Fukunaga T. Relationship between training frequency and subcutaneous and visceral fat in women. *Medicine and science in sports and exercise*. 1997;29(12):1549-1553.
12. Aldred H, Hardman A, Taylor S. Influence of 12 weeks of training by brisk walking on postprandial lipemia and insulinemia in sedentary middle-aged women. *Metabolism*. 1995;44(3):390-397.
13. Anderson AG, Murphy MH, Murtagh E, Nevill A. An 8-week randomized controlled trial on the effects of brisk walking, and brisk walking with abdominal electrical muscle stimulation on anthropometric, body composition, and self-perception measures in sedentary adult women. *Psychology of Sport and Exercise*. 2006;7(5):437-451.
14. Asikainen TM, Miilunpalo S, Oja P, Rinne M, Pasanen M, Vuori I. Walking trials in postmenopausal women: effect of one vs two daily bouts on aerobic fitness. *Scandinavian journal of medicine & science in sports*. 2002;12(2):99-105.
15. Baker G, Gray SR, Wright A, et al. The effect of a pedometer-based community walking intervention" Walking for Wellbeing in the West" on physical activity levels and health outcomes: a 12-week randomized controlled trial. *International Journal of Behavioral Nutrition and Physical Activity*. 2008;5:1-15.
16. Bonanno JA, Lies JE. Effects of physical training on coronary risk factors. *The American Journal of Cardiology*. 1974;33(6):760-764.

© 2024 Jayedi A et al. *JAMA Network Open*.

17. Boudou P, Sobngwi E, Mauvais-Jarvis F, Vexiau P, Gautier J. Absence of exercise-induced variations in adiponectin levels despite decreased abdominal adiposity and improved insulin sensitivity in type 2 diabetic men. *European journal of endocrinology*. 2003;149(5):421-424.
18. Braith RW, Pollock ML, Lowenthal DT, Graves JE, Limacher MC. Moderate-and high-intensity exercise lowers blood pressure in normotensive subjects 60 to 79 years of age. *The American journal of cardiology*. 1994;73(15):1124-1128.
19. Brosseau L, Wells GA, Kenny GP, et al. The implementation of a community-based aerobic walking program for mild to moderate knee osteoarthritis (OA): a knowledge translation (KT) randomized controlled trial (RCT): Part I: The Uptake of the Ottawa Panel clinical practice guidelines (CPGs). *BMC public health*. 2012;12(1):1-14.
20. Brown JC, Kontos D, Schnall MD, Wu S, Schmitz KH. The dose–response effects of aerobic exercise on body composition and breast tissue among women at high risk for breast cancer: a randomized trial. *Cancer Prevention Research*. 2016;9(7):581-588.
21. Butcher LR, Thomas A, Backx K, Roberts A, Webb R, Morris K. Low-intensity exercise exerts beneficial effects on plasma lipids via PPARγ. *Medicine & Science in Sports & Exercise*. 2008;40(7):1263-1270.
22. Buyukyazi G. The effects of eight-week walking programs of two different intensities on serum lipids and circulating markers of collagen remodelling in humans. *Science & sports*. 2008;23(3-4):162-169.
23. Chang JS, Namkung J. Effects of exercise intervention on mitochondrial stress biomarkers in metabolic syndrome patients: a randomized controlled trial. *International journal of environmental research and public health*. 2021;18(5):2242.
24. Choi KM, Han KA, Ahn HJ, et al. Effects of exercise on sRAGE levels and cardiometabolic risk factors in patients with type 2 diabetes: a randomized controlled trial. *The Journal of Clinical Endocrinology & Metabolism*. 2012;97(10):3751-3758.
25. Devi LBS, Singh TI. Effect of aerobic training on body composition among sedentary women of Manipur. *Int J Hum Mov Sports Sci*. 2021;9(4):712-6.
26. DiPietro L, Seeman TE, Stachenfeld NS, Katz LD, Nadel ER. Moderate-intensity aerobic training improves glucose tolerance in aging independent of abdominal adiposity. *Journal of the American Geriatrics Society*. 1998;46(7):875-879.
27. Donges C, Duffield R, Drinkwater E. Effects of resistance or aerobic exercise training on interleukin-6, C-reactive protein, and body composition. *Medicine and science in sports and exercise*. 2010;42(2):304-313.
28. Du X, Zhang C, Zhang X, Qi Z, Cheng S, Le S. The Impact of Nordic Walking on Bone Properties in Postmenopausal Women with Pre-Diabetes and Non-Alcohol Fatty Liver Disease. *International Journal of Environmental Research and Public Health*. 2021;18(14):7570.
29. Duncan JJ, Gordon NF, Scott CB. Women walking for health and fitness: how much is enough? *Jama*. 1991;266(23):3295-3299.
30. Fantin F, Rossi A, Morgante S, et al. Supervised walking groups to increase physical activity in elderly women with and without hypertension: effect on pulse wave velocity. *Hypertension Research*. 2012;35(10):988-993.
31. Friedenreich CM, Woolcott C, McTiernan A, et al. Adiposity changes after a 1-year aerobic exercise intervention among postmenopausal women: a randomized controlled trial. *International journal of obesity*. 2011;35(3):427-435.
32. Gillen JB, Martin BJ, MacInnis MJ, Skelly LE, Tarnopolsky MA, Gibala MJ. Twelve weeks of sprint interval training improves indices of cardiometabolic health similar to traditional endurance training despite a five-fold lower exercise volume and time commitment. *PloS one*. 2016;11(4):e0154075.

© 2024 Jayedi A et al. *JAMA Network Open*.

33. Gonçalves R, Motta-Santos D, Szmuchrowski L, et al. Combined training is not superior to strength and aerobic training to mitigate cardiovascular risk in adult healthy men. *Biology of Sport*. 2022;39(3):727-734.
34. Gonzalo-Encabo P, McNeil J, Pérez-López A, Valadés D, Courneya KS, Friedenreich CM. Dose-response effects of aerobic exercise on adiposity markers in postmenopausal women: pooled analyses from two randomized controlled trials. *International Journal of Obesity*. 2021;45(6):1298-1309.
35. Gradinariu V, Ard J, van Dam RM. Effects of dietary quality, physical activity and weight loss on glucose homeostasis in persons with and without prediabetes in the PREMIER trial. *Diabetes, Obesity and Metabolism*. 2023;25(9):2714-2722.
36. Helle M-L, de Faire U, Berglund B, Hamsten A, Krakau I. Diet and exercise are equally effective in reducing risk for cardiovascular disease. Results of a randomized controlled study in men with slightly to moderately raised cardiovascular risk factors. *Atherosclerosis*. 1993;103(1):81-91.
37. Hooshmand Moghadam B, Golestani F, Bagheri R, et al. The effects of high-intensity interval training vs. moderate-intensity continuous training on inflammatory markers, body composition, and physical fitness in overweight/obese survivors of breast cancer: A randomized controlled clinical trial. *Cancers*. 2021;13(17):4386.
38. Isaacs A, Critchley J, Tai SS, et al. Exercise Evaluation Randomised Trial (EXERT): a randomised trial comparing GP referral for leisure centre-based exercise, community-based walking and advice only. *HEALTH TECHNOLOGY ASSESSMENT-SOUTHAMPTON-*. 2007;11(10)
39. Karstoft K, Winding K, Knudsen SH, et al. The effects of free-living interval-walking training on glycemic control, body composition, and physical fitness in type 2 diabetic patients: a randomized, controlled trial. *Diabetes care*. 2013;36(2):228-236.
40. Kong J, Chen Y, Zheng Y, et al. Effectiveness of a Worksite-Based Lifestyle Intervention on Employees' Obesity Control and Prevention in China: A Group Randomized Experimental Study. *International Journal of Environmental Research and Public Health*. 2022;19(11):6738.
41. Krustrup P, Nielsen JJ, Krustrup BR, et al. Recreational soccer is an effective health-promoting activity for untrained men. *British journal of sports medicine*. 2009;43(11):825-831.
42. Lambers S, Van Laethem C, Van Acker K, Calders P. Influence of combined exercise training on indices of obesity, diabetes and cardiovascular risk in type 2 diabetes patients. *Clinical Rehabilitation*. 2008;22(6):483-492.
43. Letnes JM, Berglund I, Johnson KE, et al. Effect of 5 years of exercise training on the cardiovascular risk profile of older adults: the Generation 100 randomized trial. *European heart journal*. 2022;43(21):2065-2075.
44. Li M, Zheng Q, Miller JD, et al. Aerobic training reduces pancreatic fat content and improves β-cell function: A randomized controlled trial using IDEAL-IQ magnetic resonance imaging. *Diabetes/Metabolism Research and Reviews*. 2022;38(4):e3516.
45. Libardi CA, De Souza GV, Cavaglieri CR, Madruga VA, Chacon-Mikahil M. Effect of resistance, endurance, and concurrent training on TNF-a, IL-6, and CRP. *Med Sci Sports Exerc*. 2012;44(5):50-6.
46. Liu L, Ma X, Huiwen X, Ruan S, Yuan X. Comparing the effects of 12 months aerobic exercise and resistance training on glucose metabolism among prediabetes phenotype: A explorative randomized controlled trial. *Primary Care Diabetes*. 2021;15(2):340-346.
47. Luo X, Wang Z, Li B, Zhang X, Li X. Effect of resistance vs. aerobic exercise in pre-diabetes: an RCT. *Trials*. 2023;24(1):1-10.
48. Martins RA, Neves AP, Coelho-Silva MJ, Veríssimo MT, Teixeira AM. The effect of aerobic versus strength-based training on high-sensitivity C-reactive protein in older adults. *European journal of applied physiology*. 2010;110:161-169.
49. McTiernan A, Sorensen B, Irwin ML, et al. Exercise effect on weight and body fat in men and women. *Obesity*. 2007;15(6):1496-1512.

50. Mendham AE, Duffield R, Marino F, Coutts AJ. Small-sided games training reduces CRP, IL-6 and leptin in sedentary, middle-aged men. *European journal of applied physiology*. 2014;114:2289-2297.
51. Mohammadi A, Bijeh N, Moazzami M, khodaei K, Rahimi N. Effect of exercise training on spexin level, appetite, lipid accumulation product, visceral adiposity index, and body composition in adults with type 2 diabetes. *Biological Research For Nursing*. 2022;24(2):152-162.
52. Negri C, Bacchi E, Morgante S, et al. Supervised walking groups to increase physical activity in type 2 diabetic patients. *Diabetes Care*. 2010;33(11):2333-2335.
53. Nybo L, Sundstrup E, Jakobsen MD, et al. High-intensity training versus traditional exercise interventions for promoting health. *Medicine & Science in Sports & Exercise*. 2010;42(10):1951-1958.
54. Omar N, Ahmad R, Mohd Shah M, Aminuddin A, Chellappan K. Amelioration of inflammation in young men with cardiovascular risks participating pedometer-based walking programme. *Med J Malaysia*. 2021;76(3):375.
55. Osei-Tutu KB, Campagna PD. The effects of short-vs. long-bout exercise on mood, VO2max., and percent body fat. *Preventive medicine*. 2005;40(1):92-98.
56. Posner JD, Gorman KM, Windsor-Landsberg L, et al. Low to moderate intensity endurance training in healthy older adults: physiological responses after four months. *Journal of the American Geriatrics Society*. 1992;40(1):1-7.
57. Ribeiro VB, Lopes IP, Dos Reis RM, et al. Continuous versus intermittent aerobic exercise in the improvement of quality of life for women with polycystic ovary syndrome: A randomized controlled trial. *Journal of health psychology*. 2021;26(9):1307-1317.
58. Rokling-Andersen MH, Reseland JE, Veierød MB, et al. Effects of long-term exercise and diet intervention on plasma adipokine concentrations. *The American journal of clinical nutrition*. 2007;86(5):1293-1301.
59. Seals DR, Tanaka H, Clevenger CM, et al. Blood pressure reductions with exercise and sodium restriction in postmenopausal women with elevated systolic pressure: role of arterial stiffness. *Journal of the American College of Cardiology*. 2001;38(2):506-513.
60. Short KR, Vittone JL, Bigelow ML, et al. Impact of aerobic exercise training on age-related changes in insulin sensitivity and muscle oxidative capacity. *Diabetes*. 2003;52(8):1888-1896.
61. Sirisunhirun P, Bandidniyamanon W, Jrerattakon Y, et al. Effect of a 12-week home-based exercise training program on aerobic capacity, muscle mass, liver and spleen stiffness, and quality of life in cirrhotic patients: a randomized controlled clinical trial. *BMC gastroenterology*. 2022;22(1):66.
62. Strasser B, Spreitzer A, Haber P. Fat loss depends on energy deficit only, independently of the method for weight loss. *Annals of Nutrition and Metabolism*. 2007;51(5):428-432.
63. Tofas T, Fatouros IG, Draganidis D, et al. Effects of cardiovascular, resistance and combined exercise training on cardiovascular, performance and blood redox parameters in coronary artery disease patients: An 8-month training-detraining randomized intervention. *Antioxidants*. 2021;10(3):409.
64. Tripette J, Gando Y, Murakami H, et al. Effect of a 1-year intervention comprising brief counselling sessions and low-dose physical activity recommendations in Japanese adults, and retention of the effect at 2 years: a randomized trial. *BMC Sports Science, Medicine and Rehabilitation*. 2021;13(1):1-19.
65. Vidanage D, Wasalathanthri S, Hettiarachchi P. Long-term aerobic and combined exercises enhance the satiety response and modulate the energy intake in patients with type 2 diabetes mellitus (T2DM): A randomized controlled trial. *BMC Sports Science, Medicine and Rehabilitation*. 2023;15(1):1-16.
66. Vieira ER, Cavalcanti FAdC, Civitella F, et al. Effects of exercise and diet on body composition and physical function in older Hispanics with type 2 diabetes. *International Journal of Environmental Research and Public Health*. 2021;18(15):8019.

© 2024 Jayedi A et al. *JAMA Network Open*.

67. Way KL, Sabag A, Sultana RN, et al. The effect of low-volume high-intensity interval training on cardiovascular health outcomes in type 2 diabetes: A randomised controlled trial. *International journal of cardiology*. 2020;320:148-154.
68. Zhang J, Chen G, Lu W, et al. Effects of physical exercise on health-related quality of life and blood lipids in perimenopausal women: a randomized placebo-controlled trial. *Menopause*. 2014;21(12):1269-1276.
69. Alves RC, Enes A, Follador L, Prestes J, da Silva SG. Effect of Different Training Programs at Self-Selected Intensity on Body Composition, Perceptual Responses and Fitness Outcomes in Obese Women. *International Journal of Exercise Science*. 2022;15(4):373.
70. Brandon LJ, Elliott-Lloyd MB. Walking, body composition, and blood pressure dose-response in African American and white women. *Ethnicity & disease*. 2006;16(3):675-681.
71. Brixius K, Schoenberger S, Ladage D, et al. Long-term endurance exercise decreases antiangiogenic endostatin signalling in overweight men aged 50–60 years. *British journal of sports medicine*. 2008;42(2):126-129.
72. Broskey NT, Martin CK, Burton JH, Church TS, Ravussin E, Redman LM. Effect of aerobic exercise-induced weight loss on the components of daily energy expenditure. *Medicine and science in sports and exercise*. 2021;53(10):2164.
73. Church TS, Earnest CP, Skinner JS, Blair SN. Effects of different doses of physical activity on cardiorespiratory fitness among sedentary, overweight or obese postmenopausal women with elevated blood pressure: a randomized controlled trial. *Jama*. 2007;297(19):2081-2091.
74. Cowan TE, Brennan AM, Stotz PJ, Clarke J, Lamarche B, Ross R. Separate effects of exercise amount and intensity on adipose tissue and skeletal muscle mass in adults with abdominal obesity. *Obesity*. 2018;26(11):1696-1703.
75. Gusi N, Reyes MC, Gonzalez-Guerrero JL, Herrera E, Garcia JM. Cost-utility of a walking programme for moderately depressed, obese, or overweight elderly women in primary care: a randomised controlled trial. *BMC public health*. 2008;8:1-10.
76. Hagan RD, Upton SJ, Wong L, Whittam J. The effects of aerobic conditioning and/or caloric restriction in overweight men and women. *Med Sci Sports Exerc*. 1986;18(1):87-94.
77. Houghton D, Thoma C, Hallsworth K, et al. Exercise reduces liver lipids and visceral adiposity in patients with nonalcoholic steatohepatitis in a randomized controlled trial. *Clinical Gastroenterology and Hepatology*. 2017;15(1):96-102. e3.
78. Irving BA, Davis CK, Brock DW, et al. Effect of exercise training intensity on abdominal visceral fat and body composition. *Medicine and science in sports and exercise*. 2008;40(11):1863.
79. Irving BA, Weltman J, Patrie JT, et al. Effects of exercise training intensity on nocturnal growth hormone secretion in obese adults with the metabolic syndrome. *The Journal of Clinical Endocrinology & Metabolism*. 2009;94(6):1979-1986.
80. Jeffery RW, Wing RR, Thorson C, Burton LR. Use of personal trainers and financial incentives to increase exercise in a behavioral weight-loss program. *Journal of consulting and clinical psychology*. 1998;66(5):777.
81. Jorge MLMP, de Oliveira VN, Resende NM, et al. The effects of aerobic, resistance, and combined exercise on metabolic control, inflammatory markers, adipocytokines, and muscle insulin signaling in patients with type 2 diabetes mellitus. *Metabolism*. 2011;60(9):1244-1252.
82. Kearney TM, Murphy MH, Davison GW, O'Kane MJ, Gallagher AM. Accumulated brisk walking reduces arterial stiffness in overweight adults: evidence from a randomized control trial. *Journal of the American Society of Hypertension*. 2014;8(2):117-126.
83. Lee M-G, Park K-S, Kim D-U, Choi S-M, Kim H-J. Effects of high-intensity exercise training on body composition, abdominal fat loss, and cardiorespiratory fitness in middle-aged Korean females. *Applied Physiology, Nutrition, and Metabolism*. 2012;37(6):1019-1027.

© 2024 Jayedi A et al. *JAMA Network Open*.

84. Ligtenberg P, Hoekstra J, Bol E, Zonderland M, Erkelens D. Effects of physical training on metabolic control in elderly type 2 diabetes mellitus patients. *Clinical Science*. 1997;93(2):127-135.
85. LP de Oliveira V, de Freitas MM, P. de Paula T, et al. DASH diet vs. DASH diet plus physical activity in older patients with type 2 diabetes and high blood pressure: A randomized clinical trial. *Nutrition and Health*. 2022:02601060221124201.
86. Martin CK, Johnson WD, Myers CA, et al. Effect of different doses of supervised exercise on food intake, metabolism, and non-exercise physical activity: The E-MECHANIC randomized controlled trial. *The American journal of clinical nutrition*. 2019;110(3):583-592.
87. Moreira MM, Souza HPCd, Schwingel PA, Sá CKCd, Zoppi CC. Effects of aerobic and anaerobic exercise on cardiac risk variables in overweight adults. *Arquivos brasileiros de cardiologia*. 2008;91:219-226.
88. O'Hara RB, Baer JT, Pohlman RL, Laubach LL. The effect of a walking program on blood pressure response in African-American women. *ACSM's Health & Fitness Journal*. 2000;4(5):20-24.
89. Özbey-Yücel Ü, Aydoğan Z, Tokgoz-Yilmaz S, Uçar A, Ocak E, Beton S. The effects of diet and physical activity induced weight loss on the severity of tinnitus and quality of life: A randomized controlled trial. *Clinical Nutrition ESPEN*. 2021;44:159-165.
90. Reichkendler MH, Auerbach P, Rosenkilde M, et al. Exercise training favors increased insulin-stimulated glucose uptake in skeletal muscle in contrast to adipose tissue: a randomized study using FDG PET imaging. *American Journal of Physiology-Endocrinology and Metabolism*. 2013;305(4):E496-E506.
91. Rojo Tirado MA, Benito Peinado PJ, Peinado Lozano AB, Zapico García A, Calderon Montero FJ. Discriminant models to estimate the body weight loss after a six-month long diet and exercise-based intervention. *Journal of Sports Medicine And Physical Fitness*. 2015;56(1-2):79-84.
92. Rosenkilde M, Reichkendler M, Auerbach P, et al. Changes in peak fat oxidation in response to different doses of endurance training. *Scandinavian Journal of Medicine & Science in Sports*. 2015;25(1):41-52.
93. Ross R, Janssen I, Dawson J, et al. Exercise-induced reduction in obesity and insulin resistance in women: a randomized controlled trial. *Obesity research*. 2004;12(5):789-798.
94. Sentinelli F, La Cava V, Serpe R, et al. Positive effects of Nordic Walking on anthropometric and metabolic variables in women with type 2 diabetes mellitus. *Science & Sports*. 2015;30(1):25-32.
95. Sweeney M, Hill J, Heller P, Baney R, DiGirolamo M. Severe vs moderate energy restriction with and without exercise in the treatment of obesity: efficiency of weight loss. *The American journal of clinical nutrition*. 1993;57(2):127-134.
96. Volpe SL, Kobusingye H, Bailur S, Stanek E. Effect of diet and exercise on body composition, energy intake and leptin levels in overweight women and men. *Journal of the American College of Nutrition*. 2008;27(2):195-208.
97. Woodward A, Broom D, Dalton C, Metwally M, Klonizakis M. Supervised aerobic exercise training and increased lifestyle physical activity to reduce cardiovascular disease risk for women with polycystic ovary syndrome: a randomized controlled feasibility trial. *Journal of Physical Activity and Health*. 2022;19(6):436-445.
98. Yang R, Wan L, Zhu H, Peng Y. The effect of 12 week-maximum fat oxidation (FATmax) intensity exercise on microvascular function in obese patients with nonalcoholic fatty liver disease and its mechanism. *General Physiology & Biophysics*. 2023;42(3)
99. Alizadeh M, Dehghanizade J. The effect of functional training on level of brain-derived neurotrophic factor and functional performance in women with obesity. *Physiology & Behavior*. 2022;251:113798.
100. Amaro-Gahete FJ, Ponce-González JG, Corral-Pérez J, Velázquez-Díaz D, Lavie CJ, Jiménez-Pavón D. Effect of a 12-week concurrent training intervention on cardiometabolic health in obese men: a pilot study. *Frontiers in Physiology*. 2021;12:630831.

© 2024 Jayedi A et al. *JAMA Network Open*.

101. Bouamra M, Zouhal H, Ratel S, et al. Concurrent training promotes greater gains on body composition and components of physical fitness than single-mode training (endurance or resistance) in youth with obesity. *Frontiers in physiology*. 2022;13:895.
102. Boudou P, De Kerviler E, Erlich D, Vexiau P, Gautier J. Exercise training-induced triglyceride lowering negatively correlates with DHEA levels in men with type 2 diabetes. *International journal of obesity*. 2001;25(8):1108-1112.
103. Chagas EFB, Bonfim MR, Turi BC, Brondino NCM, Monteiro HL. Effect of moderate-intensity exercise on inflammatory markers among postmenopausal women. *Journal of physical activity and health*. 2017;14(6):479-485.
104. Chang K-V, Wu W-T, Huang K-C, Han D-S. Effectiveness of early versus delayed exercise and nutritional intervention on segmental body composition of sarcopenic elders-A randomized controlled trial. *Clinical Nutrition*. 2021;40(3):1052-1059.
105. De Jonge L, Moreira EA, Martin CK, Ravussin E, Team PC. Impact of 6-month caloric restriction on autonomic nervous system activity in healthy, overweight, individuals. *Obesity*. 2010;18(2):414-416.
106. Dieli-Conwright CM, Courneya KS, Demark-Wahnefried W, et al. Aerobic and resistance exercise improve patient-reported sleep quality and is associated with cardiometabolic biomarkers in Hispanic and non-Hispanic breast cancer survivors who are overweight or obese: results from a secondary analysis. *Sleep*. 2021;44(10):zsab111.
107. Dorling JL, Höchsmann C, Tudor-Locke C, Beyl R, Martin CK. Effect of an office-based intervention on visceral adipose tissue: the WorkACTIVE-P randomized controlled trial. *Applied Physiology, Nutrition, and Metabolism*. 2021;46(2):117-125.
108. Fayh APT, Lopes AL, da Silva AMV, Reischak-Oliveira A, Friedman R. Effects of 5% weight loss through diet or diet plus exercise on cardiovascular parameters of obese: a randomized clinical trial. *European journal of nutrition*. 2013;52:1443-1450.
109. Gepner Y, Shelef I, Schwarzfuchs D, et al. Effect of distinct lifestyle interventions on mobilization of fat storage pools: The CENTRAL MRI randomized controlled trial. *Circulation*. 2018;137(11):1143-1157.
110. Ko G, Davidson LE, Brennan AM, Lam M, Ross R. Abdominal adiposity, not cardiorespiratory fitness, mediates the exercise-induced change in insulin sensitivity in older adults. *PLoS One*. 2016;11(12):e0167734.
111. Layman DK, Evans E, Baum JI, Seyler J, Erickson DJ, Boileau RA. Dietary protein and exercise have additive effects on body composition during weight loss in adult women. *The Journal of nutrition*. 2005;135(8):1903-1910.
112. Legaard GE, Feineis CS, Johansen MY, et al. Effects of an exercise-based lifestyle intervention on systemic markers of oxidative stress and advanced glycation endproducts in persons with type 2 diabetes: Secondary analysis of a randomised clinical trial. *Free Radical Biology and Medicine*. 2022;188:328-336.
113. Leroux-Stewart J, Elisha B, Tagougui S, et al. Effect of caloric restriction with or without physical activity on body composition and epicardial fat in type 2 diabetic patients: A pilot randomized controlled trial. *Nutrition, Metabolism and Cardiovascular Diseases*. 2021;31(3):921-929.
114. Maleklou F, Hakakzadeh A, Halabchi F, Tabesh MR, Alizadeh Z. Effects of aerobic exercise concurrent with caffeine supplementation on weight and body fat among overweight women. *Asian Journal of Sports Medicine*. 2022;13(1)
115. McElfish PA, Felix HC, Bursac Z, et al. A Cluster Randomized Controlled Trial Comparing Diabetes Prevention Program Interventions for Overweight/Obese Marshallese Adults. *INQUIRY: The Journal of Health Care Organization, Provision, and Financing*. 2023;60:00469580231152051.
116. Mezghani N, Ammar A, Boukhris O, et al. The Impact of Exercise Training Intensity on Physiological Adaptations and Insulin Resistance in Women with Abdominal Obesity. MDPI; 2022:2533.

117. Mezghanni N, Chaabouni K, Chtourou H, et al. Effect of exercise training intensity on body composition, lipid profile, and insulin resistance in young obese women. *African Journal of Microbiology Research*. 2012;6(10):2481-2488.
118. Mohammadi G, Eskandari Z. Comparison of Chinese Acupressure with Traditional Iranian Medicine Along with Aerobic Exercise on Weight Loss and Serum Lipid Factors in Obese and Overweight Women. *Asian Journal of Sports Medicine*. 2022;13(4)
119. Montemayor S, Bouzas C, Mascaró CM, et al. Effect of dietary and lifestyle interventions on the amelioration of NAFLD in patients with metabolic syndrome: The FLIPAN study. *Nutrients*. 2022;14(11):2223.
120. Mourier A, Gautier J-F, Kerviler ED, et al. Mobilization of visceral adipose tissue related to the improvement in insulin sensitivity in response to physical training in NIDDM: effects of branched-chain amino acid supplements. *Diabetes care*. 1997;20(3):385-391.
121. Nakata Y, Okada M, Hashimoto K, Harada Y, Sone H, Tanaka K. Comparison of education-only versus group-based intervention in promoting weight loss: a randomised controlled trial. *Obesity Facts*. 2011;4(3):222-228.
122. Nam S, Stewart KJ, Dobrosielski DA. Lifestyle intervention for sleep disturbances among overweight or obese individuals. *Behavioral sleep medicine*. 2016;14(3):343-350.
123. Nordby P, Auerbach PL, Rosenkilde M, et al. Endurance training per se increases metabolic health in young, moderately overweight men. *Obesity*. 2012;20(11):2202-2212.
124. Oh D-H, Lee J-K. Effect of Different Intensities of Aerobic Exercise Combined with Resistance Exercise on Body Fat, Lipid Profiles, and Adipokines in Middle-Aged Women with Obesity. *International Journal of Environmental Research and Public Health*. 2023;20(5):3991.
125. Sandsdal RM, Juhl CR, Jensen SB, et al. Combination of exercise and GLP-1 receptor agonist treatment reduces severity of metabolic syndrome, abdominal obesity, and inflammation: a randomized controlled trial. *Cardiovascular diabetology*. 2023;22(1):41.
126. Wallace MB, Mills BD, Browning CL. Effects of cross-training on markers of insulin resistance/hyperinsulinemia. *Medicine and science in sports and exercise*. 1997;29:1170-1175.
127. Xue Q, Li X, Ma H, et al. Changes in pedometer-measured physical activity are associated with weight loss and changes in body composition and fat distribution in response to reduced-energy diet interventions: The POUNDS Lost trial. *Diabetes, Obesity and Metabolism*. 2022;24(6):1000-1009.
128. Zhang Y, Guo X, Zhang N, et al. Effect of mobile-based lifestyle intervention on body weight, glucose and lipid metabolism among the overweight and obese elderly population in China: a randomized controlled trial protocol. *International Journal of Environmental Research and Public Health*. 2021;18(9):4854.
129. 2022. Aerobic exercise versus mediterranean diet on insulin resistance in obese prediabetic postmenopausal women: A randomized controlled study. *Fizjoterapia Polska*. Hala Mohamed Hanafy, Magda Sayed Morsi, Hosam El-Din Hussain Kamel, Safaa Al-Hossany Tawfiq, Sally Osama Baraka,;22:184-190.
130. Ard JD, Carson TL, Shikany JM, et al. Weight loss and improved metabolic outcomes amongst rural African American women in the Deep South: six-month outcomes from a community-based randomized trial. *Journal of internal medicine*. 2017;282(1):102-113.
131. Bacchi E, Negri C, Targher G, et al. Both resistance training and aerobic training reduce hepatic fat content in type 2 diabetic subjects with nonalcoholic fatty liver disease (the RAED2 Randomized Trial). *Hepatology*. 2013;58(4):1287-1295.
132. Ballor DL, Harvey-Berino JR, Ades PA, Cryan J, Calles-Escandon J. Contrasting effects of resistance and aerobic training on body composition and metabolism after diet-induced weight loss. *Metabolism*. 1996;45(2):179-183.

© 2024 Jayedi A et al. *JAMA Network Open*.

133. Berge J, Hjelmesæth J, Hertel JK, et al. Effect of aerobic exercise intensity on energy expenditure and weight loss in severe obesity—a randomized controlled trial. *Obesity*. 2021;29(2):359-369.
134. Berge J, Hjelmesæth J, Kolotkin RL, et al. Effect of aerobic exercise intensity on health-related quality of life in severe obesity: a randomized controlled trial. *Health and Quality of Life Outcomes*. 2022;20(1):1-10.
135. Bragg AE, Crowe-White KM, Ellis AC, et al. Changes in cardiometabolic risk among older adults with obesity: an ancillary analysis of a randomized controlled trial investigating exercise plus weight maintenance and exercise plus intentional weight loss by caloric restriction. *Journal of the Academy of Nutrition and Dietetics*. 2022;122(2):354-362.
136. Choo J, Lee J, Cho J-H, Burke LE, Sekikawa A, Jae SY. Effects of weight management by exercise modes on markers of subclinical atherosclerosis and cardiometabolic profile among women with abdominal obesity: a randomized controlled trial. *BMC Cardiovascular Disorders*. 2014;14(1):1-13.
137. Collins KA, Fos LB, Ross LM, et al. Aerobic, resistance, and combination training on health-related quality of life: the STRRIDE-AT/RT randomized trial. *Frontiers in Sports and Active Living*. 2021;2:620300.
138. Fanning J, Rejeski WJ, Leng I, et al. Intervening on exercise and daylong movement for weight loss maintenance in older adults: a randomized, clinical trial. *Obesity*. 2022;30(1):85-95.
139. Figard-Fabre H, Fabre N, Leonardi A, Schena F. Efficacy of Nordic walking in obesity management. *International journal of sports medicine*. 2011:407-414.
140. Friedenreich CM, Neilson HK, O'Reilly R, et al. Effects of a high vs moderate volume of aerobic exercise on adiposity outcomes in postmenopausal women: a randomized clinical trial. *JAMA oncology*. 2015;1(6):766-776.
141. Jamka M, Mądry E, Bogdański P, et al. The effect of endurance and endurance-strength training on bone mineral density and content in abdominally obese postmenopausal women: A randomized trial. MDPI; 2021:1074.
142. Jeffery RW, Wing RR, Sherwood NE, Tate DF. Physical activity and weight loss: does prescribing higher physical activity goals improve outcome? *The American journal of clinical nutrition*. 2003;78(4):684-689.
143. Kambic T, Šarabon N, Lainscak M, Hadžić V. Combined resistance training with aerobic training improves physical performance in patients with coronary artery disease: a secondary analysis of a randomized controlled clinical trial. *Frontiers in cardiovascular medicine*. 2022;9:909385.
144. Keating SE, Hackett DA, Parker HM, et al. Effect of aerobic exercise training dose on liver fat and visceral adiposity. *Journal of hepatology*. 2015;63(1):174-182.
145. Lukoszek D, Sieroń D, Jabłońska I, Szczegielniak J, Trąbka R, Szyluk K. Efficacy of ultrasound in diagnosis and treatment of the shoulder-a systematic review. *Fizjoterapia Polska*. 2022;22(4)
146. Madrid DA, Beavers KM, Walkup MP, et al. Effect of exercise modality and weight loss on changes in muscle and bone quality in older adults with obesity. *Experimental Gerontology*. 2023;174:112126.
147. Oliveira GTA, Costa EC, Santos TM, et al. Effect of High-Intensity Interval, Moderate-Intensity Continuous, and Self-Selected Intensity Training on Health and Affective Responses. *Research Quarterly for Exercise and Sport*. 2023:1-16.
148. Purcell SA, Legget KT, Halliday TM, et al. Appetitive and Metabolic Responses to an Exercise versus Dietary Intervention in Adults with Obesity. *Translational Journal of the American College of Sports Medicine*. 2022;7(4):e000211.
149. Sabag A, Way KL, Sultana RN, et al. The effect of a novel low-volume aerobic exercise intervention on liver fat in type 2 diabetes: a randomized controlled trial. *Diabetes Care*. 2020;43(10):2371-2378.
150. Sasai H, Katayama Y, Nakata Y, et al. The effects of vigorous physical activity on intra-abdominal fat levels: a preliminary study of middle-aged Japanese men. *Diabetes research and clinical practice*. 2010;88(1):34-41.

© 2024 Jayedi A et al. *JAMA Network Open*.

151. Senkus KE, Crowe-White KM, Bolland AC, Locher JL, Ard JD. Changes in adiponectin: leptin ratio among older adults with obesity following a 12-month exercise and diet intervention. *Nutrition & Diabetes*. 2022;12(1):30.
152. Stillman CM, Jakicic J, Rogers R, et al. Changes in cerebral perfusion following a 12-month exercise and diet intervention. *Psychophysiology*. 2021;58(7):e13589.
153. Taha MM, Aneis YM, Mohamady HM, Alrasheedy S, Elsayed SH. Effect of focused ultrasound cavitation augmented with aerobic exercise on abdominal and intrahepatic fat in patients with non-alcoholic fatty liver disease: A randomized controlled trial. *Plos one*. 2021;16(4):e0250337.
154. Von Korn P, Keating S, Mueller S, et al. The effect of exercise intensity and volume on metabolic phenotype in patients with metabolic syndrome: a randomized controlled trial. *Metabolic syndrome and related disorders*. 2021;19(2):107-114.
155. Youssef M. Effects of moderate exercise versus light exercise on fasting blood glucose in obese patients with type 2 diabetes mellitus. *Physiotherapy Quarterly*. 31(3)
156. Zeng J, Peng L, Zhao Q, Chen Q. Effects over 12 weeks of different types and durations of exercise intervention on body composition of young women with obesity. *Science & Sports*. 2021;36(1):45-52.
157. Abdelbasset WK, Elsayed SH, Nambi G, et al. Effect of moderate-intensity aerobic exercise on hepatic fat content and visceral lipids in hepatic patients with diabesity: a single-blinded randomised controlled trial. *Evidence-Based Complementary and Alternative Medicine*. 2020;2020
158. Andreou E, Philippou C, Papandreou D. Effects of an intervention and maintenance weight loss diet with and without exercise on anthropometric indices in overweight and obese healthy women. *Annals of Nutrition and Metabolism*. 2011;59(2-4):187-192.
159. Asjari M, Abedi B, Fatolahi H. Effects of aerobic training and licorice extract consumption on inflammation and antioxidant states in overweight women. *Obesity Medicine*. 2021;21:100271.
160. Bittman J, Thomson CJ, Lyall LA, et al. Effect of an Exercise and Nutrition Program on Quality of Life in Patients With Atrial Fibrillation: The Atrial Fibrillation Lifestyle Project (ALP). *CJC open*. 2022;4(8):685-694.
161. Borg P, Kukkonen-Harjula K, Fogelholm M, Pasanen M. Effects of walking or resistance training on weight loss maintenance in obese, middle-aged men: a randomized trial. *International journal of obesity*. 2002;26(5):676-683.
162. Chen CK, Ismail NS, Al-Safi AA. Effects of brisk walking and resistance training on cardiorespiratory fitness, body composition, and lipid profiles among overweight and obese individuals. *Journal of Physical Education and Sport*. 2016;16(3):957.
163. Fritz T, Caidahl K, Osler M, Östenson C, Zierath J, Wändell P. Effects of Nordic walking on health-related quality of life in overweight individuals with Type 2 diabetes mellitus, impaired or normal glucose tolerance. *Diabetic Medicine*. 2011;28(11):1362-1372.
164. Gepner Y, Shelef I, Schwarzfuchs D, et al. Effect of distinct lifestyle interventions on mobilization of fat storage pools: CENTRAL magnetic resonance imaging randomized controlled trial. *Circulation*. 2018;137(11):1143-1157.
165. Guessogo WR, Temfemo A, Mandengue SH, et al. Effect of 24-week repeated short-time walking based training program on physical fitness of black Cameroonian obese women. *Journal of exercise rehabilitation*. 2016;12(2):90.
166. Ismail AMA, Tolba AMN, Felaya E-SEE-S. Effect of aerobic exercise training on leptin and liver enzymes in non-diabetic overweight hepatitis C men. *Postepy Rehabilitacji*. 2021;35(2):17.
167. Jakicic JM, Rogers RJ, Lang W, et al. Impact of weight loss with diet or diet plus physical activity on cardiac magnetic resonance imaging and cardiovascular disease risk factors: Heart Health Study randomized trial. *Obesity*. 2022;30(5):1039-1056.

© 2024 Jayedi A et al. *JAMA Network Open*.

168. Lakhdar N, Denguezli M, Zaouali M, Zbidi A, Tabka Z, Bouassida A. Diet and diet combined with chronic aerobic exercise decreases body fat mass and alters plasma and adipose tissue inflammatory markers in obese women. *Inflammation*. 2013;36(6):1239-1247.
169. Lugones-Sánchez C, Recio-Rodríguez JI, Menéndez-Suárez M, et al. Effect of a Multicomponent mHealth Intervention on the Composition of Diet in a Population with Overweight and Obesity—Randomized Clinical Trial EVIDENT 3. *Nutrients*. 2022;14(2):270.
170. McNeilly AM, Davison GW, Murphy MH, et al. Effect of α-lipoic acid and exercise training on cardiovascular disease risk in obesity with impaired glucose tolerance. *Lipids in health and disease*. 2011;10:1-9.
171. Quist JS, Rosenkilde M, Petersen M, Gram A, Sjödin A, Stallknecht B. Effects of active commuting and leisure-time exercise on fat loss in women and men with overweight and obesity: a randomized controlled trial. *International Journal of Obesity*. 2018;42(3):469-478.
172. Sullivan S, Kirk EP, Mittendorfer B, Patterson BW, Klein S. Randomized trial of exercise effect on intrahepatic triglyceride content and lipid kinetics in nonalcoholic fatty liver disease. *Hepatology*. 2012;55(6):1738-1745.
173. Adams-Campbell LL, Taylor T, Hicks J, Lu J, Dash C. The effect of a 6-month exercise intervention trial on allostatic load in Black women at increased risk for breast cancer: The FIERCE study. *Journal of Racial and Ethnic Health Disparities*. 2021:1-7.
174. Beavers KM, Ambrosius WT, Nicklas BJ, Rejeski WJ. Independent and combined effects of physical activity and weight loss on inflammatory biomarkers in overweight and obese older adults. *Journal of the American Geriatrics Society*. 2013;61(7):1089-1094.
175. Collins KA, Kraus WE, Rogers RJ, et al. Effect of behavioral weight-loss program on biomarkers of cardiometabolic disease risk: Heart Health Study randomized trial. *Obesity*. 2023;31(2):338-349.
176. Elsayed M, Rabiee A, Elrefaye G, Elsisi H. Effect of aerobic exercise with diet on sex hormones and selected coagulation biomarkers in obese postmenopausal women: a randomized clinical trial. *European Review for Medical & Pharmacological Sciences*. 2022;26(2)
177. Ho E, Qualls C, Villareal DT. Effect of diet, exercise, or both on biological age and healthy aging in older adults with obesity: secondary analysis of a randomized controlled trial. *The journal of nutrition, health & aging*. 2022;26(6):552-557.
178. Janssen I, Fortier A, Hudson R, Ross R. Effects of an energy-restrictive diet with or without exercise on abdominal fat, intermuscular fat, and metabolic risk factors in obese women. *Diabetes care*. 2002;25(3):431-438.
179. Katzel LI, Bleecker ER, Colman EG, Rogus EM, Sorkin JD, Goldberg AP. Effects of weight loss vs aerobic exercise training on risk factors for coronary disease in healthy, obese, middle-aged and older men: a randomized controlled trial. *Jama*. 1995;274(24):1915-1921.
180. Messier SP, Newman JJ, Scarlett MJ, et al. Changes in Body Weight and Knee Pain in Adults With Knee Osteoarthritis Three-and-a-Half Years After Completing Diet and Exercise Interventions: Follow-Up Study for a Single-Blind, Single-Center, Randomized Controlled Trial. *Arthritis Care & Research*. 2022;74(4):607-616.
181. Nieman DC, Brock DW, Butterworth D, Utter AC, Nieman CC. Reducing diet and/or exercise training decreases the lipid and lipoprotein risk factors of moderately obese women. *Journal of the American College of Nutrition*. 2002;21(4):344-350.
182. Pugh CJ, Cuthbertson DJ, Sprung VS, et al. Exercise training improves cutaneous microvascular function in nonalcoholic fatty liver disease. *American Journal of Physiology-Endocrinology and Metabolism*. 2013;305(1):E50-E58.
183. Rejeski WJ, Ambrosius WT, Burdette JH, Walkup MP, Marsh AP. Community weight loss to combat obesity and disability in at-risk older adults. *Journals of Gerontology Series A: Biomedical Sciences and Medical Sciences*. 2017;72(11):1547-1553.

© 2024 Jayedi A et al. *JAMA Network Open*.

184. Rejeski WJ, Brubaker PH, Goff DC, et al. Translating weight loss and physical activity programs into the community to preserve mobility in older, obese adults in poor cardiovascular health. *Archives of internal medicine*. 2011;171(10):880-886.
185. Waters DL, Aguirre L, Gurney B, et al. Effect of aerobic or resistance exercise, or both, on intermuscular and visceral fat and physical and metabolic function in older adults with obesity while dieting. *The Journals of Gerontology: Series A*. 2022;77(1):131-139.
186. Bonab SB, Parvaneh M. Effect of 12-week of aerobic exercise on hormones and lipid profile status in adolescent girls with polycystic ovary syndrome: A study during COVID-19. *Science & Sports*. 2023;38(5-6):565-573.
187. Keller C, Treviño RP. Effects of two frequencies of walking on cardiovascular risk factor reduction in Mexican American women §. *Research in nursing & health*. 2001;24(5):390-401.
188. Kim M-K, Tomita T, Kim M-J, Sasai H, Maeda S, Tanaka K. Aerobic exercise training reduces epicardial fat in obese men. *Journal of Applied Physiology*. 2009;106(1):5-11.
189. Mardani S, Binias SA, Ramezani S. The Effect of 8 Weeks of Continuous Aerobic Training on Serum Irizin Level and Insulin Resistance Index of Middle-Aged Women with Type 2 Diabetes. *Iranian Journal of Diabetes and Metabolism*. 2022;22(2):89-98.
190. Miyatake N, Takahashi K, Wada J, et al. Daily exercise lowers blood pressure and reduces visceral adipose tissue areas in overweight Japanese men. *Diabetes research and clinical practice*. 2003;62(3):149-157.
191. Mohammadkhani PG, Irandoust K, Taheri M, Mirmoezzi M, Baić M. Effects of eight weeks of aerobic exercise and taking caraway supplement on C-reactive protein and sleep quality in obese women. *Biological Rhythm Research*. 2021;52(2):218-226.
192. Moss SJ. Changes in coronary heart disease risk profile of adults with intellectual disabilities following a physical activity intervention. *Journal of Intellectual Disability Research*. 2009;53(8):735-744.
193. Musto A, Jacobs K, Nash M, DelRossi G, Perry A. The effects of an incremental approach to 10,000 steps/day on metabolic syndrome components in sedentary overweight women. *Journal of physical activity and health*. 2010;7(6):737-745.
194. Rajabi A, Khajehlandi M, Siahkuhian M, Akbarnejad A, Khoramipour K, Suzuki K. Effect of 8 weeks aerobic training and saffron supplementation on inflammation and metabolism in middle-aged obese women with type 2 diabetes mellitus. *Sports*. 2022;10(11):167.
195. Setiakarnawijaya Y, Fitranto N, Taufik MS, Hanief YN. Effect of aerobic exercise on stress reduction and weight loss in obese students using circuit training. *Journal of Physical Education and Sport*. 2022;22(12):3038-3043.
196. Soori S, Heirani A, Rafie F. Effects of the aerobic and Pilates exercises on mental health in inactive older women. *Journal of Women & Aging*. 2022;34(4):429-437.
197. Yaghubi Z, ABEDI B. Effect of Eight Weeks of Aerobic Exercise with Ginger Supplementation on FGF21, Irisin and Insulin Resistance in Women with Type 2 Diabetes. 2021;
198. Koeder C, Kranz R-M, Anand C, et al. Effect of a 1-year controlled lifestyle intervention on body weight and other risk markers (the healthy lifestyle community programme, cohort 2). *Obesity Facts*. 2022;15(2):228-239.
199. Kuk JL. Associations between abdominal adiposity, exercise, morbidity, and mortality. *Applied Physiology, Nutrition, and Metabolism*. 2007;32(6):1210-1211.
200. Park S-K, Park J-H, Kwon Y-C, Kim H-S, Yoon M-S, Park H-T. The effect of combined aerobic and resistance exercise training on abdominal fat in obese middle-aged women. *Journal of physiological anthropology and applied human science*. 2003;22(3):129-135.
201. Alvarez C, Ramirez-Campillo R, Martinez-Salazar C, et al. Low-volume high-intensity interval training as a therapy for type 2 diabetes. *International journal of sports medicine*. 2016:723-729.

© 2024 Jayedi A et al. *JAMA Network Open*.

202. Batrakoulis A, Jamurtas AZ, Georgakouli K, et al. High intensity, circuit-type integrated neuromuscular training alters energy balance and reduces body mass and fat in obese women: A 10-month training-detraining randomized controlled trial. *PloS one*. 2018;13(8):e0202390.
203. Batrakoulis A, Loules G, Georgakouli K, et al. High-intensity interval neuromuscular training promotes exercise behavioral regulation, adherence and weight loss in inactive obese women. *European journal of sport science*. 2020;20(6):783-792.
204. Blue MN, Smith-Ryan AE, Trexler ET, Hirsch KR. The effects of high intensity interval training on muscle size and quality in overweight and obese adults. *Journal of science and medicine in sport*. 2018;21(2):207-212.
205. Heydari M, Freund J, Boutcher SH. The effect of high-intensity intermittent exercise on body composition of overweight young males. *Journal of obesity*. 2012;2012
206. Hsu KJ, Chien KY, Tsai SC, et al. Effects of Exercise Alone or in Combination with High-Protein Diet on Muscle Function, Aerobic Capacity, and Physical Function in Middle-Aged Obese Adults: A Randomized Controlled Trial. *J Nutr Health Aging*. 2021;25(6):727-734. doi:10.1007/s12603-021-1599-1
207. Ismail AMA, El-Azeim ASA, Saif HFAEA. Effect of aerobic exercise alone or combined with Mediterranean diet on dry eye in obese hypertensive elderly. *Irish Journal of Medical Science (1971-)*. 2023:1-11.
208. Mora-Rodriguez R, Fernández-Elías VE, Morales-Palomo F, Pallares J, Ramirez-Jimenez M, Ortega JF. Aerobic interval training reduces vascular resistances during submaximal exercise in obese metabolic syndrome individuals. *European journal of applied physiology*. 2017;117:2065-2073.
209. Nikseresht M, Agha-Alinejad H, Azarbayjani MA, Ebrahim K. Effects of nonlinear resistance and aerobic interval training on cytokines and insulin resistance in sedentary men who are obese. *The Journal of Strength & Conditioning Research*. 2014;28(9):2560-2568.
210. Reljic D, Frenk F, Herrmann HJ, Neurath MF, Zopf Y. Effects of very low volume high intensity versus moderate intensity interval training in obese metabolic syndrome patients: a randomized controlled study. *Scientific reports*. 2021;11(1):2836.
211. Stensvold D, Tjønna AE, Skaug E-A, et al. Strength training versus aerobic interval training to modify risk factors of metabolic syndrome. *Journal of applied physiology*. 2010;108(4):804-810.
212. Valsdottir TD, Øvrebø B, Kornfeldt TM, et al. Effect of aerobic exercise and low-carbohydrate high-fat diet on glucose tolerance and android/gynoid fat in overweight/obese women: A randomized controlled trial. *Frontiers in Physiology*. 2023;14:1056296.
213. Bennet L, Fawad A, Struck J, Larsson SL, Bergmann A, Melander O. The effect of a randomised controlled lifestyle intervention on weight loss and plasma proneurotensin. *BMC Endocrine Disorders*. 2022;22(1):264.
214. Carneiro-Barrera A, Amaro-Gahete FJ, Guillén-Riquelme A, et al. Effect of an interdisciplinary weight loss and lifestyle intervention on obstructive sleep apnea severity: the INTERAPNEA randomized clinical trial. *JAMA Network Open*. 2022;5(4):e228212-e228212.
215. Deraas TS, Hopstock L, Henriksen A, et al. Complex lifestyle intervention among inactive older adults with elevated cardiovascular disease risk and obesity: a mixed-method, single-arm feasibility study for RESTART—a randomized controlled trial. *Pilot and Feasibility Studies*. 2021;7:1-19.
216. Díaz-López A, Becerra-Tomás N, Ruiz V, et al. Effect of an intensive weight-loss lifestyle intervention on kidney function: a randomized controlled trial. *American Journal of Nephrology*. 2021;52(1):45-58.
217. Drew RJ, Morgan PJ, Collins CE, et al. Behavioral and cognitive outcomes of an online weight loss program for men with low mood: a randomized controlled trial. *Annals of Behavioral Medicine*. 2022;56(10):1026-1041.

© 2024 Jayedi A et al. *JAMA Network Open*.

218. Fawcett K, Martinez A, Crimmins M, Sims C, Børsheim E, Andres A. Effect of a dietary and exercise intervention in women with overweight and obesity undergoing fertility treatments: protocol for a randomized controlled trial. *BMC nutrition*. 2021;7:1-12.
219. Georgoulis M, Yiannakouris N, Kechribari I, et al. The effectiveness of a weight-loss Mediterranean diet/lifestyle intervention in the management of obstructive sleep apnea: Results of the "MIMOSA" randomized clinical trial. *Clinical Nutrition*. 2021;40(3):850-859.
220. Lockard B, Mardock M, Oliver JM, et al. Comparison of Two Diet and Exercise Approaches on Weight Loss and Health Outcomes in Obese Women. *International Journal of Environmental Research and Public Health*. 2022;19(8):4877.
221. Rani GS, Swaminathan A. Effectiveness Of Physical Activity And Diet Supplementation On Body Mass Index And Bone Mineral Density Among Premenopausal Women. *Journal of Pharmaceutical Negative Results*. 2022:4403-4411.
222. Silva AM, Nunes CL, Jesus F, et al. Effectiveness of a lifestyle weight-loss intervention targeting inactive former elite athletes: the Champ4Life randomised controlled trial. *British Journal of Sports Medicine*. 2022;56(7):394-402.
223. Blonk M, Jacobs M, Biesheuvel E, Weeda-Mannak W, Heine R. Influences on weight loss in Type 2 diabetic patients: little long-term benefit from group behaviour therapy and exercise training. *Diabetic Medicine*. 1994;11(5):449-457.
224. Gerosa-Neto J, Panissa VLG, Monteiro PA, et al. High-or moderate-intensity training promotes change in cardiorespiratory fitness, but not visceral fat, in obese men: A randomised trial of equal energy expenditure exercise. *Respiratory physiology & neurobiology*. 2019;266:150-155.
225. Hu M, Shi Q, Sun S, et al. Effect of a Low-Carbohydrate Diet With or Without Exercise on Anxiety and Eating Behavior and Associated Changes in Cardiometabolic Health in Overweight Young Women. *Frontiers in Nutrition*. 2022;9:894916.
226. Johnson NA, Sachinwalla T, Walton DW, et al. Aerobic exercise training reduces hepatic and visceral lipids in obese individuals without weight loss. *Hepatology*. 2009;50(4):1105-1112.
227. Koh Y, Park K-S. Responses of inflammatory cytokines following moderate intensity walking exercise in overweight or obese individuals. *Journal of exercise rehabilitation*. 2017;13(4):472.
228. Mahdirejei TA, Razi M, Barari A, et al. A comparative study of the effects of endurance and resistance exercise training on PON1 and lipid profile levels in obese men. *Sport Sciences for Health*. 2015;11:263-270.
229. Nakhaei H, Nayebifar S, Fanaei H. Decrease in serum asprosin levels following six weeks of spinning and stationary cycling training in overweight women. *Hormone molecular biology and clinical investigation*. 2022;44(1):21-26.
230. Park K-S, Nickerson BS. Aerobic exercise is an independent determinant of levels of inflammation and oxidative stress in middle-aged obese females. *Journal of exercise rehabilitation*. 2022;18(1):43.
231. Shojaee-Moradie F, Baynes K, Pentecost C, et al. Exercise training reduces fatty acid availability and improves the insulin sensitivity of glucose metabolism. *Diabetologia*. 2007;50:404-413.
232. Sun S, Kong Z, Shi Q, Zhang H, Lei O-K, Nie J. Carbohydrate restriction with or without exercise training improves blood pressure and insulin sensitivity in overweight women. MDPI; 2021:637.
233. Winn NC, Liu Y, Rector RS, Parks EJ, Ibdah JA, Kanaley JA. Energy-matched moderate and high intensity exercise training improves nonalcoholic fatty liver disease risk independent of changes in body mass or abdominal adiposity—a randomized trial. *Metabolism*. 2018;78:128-140.
234. Alizadeh Z, Kordi R, Attar MJH-Z, Mansournia MA. The effects of continuous and intermittent aerobic exercise on lipid profile and fasting blood sugar in women with a body mass index more than 25 kg/m 2: a randomized controlled trial. *Tehran University Medical Journal*. 2011;69(4)
235. Coquart J, Lemaire C, Douillard C, Garcin M. Effects of intermittent walk program on the body mass and composition in obese women. 2008:227-230.

© 2024 Jayedi A et al. *JAMA Network Open*.

236. Soori R, Asad MR, Khosravi M, Abbasian S. Comparison of the effects of regular aerobic training irisin serum changes in sedentary obese men. *Iranian Journal of Endocrinology and Metabolism*. 2016;18(4):270-278.
237. Yari S, Taheri M, Irandoust K. The effect of omega-3 fatty acid supplementation and aerobic exercise on the depression of obese women. *EBNESINA*. 2018;20(2):30-35.
238. Chen Y, Chen Z, Pan L, et al. Effect of moderate and vigorous aerobic exercise on incident diabetes in adults with obesity: a 10-year follow-up of a randomized clinical trial. *JAMA Internal Medicine*. 2023;183(3):272-275.
239. Hunter GR, Brock DW, Byrne NM, Chandler-Laney PC, Del Corral P, Gower BA. Exercise training prevents regain of visceral fat for 1 year following weight loss. *Obesity*. 2010;18(4):690-695.
240. Levakov G, Kaplan A, Meir AY, et al. The effect of weight loss following 18 months of lifestyle intervention on brain age assessed with resting-state functional connectivity. *Elife*. 2023;12:e83604.
241. Couillard C, Després J-P, Lamarche B, et al. Effects of endurance exercise training on plasma HDL cholesterol levels depend on levels of triglycerides: evidence from men of the Health, Risk Factors, Exercise Training and Genetics (HERITAGE) Family Study. *Arteriosclerosis, thrombosis, and vascular biology*. 2001;21(7):1226-1232.
242. Lee S, Kuk JL, Davidson LE, et al. Exercise without weight loss is an effective strategy for obesity reduction in obese individuals with and without Type 2 diabetes. *Journal of applied physiology*. 2005;
243. Bharath LP, Choi WW, Cho J-m, et al. Combined resistance and aerobic exercise training reduces insulin resistance and central adiposity in adolescent girls who are obese: randomized clinical trial. *European journal of applied physiology*. 2018;118:1653-1660.
244. Comeras-Chueca C, Villalba-Heredia L, Perez-Lasierra JL, et al. Effect of an active video game intervention combined with multicomponent exercise for cardiorespiratory fitness in children with overweight and obesity: randomized controlled trial. *JMIR Serious Games*. 2022;10(2):e33782.
245. Bertz F, Winkvist A, Brekke HK. Sustainable weight loss among overweight and obese lactating women is achieved with an energy-reduced diet in line with dietary recommendations: results from the LEVA randomized controlled trial. *Journal of the Academy of Nutrition and Dietetics*. 2015;115(1):78-86.

© 2024 Jayedi A et al. *JAMA Network Open*.
