# Supplementary material for: Aerobic Exercise and Weight Loss in Adults: A Systematic Review and Dose-Response Meta-Analysis
Source: JAMA Netw Open. 2024 Dec 26;7(12):e2452185. doi: 10.1001/jamanetworkopen.2024.52185 (PMC11672165; doi:10.1001/jamanetworkopen.2024.52185)
Supplement: Supplement 2. — Data Sharing Statement [file jamanetwopen-e2452185-s002.pdf]

Data Sharing Statement

Jayedi. Aerobic Exercise and Weight Loss In Adults. *JAMA Netw Open*. Published December 26, 2024. doi:10.1001/jamanetworkopen.2024.52185

Data
Data available: No

Additional Information
Explanation for why data not available: Additional Information Explanation for why data not available: All extracted and calculated data are available by emailing to the corresponding author on reasonable request.
